# The Polycyclic Aromatic Hydrocarbon (PAH) degradation activities and genome analysis of a novel strain *Stenotrophomonas sp*. Pemsol isolated from Mexico

**DOI:** 10.7717/peerj.8102

**Published:** 2020-01-06

**Authors:** Temidayo O. Elufisan, Isabel C. Rodríguez-Luna, Omotayo Opemipo Oyedara, Alejandro Sánchez-Varela, Armando Hernández-Mendoza, Edgar Dantán Gonzalez, Alma D. Paz-González, Kashif Muhammad, Gildardo Rivera, Miguel Angel Villalobos-Lopez, Xianwu Guo

**Affiliations:** 1 Laboratorio de Biotecnologia Genomica, Centro de Biotecnologia Genomica, Instituto Politecnico Nacional, Mexico, Reynosa, Tamaulipas, Mexico; 2 Science Policy and Innovation Studies (SPIS), National Center for Technology Management Obafemi Awolowo University campus Ile-Ife, Ile-Ife, Osun, Nigeria; 3 Microbiology Department, Osun State University, Osogbo, Osun, Nigeria; 4 Centro de Investigación en Dinámica Celular, Instituto de Investigación en Ciencias Básicas y Aplicadas, Universidad Autónoma del Estado de Morelos (UAEM), Cuernavaca, Morelos, Mexico; 5 Laboratorio de Estudios Ecogenómicos (UAEM), Centro de Investigación en Biotecnología, Universidad Autónoma del Estado de Morelos, Cuernavaca, Morelos, Mexico; 6 Laboratorio de Biotecnologia Famaceutica, Centro de Biotecnologia Genomica, Instituto Politecnico Nacional, Mexico, Reynosa, Tamaulipas, Mexico; 7 Centro de Investigación en Biotecnología Aplicada, Instituto Politécnico Nacional, Tepetitla, Tlaxcala, Mexico

**Keywords:** *Stenotrophomonas*, Sequencing., Biphenyl, Polycyclic Aromatic Hydrocarbon (PAH), Naphthalene, Degradation

## Abstract

**Background:**

*Stenotrophomonas* are ubiquitous gram-negative bacteria, which can survive in a wide range of environments. They can use many substances for their growth and are known to be intrinsically resistant to many antimicrobial agents. They have been tested for biotechnological applications, bioremediation, and production of antimicrobial agents.

**Method:**

*Stenotrophomonas sp*. Pemsol was isolated from a crude oil contaminated soil. The capability of this isolate to tolerate and degrade polycyclic aromatic hydrocarbons (PAH) such as anthraquinone, biphenyl, naphthalene, phenanthrene, phenanthridine, and xylene was evaluated in Bushnell Hass medium containing PAHs as the sole carbon sources. The metabolites formed after 30-day degradation of naphthalene by Pemsol were analyzed using Fourier Transform Infra-red Spectroscopic (FTIR), Ultra-Performance Liquid Chromatography-Mass Spectrometry (UPLC-MS) and Gas Chromatography-Mass Spectrometry (GC-MS). The genome of Pemsol was also sequenced and analyzed.

**Results:**

Anthraquinone, biphenyl, naphthalene, phenanthrene, and phenanthridine except xylene can be used as sole carbon sources for Pemsol’s growth in Bushnell Hass medium. The degradation of naphthalene at a concentration of 1 mg/mL within 30 days was tested. A newly formed catechol peak and the disappearance of naphthalene peak detected on the UPLC-MS, and GC-MS analyses spectra respectively confirmed the complete degradation of naphthalene. Pemsol does not produce biosurfactant and neither bio-emulsify PAHs. The whole genome was sequenced and assembled into one scaffold with a length of 4,373,402 bp. A total of 145 genes involved in the degradation of PAHs were found in its genome, some of which are Pemsol-specific as compared with other 11 *Stenotrophomonas* genomes. Most specific genes are located on the genomic islands. *Stenotrophomonas* sp. Pemsol’s possession of few genes that are associated with bio-emulsification gives the genetic basis for its inability to bio-emulsify PAH. A possible degradation pathway for naphthalene in Pemsol was proposed following the analysis of Pemsol’s genome. ANI and GGDH analysis indicated that Pemsol is likely a new species of *Stenotrophomonas.* It is the first report on a complete genome sequence analysis of a PAH*-*degrading *Stenotrophomonas*. *Stenotrophomonas* sp. Pemsol possesses features that make it a good bacterium for genetic engineering and will be an excellent tool for the remediation of crude oil or PAH-contaminated soil.

## Introduction

*Stenotrophomonas* are ubiquitous bacteria, occupying various habitats, including harsh environments (Ryan et al., 2009; Hughes et al., 2016). They can use a wide range of substances for their growth (Juhasz, Stanley & Britz, 2000; Pages et al., 2008; Zhang et al., 2009; Urszula et al., 2009; Iyer, Iken & Leon, 2016). The vast metabolic capability of *Stenotrophomonas* species has encouraged various studies which aimed at find new paths for their biotechnological application, such as bioremediation, biodegradation, plant growth promotion, removal of organophosphate and synthesis of new antimicrobial agents (Ryan et al., 2009; Rajkumar et al., 2010; Iyer, Iken & Leon, 2016; Arulazhagan et al., 2017). In particular, several studies have focused on the use of *Stenotrophomonas maltophilia* for the remediation of Polycyclic Aromatic Hydrocarbons (PAHs) or crude oil contaminated sites (Boonchan, Britz & Stanley, 1998; Juhasz, Stanley & Britz, 2000; Arulazhagan et al., 2017).

PAHs are the compounds formed from two or more fused aromatic rings. PAHs often get into released into the environment either through natural or manmade combustion sources. PAHs range from naphthalene (two fused benzene rings) to coronene (seven fused benzene rings). Accidental petroleum spillage is one of the ways through which PAHs get into the environment. Human exposure to PAH or its analogs in the environment poses a high risk to health. Cancer resulting from previous exposure to PAHs has been demonstrated in animal models (Kim et al., 2013). Risks associated with PAH exposure validate the importance of an adequate cleanup strategy of a PAH polluted environment. Microbes are said to be the best agents for the bioremediation in oil-spilled sites (Haritash & Kaushik, 2009).

The degradation of PAH by bacteria is usually via the activities of some enzymes such as oxygenases and peroxidases. These enzymes include, but not limited to, alkane monooxygenases, such as AlkB from *Pseudomonas*, Alkm from *Acinetobacter* sp. Strain, ADP-1, AlkB1 and AlkB2 from *Rhodococcus* sp; XylE, catechol 2, 3 dioxygenases from *Pseudomonas putida*; NdoB, naphthalene monooxygenase from *P. putida*; and Nid*A*, pyrene dioxygenase large subunit from *Mycobacterium* sp. strain PYR-1, as well as various dehydrogenases and protocatechuate dioxygenases in *Stenotrophomonas* spp. (GUNSALUS, 1951; Seo, Keum & Li, 2009; Urszula et al., 2009; Das & Chandran, 2011). Bacterial degradation of PAHs can also involve the production of bio-surfactants (Van Beilen & Funhoff, 2007; Fritsche & Hofrichter, 2001). Biosurfactants or surface-active substances decrease the surface tension of water molecules, thereby making entrapped PAH on surfaces available for the use of bacteria (Boonchan, Britz & Stanley, 1998).

Genome sequencing has been used to explain detailed characteristics of many bacteria including their metabolic behavior (Makarova et al., 2001; Oyedara et al., 2018). Genome sequencing of some bacteria with the potentials to degrade hydrocarbons and PAHs has given profound insight into the genes involved in the degradation and mineralization of PAHs (Gunsalus, 1951; Schneiker et al., 2006; Kim et al., 2008, Das & Chandran, 2011; Pal et al., 2017). This genome sequence analysis has also provided information on the peripheral pathways associated with the PAH degradation process by bacteria including *Stenotrophomonas* species (Elufisan et al., 2019). Several bacteria with good potentials for hydrocarbon degradation have been sequenced (Kim et al., 2008; Das et al., 2015; Pal et al., 2017). Although there were reports of PAHs-degrading *Stenotrophomonas* strains, no such genome was sequenced and analyzed so far.

In this study, *Stenotrophomonas* sp. Pemsol isolated from crude oil-contaminated soil in the state of Tabasco, Mexico was evaluated for its ability to tolerate and degrade PAH as the sole carbon source in a minimal medium. The genome analysis of *Stenotrophomonas* sp. Pemsol revealed that it has many genes that are responsible for the degradation of PAHs and other hydrocarbons. The aim of this study is to elucidate and understand the genetic basis involved in the uptake and degradation of PAHs in *Stenotrophomonas* sp. Pemsol.

## Material & Methods

### Sampling, isolation, and cultivation of *Stenotrophomonassp*. Pemsol

*Stenotrophomonas sp.* Pemsol was isolated from a crude oil-contaminated soil, Tabasco, Mexico (17°52′26.9″N 92°29′12.4″W). One gram of soil sample was added into 10 mL of Luria-Bertani broth, and the mix was incubated at 30 °C overnight. one mL of the bacterial culture was serially diluted from 10^−1^to 10^−8^ in phosphate buffer (pH = 6.5). One hundred microliters of each dilution were spread on selective medium (StenoVIA agar, Himedia, India) plates. Colonies formed on plates were selected for further identification.

### Biochemical characterization

Biochemical tests were carried out based on the Bergey’s manual of determinative Bacteriological studies.

#### Sugar utilization test

The use of various sugars as a sole source of carbon was evaluated in liquid medium containing the test sugar as the only source of carbon. The liquid medium contains 2 g peptone, 5 g, Sodium chloride (NaCl) 0.3 g dibasic potassium phosphate (K_2_HPO_4_), 3 mg bromocresol purple, 10 g sugar in 1 liter of distilled water. Isolates ability to use different sugars as a sole carbon source were determined by the color changed in the liquid culture from purple to yellow following incubation for 24–48 h at 35 °C. * E. coli* ATCC (8739), *Pseudomonas aeruginosa* (ATCC 27853) and uninoculated sugar medium were used as control in the experimental set-up.

#### Other biochemical tests

Oxidase test was performed by using a commercially available oxidase test kit (Sigma Aldrich, USA). Catalase activity was determined by immersing a loopful of an isolate in hydrogen peroxides. Bile Aesculin hydrolysis was determined by streaking bacteria on Bile Aesculin plate and incubated at 37° C for 24 h. Decarboxylase and Deaminase enzyme activity was evaluated by inoculating isolates in basal medium with 1% amino acid and Lactose (Lysine, Serine, Ornithine). Decarboxylase tests were implemented as described by Elston (1971). Urease activity was evaluated as described by Brink (2010). *Proteus mirabilis* CDBB-B-1343(ATCC 21100) was used as positive control while *E* coli DH5 *α* was used as negative control for the analysis. Gelatin hydrolysis was evaluated in a gelatin agar plate containing nutrient agar and 0.8% gelatin. The gelatin agar was inoculated with a loopful of suspected *Stenotrophomonas* culture and incubated at 30 °C for 24 h. Hydrolysis of starch was tested on nutrient agar as described by Tindall (1990). Hydrolysis of Tween 80 was analyzed using the method described by Sierra (1957).

### Preliminary identification of isolates based on 16S RNA gene sequence and phylogenetic analysis

Genomic DNA was extracted from five mL bacterial culture grown in Luria broth using Promega wizard genomic DNA purification kit (Promega, Madison, USA) as per the manufacturer’s instruction. The 16S rRNA genes were amplified by PCR using steno1 (5′AGG GAA ACT TAC GCT AAT ACC- 3′) and steno2 (5′CTC TGT CCC TAC CAT TGT AG-3′). The PCR mix contains 0.5 µL, 2.5 U Taq DNA polymerase, 0.5 µL of 10 mM d -NTP mix, 2.5 µL of 10 × PCR buffer, 1 µL (0.5 µM) of each primer, 0.75 µL (50 mM) MgCl_2_, 16.75 µL double distilled water and 2 µL DNA (10 ng/µL). PCR products were purified and sequenced at the Centro de Biotecnologia Genomica, Instituto Politecnico Nacional (IPN), Mexico using the Thermofisher Applied Biosystems^®^ 3130 Genetic Analyzer. The 16S rRNA gene sequence was analyzed with Seqman software version 13 and subjected to similarity search using the Blastn program from NCBI (https://blast.ncbi.nlm.nih.gov/Blast.cgi). The sequences were then aligned with homologous sequences retrieved from NCBI database using ClustalW on Mega 6.0 (Tamura et al., 2013), and a phylogenetic tree was constructed using the neighbor-joining algorithm. Reliability of tree topologies was confirmed by bootstrap analysis using 1,000 repeat alignment. *Stenotrophomonas sp.* Pemsol 16S rRNA sequence has been deposited on NCBI with ascension number, KX500117.1.

### Cultivation and growth in PAHs-containing media and bio-emulsification

All PAHs (anthraquinone, 97%; biphenyl, 99%; naphthalene, 99%; phenanthrene, 99%; phenanthridine, 98% and xylene, 98.5%) used for this study were purchased from Sigma Aldrich, Mexico. Growth in PAH tests was carried out using Bushnell Haas (BH) medium with one of the PAH compounds.

A preliminary growth test for *Stenotrophomonas* sp. Pemsol in PAH-containing media showed that it grew at a concentration of 1 mg/ml–5 mg/ml. All the hydrocarbons were dissolved in dimethyl chloride, and the solvent was allowed to evaporate before introducing the hydrocarbons in the experimental system. A 100 µL of the overnight grown culture of bacteria washed in phosphate buffer was inoculated in 100 mL BH medium containing each PAH at a concentration of 1 mg/mL in 250 mL Erlenmeyer flask, which was incubated at 30° C in a rotatory incubator at a revolution of 200 rpm for 8 days. An uninoculated BH medium containing hydrocarbons and a BH medium with *Stenotrophomonas* sp. Pemsol were controls. *Stenotrophomonas’* growth was checked every two days using colony counting. All experiments were in triplicates. Spectrophotometric analysis was also carried out on culture from all experiments to corroborate the observations from the colony counting method. A test with a mix of 5 PAHs (1 mg/ml for each) in the medium was also implemented. Emulsification was tested according to previous reports by Boonchan, Britz & Stanley, 1998 and Sachan & Sachan, 2015, Boonchan, Britz & Stanley, 1998; Panjiar, Sachan & Sachan, 2015. Briefly, 2ml of fresh engine oil were added to 3 ml cell or cell free culture broth in a graduated screw cap test tube. The mix were vortexed vigorously at a high speed for 2 min. Emulsion stability was determined after 2 h, 12 h and 24 h. The emulsification index was calculated by dividing the height of the emulsion layer by total height of the mixture, multiplied by 100. The emulsion activities of Pemsol was compared with other Oil bio-emulsifying *Stenotrophomonas* species isolated in our laboratory.

### Identification of metabolic intermediates from the degradation of naphthalene

Extraction of naphthalene from culture media was performed using an equal volume of hexane in triplicate. Then hexane was eliminated with vacuum pressure for further analysis (FTIR, UPLC-MS, and GC-MS). The metabolite was left to air dry, before doing the FTIR spectrophotometry analysis on them.

#### Fourier-transform infrared spectroscopy (FTIR)

The air-dried samples were analyzed on Bruker Alpha FT-IR spectrometer with Platinum ATR (AXS Inc., Madison, WI, USA) to determine the presence or absence of specific bonds after degradation.

#### Ultra-Performance Liquid Chromatographic-Mass Spectrometry (UPLC-MS) and Gas Chromatography-Mass Spectrometry (GC-MS) analysis

A total of 1 mg of extract was dissolved in one mL in dichloromethane. Then, 0.1 mL of this solution was added to 0.9 mL of methanol, and then analyzed with the Ultra-Performance Liquid Chromatographic (UPLC) with an ACQUITY QDa mass detector from Waters (Milford, MA, USA). The following conditions were applied for the UPLC-MS analysis: column, ACQUITY UPLC^®^BEH C_18_ 1.7 µm 2.1 × 100 mm ; mobile phase A (0.1% formic acid in water), mobile phase B (methanol) and C (Acetonitrile) in a time 0.5-5 min 27%A:25%B; 48 °C; total run time, 5 min; flow rate, 0.3 mL/min; injection volume, 3.0 µL; temperature column, 40 °C.

A total of 1 mg of extract was dissolved in one mL in dichloromethane. Then, 0.1 mL of this solution was added to 0.9 mL of methanol, which was then used for analysis by Gas Chromatographic (7890A GC System) coupled to a Mass detector (5975C inert MSD with Triple-Axis Detector) from Agilent technologies. The following conditions were applied for the GCMS analysis: column, J&W 19091S-433HP-5MS: 30 m ×  250 µm ×0.25 µm; oven program 70 °C for 2 min, then 10 °C/min to 160 °C for 2 min, then 5 °C/min to 240 °C for 2 min, then 30 °C/min to 290 °C for 2 min; Run Time, 34.667 min; injection volume, 2µL.

### Whole-genome sequencing

The genomic DNA was extracted as described above using the Promega DNA extraction kit (USA) according to the manufacturer’s instruction. The extracted bacterial genomic DNA was sequenced at the Unidad Universitaria de Secuenciación Masiva y Bioinformática at the Instituto de Biotecnología, UNAM with the Illumina MiSeq platform.

#### Genome assembly and annotation

The reads quality was checked with Fastqc (Andrews, 2010) and the adaptors from the raw reads were trimmed with trim-galore version 4.10, which also filtered out reads with poor quality. De novo genome assembly was carried out with a standalone Spades 3.11.1 genome assembler (Center for Algorithmic biotechnology, St. Petersburg State University, Russia) (Bankevich et al., 2012). The assembly’s quality was checked with QUAST (Gurevich et al., 2013). The assembled contigs were ordered and reduced into a single scaffold with MedusaCombo, an online genome multi-draft scaffolder (Bosi et al., 2015). The assembled genome was annotated with Prokka annotating pipeline version 1.12 and PGAP (Seemann, 2014; Tatusova et al., 2016). Further functional genome annotation was done with online genome analysis server WebMGA (http://weizhong-lab.ucsd.edu/metagenomic-analysis) (Wu et al., 2011). The KEGG functions and COG categories present in the genome were predicted with the WebMGA online server. The presence of transposon and insertion sequences was predicted with a web-based analysis tool software ISsaga (http://issaga.biotoul.fr/ISsaga2/issaga_index.php) (Varani et al., 2011). The Pan core genome analysis for *Stenotrophomonas* sp. Pemsol and 11 other *Stenotrophomonas* species was performed to determine the unique genes in *Stenotrophomonas* sp. Pemsol. These genomes include *Stenotrophomonas maltophilia* JV3, *Stenotrophomonas maltophilia* ASS1, *Stenotrophomonas pavani* LMG, *Stenotrophomonas rhizophilia* QLP4, *Stenotrophomonas pictorium* JCM 9942, *Stenotrophomonas maltophilia* K279a, *Stenotrophomonas nitrireducen* 2001, *Stenotrophomonas panacihumi*, *Stenotrophomonas maltophilia* ATCC 19687, *Stenotrophomonas maltophilia* R551-3, and *Stenotrophomonas* sp. SKK. The annotation of the unique genes for Pemsol was done with the eggNOG mapper and blast2go (Conesa et al., 2005; Huerta-Cepas et al., 2017). Furthermore, the homologous unique genes in Pemsol’s genome were analyzed by blast search on the NCBI database and the synteny of the genes or gene fragments in the corresponding genomes were compared with the help of SyntTax and RAST annotation server.

#### Prediction of genomic islands (GIs)

The genomic islands (GIs) in Pemsol’s genome were predicted with the genomic IslandViewer 4 (Bertelli et al., 2017).

#### Comparative genome analysis

Genetic relatedness with other *Stenotrophomonas* species was determined by analyzing the Average Nucleotide Identity (ANI) on J speciesWS (Richter et al., 2016) and Genome-Genome distance hybridization (GGDH) (Auch et al., 2010) tools. Further analysis on Pemsol was carried out in the Integrated Microbial Genome (IMG) server (https://img.jgi.doe.gov) and Kbase Platform (https://narrative.kbase.us/narrative/ws.27061.obj.1).

The complete genome sequence of Pemsol has been deposited on DDBJ/EMBL/GenBank under the accession number CP025780.1.

## Results

### Isolation and identification of *Stenotrophomonas* strain Pemsol from crude oil-contaminated soil

The objective of this work is to isolate a *Stenotrophomonas* species that could be used for bioremediation of oil-polluted soil. Thus, StenoVIA agar medium was used for the selection of *Stenotrophomonas* strains (Kerr et al., 1996). Several uniform colonies with characteristic yellow color appeared on the selective medium after 48 h of incubation. The colonies that appeared on plates were characterized biochemically (Table 1) and the 16S rRNA gene fragment was amplified with steno1 and steno2 primers. The amplified fragments were sequenced, and blast search analysis of the sequences confirmed that each clone belongs to the genus *Stenotrophomona*s. The phylogeny produced from the aligned 16S rRNA sequence showed that Pemsol is closely related to *Stenotrophomona*s *maltophilia* M27 (Fig. S1).

**Table 1:** Metabolic characteristics of *Stenotrophomonas* sp Pemsol.

**Growth substrate**	**Response**
Catalase	+ +
Oxidase	−
Galactose	−
Fructose	+ +
Lactose	−
Mannitol	−
Arabinose	−
Maltose	−
Mannose	+ +
Glucose	−
Citrate	−
Aesculin	+ +
Trehalose	+ +
Dulcitol	−
Phenylalanine	−
Serine	−
Lysine	+ +
Starch	−
Tween80	+ +
gelatin	+ +
Hydrogen sulfide	+ +

### Preliminary PAH survival test

The clone Pemsol was chosen for further analysis because of its good resistance to PAH. *Stenotrophomonas* sp. Pemsol ‘s ability to grow and survive in different PAHs was evaluated in BH minimal media with PAHs as a sole carbon source at a concentration ranging from 1 mg/ml to 5 mg/ml. Pemsol showed a good capacity to grow at these concentrations in the 8-day study. *Stenotrophomonas* sp. Pemsol showed better growth rate at a concentration of 1mg/ml in contrast to the growth at higher concentration of PAHs (Dataset 4).

### Utilization of PAHs by *Stenotrophomonas sp.* Pemsol as a sole carbon source

**Figure 1:**
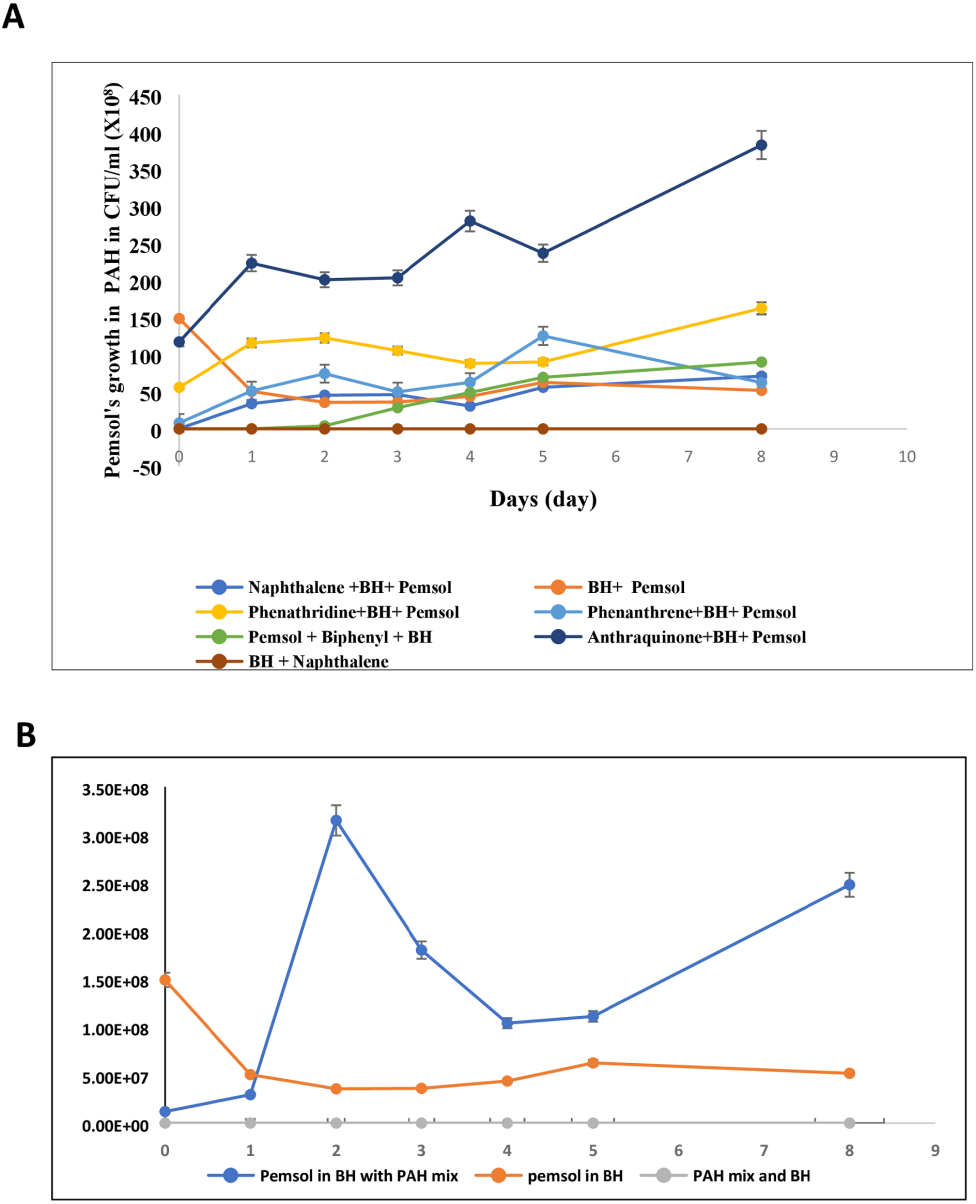
The growth of *Stenotrophomonas* sp. Pemsol using different PAHs as unique carbon source. (A) Pemsol growth in individual PAH; (B) Pemsol’s growth in five PAH mix.

*Stenotrophomonas* sp. Pemsol grew well in BH medium supplemented with biphenyl, phenanthrene, phenanthridine, naphthalene and anthraquinone as sole carbon source at a concentration of 1 mg/mL but did not exhibit growth in BH medium supplemented with xylene, as shown in Fig. 1 (Fig. S2 showed Pemsol’s growth in BH solid medium). Pemsol also displayed the ability to grow in a mix of the five PAHs at the final concentration of 5 mg/mL (1 mg/mL for each) (Fig. 1B). Pemsol showed two growth peaks in this mix, indicating that this strain may prefer to use some compounds as sole carbon source rather than others.

### Bio-emulsion and surfactant production in *Stenotrophomonas sp.* Pemsol

**Figure 2:**
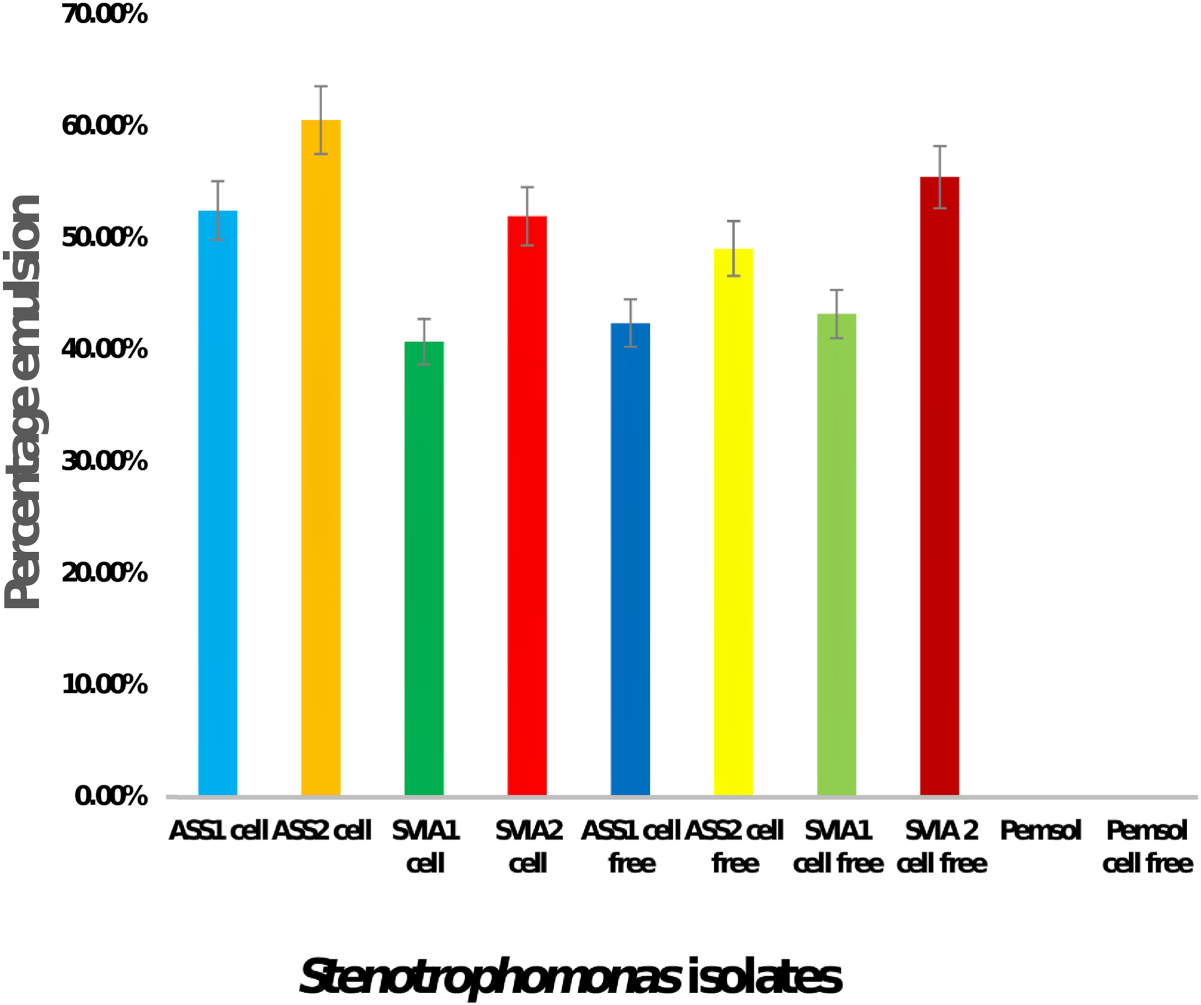
Bio-emulsification activity of *Stenotrophomonas* sp. Pemsol.

Emulsion and surfactant production can assist in bacterial degradation of PAHs. Bio-surfactant production usually enhances the dislodging of PAH attached to surfaces in water, thereby making hydrophobic hydrocarbon available for the use of bacteria (Cameotra & Bollag, 2003). Thus, the emulsion and surfactant production in *Stenotrophomonas* sp. Pemsol was evaluated, as described by Boonchan, Britz & Stanley (1998), Panjiar, Sachan & Sachan (2015), Boonchan, Britz & Stanley (1998), Panjiar, Sachan & Sachan (2015). The result showed that Pemsol cannot produce bio-surfactants as it did not bio-emulsify PAHs (Fig. 2).

### Analysis of degradation products using FTIR spectroscopy

To confirm if the PAH degradation occurred in the minimal medium using PAH as sole carbon source in which Pemsol grew, one of the PAHs (naphthalene) was chosen for the metabolite analysis. The ability of *Stenotrophomonas* sp. Pemsol to degrade naphthalene was analyzed using FTIR spectrometry. New peaks observed at wavelengths -OH (3,200–2,800 cm^−1^), -C = O_(CH2)_ (1,684 cm^−1^), -C = O_(OH)_ (1,641 cm^−1^), -CH _2_(2911)) after 15th day of degradation study and -OH (3,300–3,100 cm^−1^), -C = O (1,690 or 1,700 cm^−1^), -CH_2_ (3,001 cm^−1^) after the 30th day provided evidence of degradation of naphthalene by *Stenotrophomonas* sp. (Figs. S3A–S3C).

### UPLC-MS and gas chromatography-MS analyzes of degradation products

**Figure 3:**
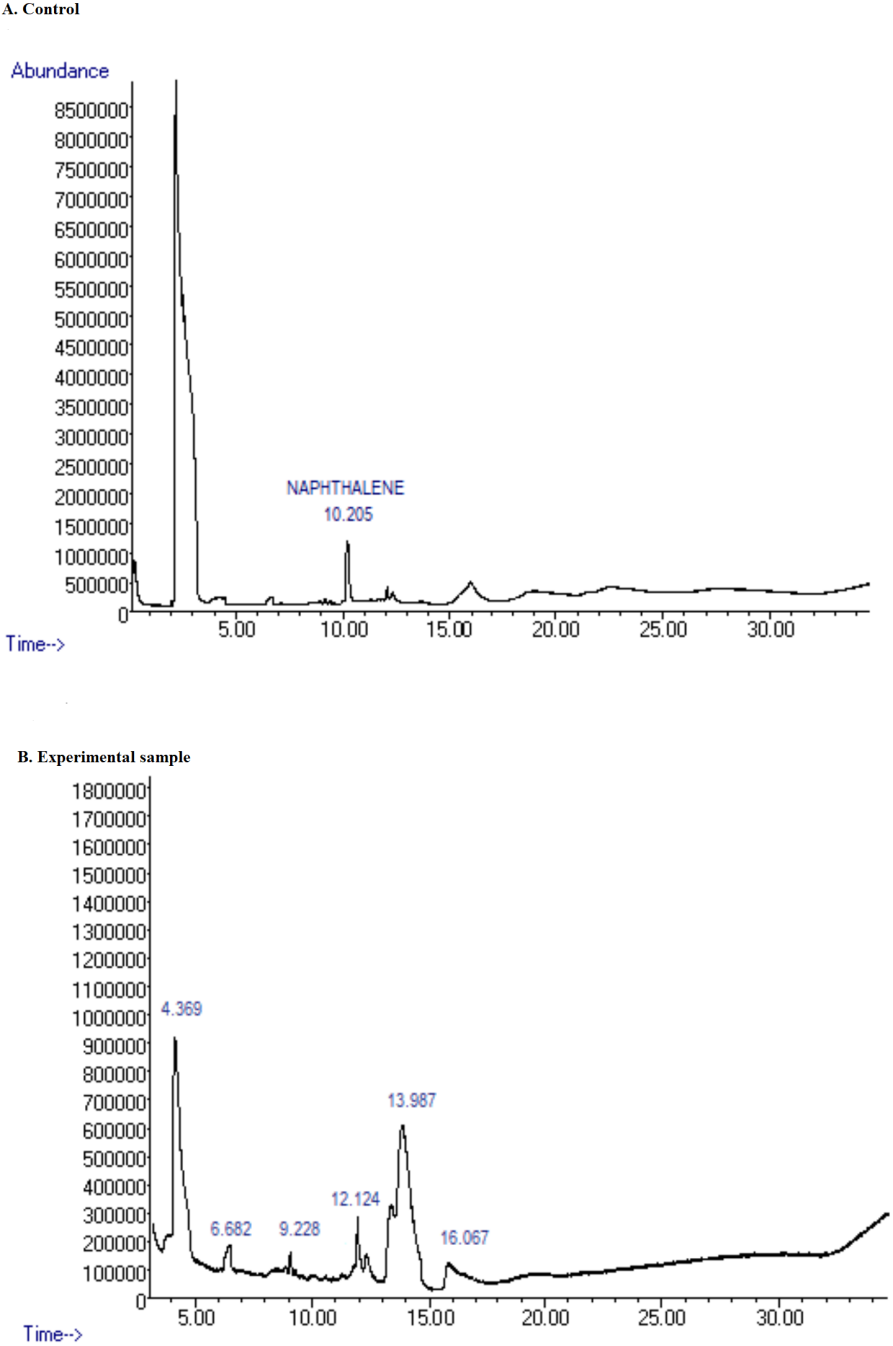
GC-MS image of the metabolites formed by Pemsol degradation of Naphthalene. (A) GC-MS spectrum for Naphthalene; (B) GC-MS spectrum for the metabolite formed from the degradation of Naphthalene by Pemsol.

The UPLC-MS and GC-MS analyses were performed to detect the metabolites formed from the degradation of naphthalene after the 30-day experiment. The absence of a peak corresponding to naphthalene on the spectra obtained from GC-MS analysis confirmed the degradation of naphthalene, comparing with the spectra in the control (Fig. 3). Meanwhile, a peak with a molecular weight of 109.98 was seen as the major metabolites in the UPLC-MS spectra. This peak is estimated to be C_6_H_5_OH corresponding to the molecular weight for catechol (Figs. S4–S5). It could thus be inferred that the degradation of naphthalene by Pemsol involved the formation of catechol.

### Genome analysis of *Stenotrophomonas sp*. Pemsol

**Figure 4:**
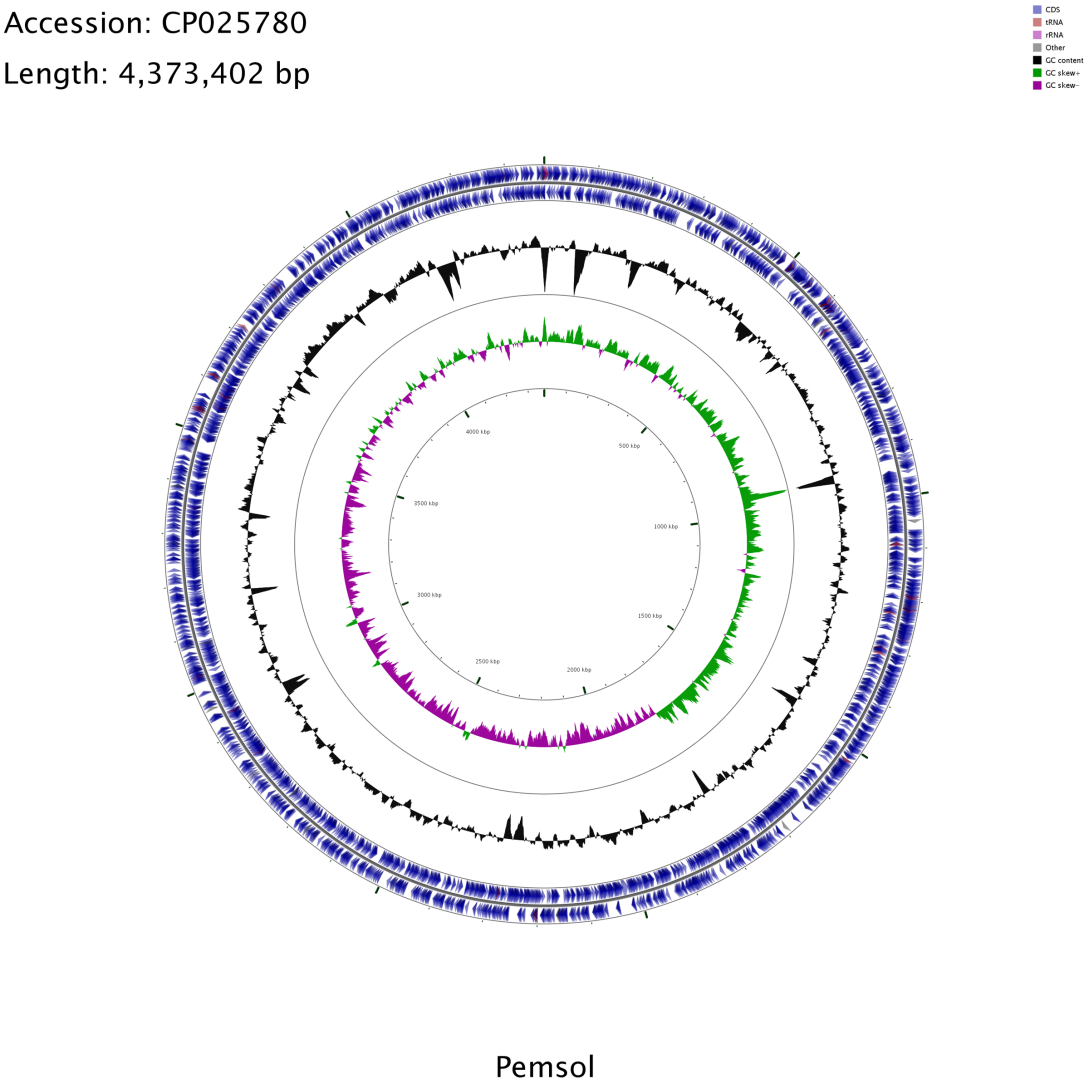
Circular genome map for *Stenotrophomonas* sp. Pemsol.

Several strains belonging to the genus *Stenotrophomonas* have been sequenced, including clinical isolates (Lira et al., 2012; Iyer, Iken & Leon, 2016), but to date, no genome analysis with emphasis on PAH degradation has been reported in this genus. *Stenotrophomonas* sp. Pemsol was sequenced using the Illumina next generation sequencing technology. The completely sequenced Pemsol’s genome was assembled de novo to 62 contigs. These contigs were then reduced to one contig with Medusa Scaffolder. The genome is composed of a single circular chromosome of 4,373,402 bp (Table 2, Fig. 4).

**Table 2:** Genome feature.

Features	Genome
DNA, total number of bases	4,373,402
DNA coding number of bases	4,370,061
DNA G + C content (%)	66.59%.
Misc_RNA	39
Protein coding genes	3,905
rRNA genes	3
tRNA genes	67
tmRNA	1
Genes	4,037

### Genetic basis for the degradation of PAH by *Stenotrophomonas sp*. Pemsol

The annotation and deep sequence analysis of the genome showed that it possesses 145 genes that are associated with PAH degradation (Table S1). In this category are nine genes which code for the enzymes in the lactoylglutathione lyase family COG0346 (PEM_01474; 01733; 01959; 02738; 02855; 02960; 02961; 03007; 03383). The lactoylglutathione lyase family protein usually helps in the degradation of aromatic compound (Mesarch, Nakatsu & Nies, 2000). One of the lactoylglutathione (PEM_03383) was predicted to be a catechol 2, 3 dioxygenases on the JGI-IMG analysis platform (Fig. S6). This gene is essential for the conversion of salicylate aldehyde to catechol in the naphthalene’s metabolic degradation pathway (Grund, Denecke & Eichenlaub, 1992). It also has a gene which codes for salicylate hydroxylase (PEM_02405) (COG0654) (EC:1.14.13.1) (*nahG*). This enzyme catalyzes the removal of the carboxyl group at position 1 of salicylic acid and replaces the carboxyl with a hydroxyl group in the same position. This substitution converts salicylic acid to catechol (Goyal & Zylstra, 1997; Bosch et al., 1999). Two genes, homogentisate 1,2-dioxygenase (PEM_03309) that are involved in the catabolism of aromatic rings (Borowski, Georgiev & Siegbahn, 2005), and 2, 4 dihydroxyacetophenonedioxygenase (PEM_00137), which helps in the cleavage of carbon–carbon bond in a substituent aromatic ring, were also detected (Keegan et al., 2014). The information mentioned above ratifies the capacity of *Stenotrophomonas* sp. Pemsol to degrade naphthalene as shown in the experiments.

There are several other genes in *Stenotrophomonas* sp. Pemsol which can enhance the degradation of PAHs. One of such genes is chloromuconate isomerase (PEM_00043, EC:5.5.1.7), which catalyzes the degradation of 1,4-dichlorobenzene degradation (Schmidt, Remberg & Knackmuss, 1980). Others include carboxymethylenebutenolidase which catalyzes the conversion of methyl catechol to 4-carboxymethyl-4-methylbut-2-en-4-olide during the degradation of toluene to 4-oxohex-2-enedioate in *Burkholderia* (Dobslaw & Engesser, 2015), 4-oxalocrotonate tautomerase (PEM_00595) known to be associated with the degradation of toluene, o-xylene, 3-ethyltoluene, and 1,2,4-trimethylbenzene (Chen et al., 1992). Also present in the genome is biphenyl-2,3-diol 1,2-dioxygenase (PEM_03239, EC:1.13.11.39) that is associated with the degradation of biphenyl and gamma-hexachlorocyclohexane (Yam et al., 2009). Several monooxygenases and dehydrogenases are present in the Pemsol, which could catalyze the degradation of aromatic hydrocarbon and other xenobiotics (Versalovic et al., 2016; Pal et al., 2017) (Table S1).

### Specific PAH degrading genes in genomic islands (GIs)

**Figure 5:**
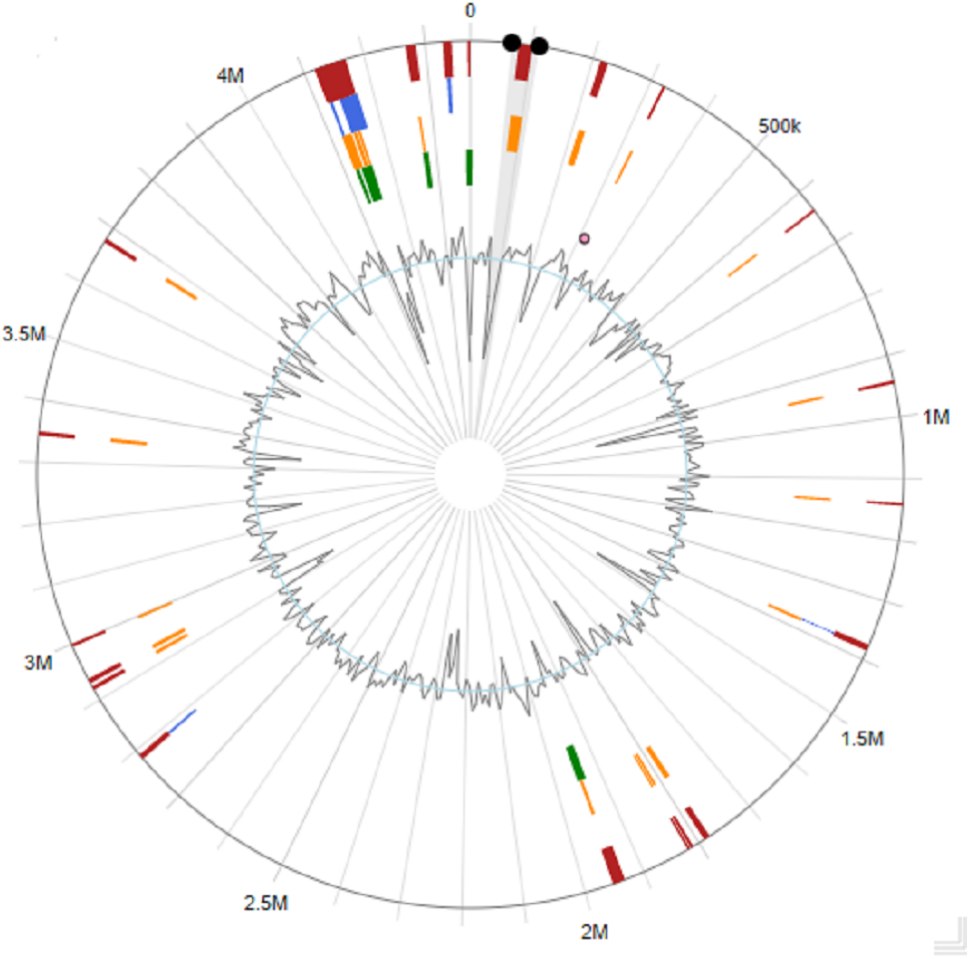
Genomic Island distribution in *Stenotrophomonas* sp. Pemsol.

Microbes have been widely known to acquire new properties via horizontal gene transfer. In Pemsol, 29 genomic islands (GIs) were identified (Fig. 5, Table S2), which is 336,552 bp in length and constituting 7.7% of the genome. Table S3 contains the annotation of the genes in the GIs for *Stenotrophomonas* sp. Pemsol. Some genes on the GIs showed similarity with genes found in bacteria of other taxa. Most genes were predicted to be of unknown function. Twelve genes encoding transporters and some transcriptional regulators were found in the GIs. For example, a regulatory protein (PEM_01297) required for the regulation of xenobiotics’ degradation is present in the GI (1932729–1952883). Similarly, a Cysteine-liking transporter (PEM_00076) (GI 1816906–1820994) and another sulfite transporter (PEM_03784) (GI, 4135477–4158355) needed for the transport of sulfite molecules were also found in these regions (Takumi & Nonaka, 2016).

### Analysis of unique genes in adaptation for survival in PAH environment

The Pan-Core genome analysis was done for *Stenotrophomonas* sp. Pemsol and 11 other *Stenotrophomonas* species. The result of the analysis showed that *Stenotrophomonas* sp. Pemsol possesses 154 unique genes. Most genes identified to be unique in Pemsol are present in the genomic islands. The predicted functions for these genes are shown in Table S4. Some of these genes are involved in the degradation of PAH.

### A comparative COG category analysis for *Stenotrophomonas* sp. *Pemsol*

The COG categories in *Stenotrophomonas* species was compared in Pemsol and other *Stenotrophomonas* strains. *Stenotrophomonas* sp. Pemsol has a higher number of genes in some COG categories than the other 11 *Stenotrophomonas* species examined. The Fischer test statistical analysis showed that *Stenotrophomonas* species Pemsol has more genes in the categories: energy production and conversion (C) (6.01%), amino acid transport and metabolism coenzyme transport and metabolism (H) (6.78%), cell motility (N) (3.74%), secondary metabolite biosynthesis, transport and metabolism (Q) (2.58%), general function prediction (R) (8.4%), function unknown (S) (6.4%), signal transduction (T) (6.52%), defense mechanism (V) (3.19%), extracellular mechanism (W) (1.77%) (Table S4).

**Figure 6:**
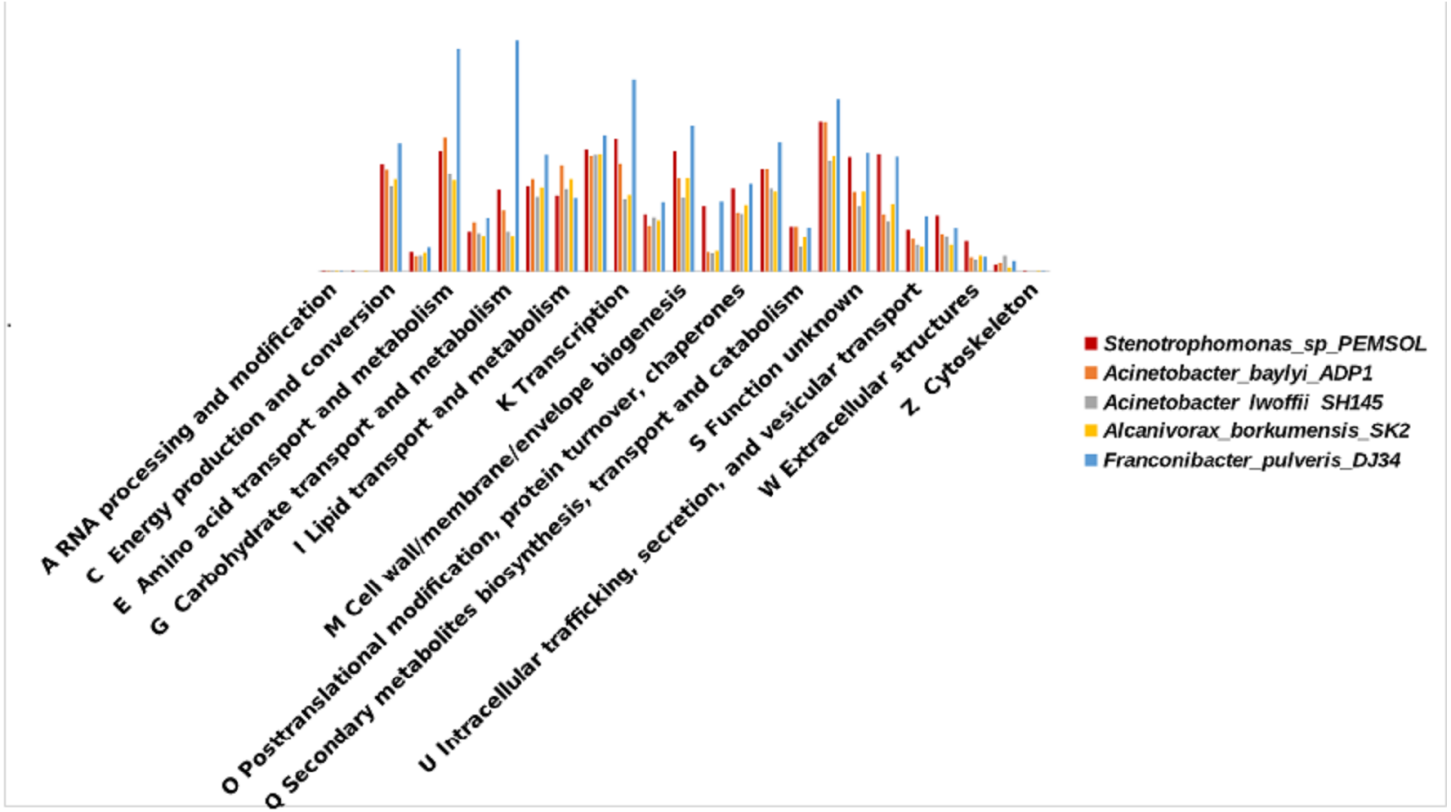
COG distribution comparison of Pemsol and other hydrocarbon degrading bacteria.

The COG categories in Pemsol was compared with the COGs in four other PAH-degrading bacteria (*Acinetobacter_baylyi* _ADP1, *Acinetobacter_lwoffii* _SH145, *Alcanivorax_borkumensis* _SK2, *Franconibacter_pulveris* _DJ34) previously reported. The result revealed that Pemsol has more genes in the COG category G, N, T, and W than the other hydrocarbon-degrading bacteria (Fig. 6, Table S5). These categories are associated with carbohydrate transport and metabolism (4.54%), cell movement (3.74%), signal transduction mechanisms (6.52%), and extracellular structure (1.75%).

### Pemsol’s genomic relatedness with other *Stenotrophomonas* strains (ANI and GGDH)

**Figure 7:**
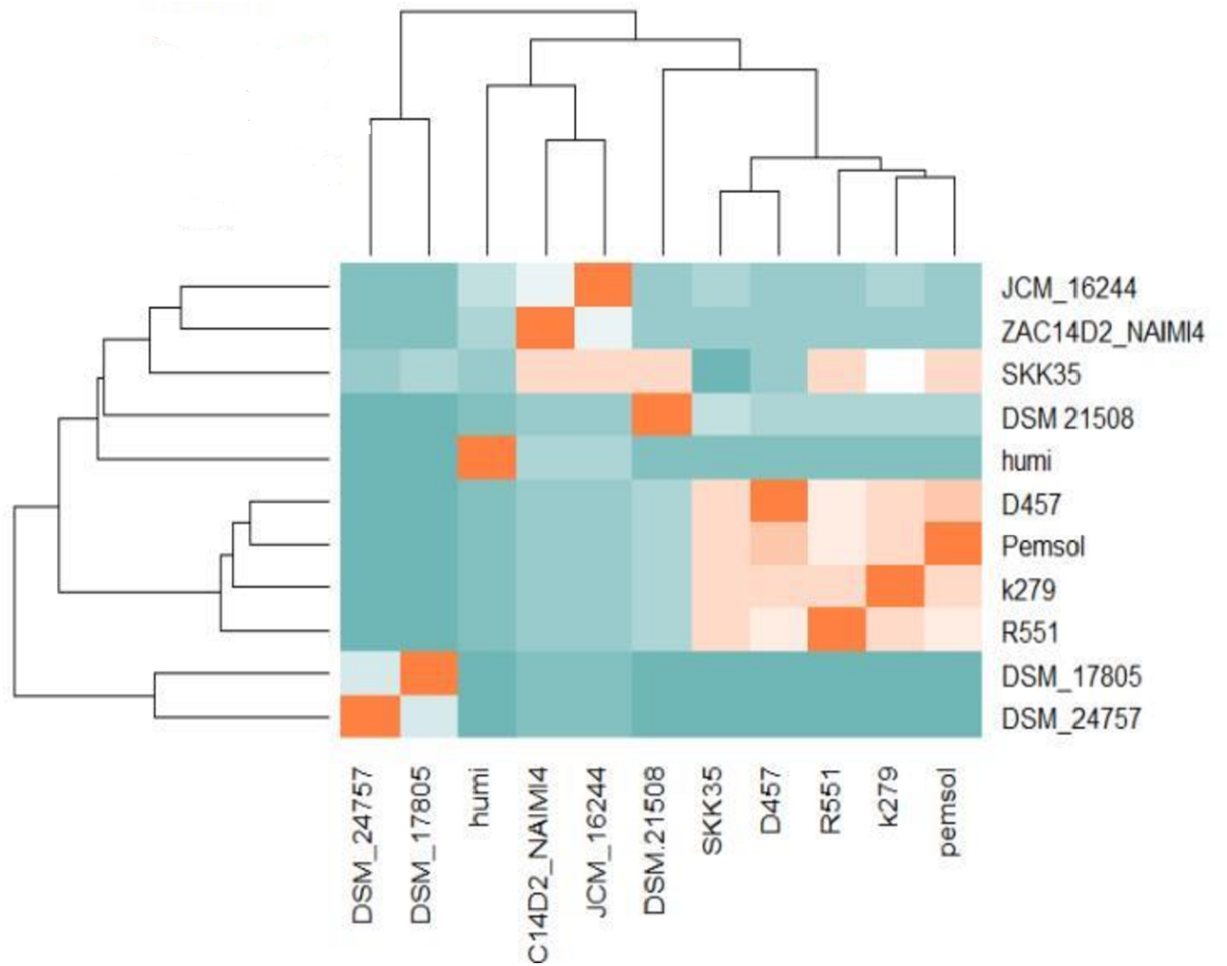
Average nucleotide identity score for Pemsol and other *Stenotrophomonas* species.

Average Nucleotide Identity blast (ANIb) was employed for the identification of *Stenotrophomonas* sp. Pemsol to the species level. The ANIb showed that *Stenotrophomonas* sp is likely a new species as it shares the ANIb similarity closest to *S. maltophilia* K279a at the level of 91.2% (Fig. 7) and the GGDH analysis result gave the Digital DNA Hybridization (DDH) score at the level of 44.2% using the recommended formula 2 (Table S6).

## Discussion

The SVIA selective medium used in this study successfully select *Stenotrophomonas* strains (Kerr et al., 1996). The biochemical characteristics of the colonies which appear on the SVIA conformed with the features described for members of the genus *Stenotrophomonas* (Denton & Kerr, 1998; Ryan et al., 2009; Brooke, 2012)*. Stenotrophomonas* sp. Pemsol was obtained by this method from the oil-contaminated site. The sequences of 16S rRNA fragments of bacteria is known to provide valid information about their classification. *Stenotrophomonas* sp. Pemsol showed 99% similarity with *Stenotrophomonas maltophilia* M27 (Fig. S1). It could be inferred that *Stenotrophomonas* sp. Pemsol is likely a *S. maltophilia* strain. The ANI analysis result, however, showed that Pemsol only shares 91.2% ANI score with *S. maltophilia* K279a as the closest strain (complete genome sequence is not available for the strain M27). Thus, *Stenotrophomonas* sp. Pemsol could be a novel species (Figueras et al., 2014) (Fig. 7). Similarly, the DDH (44.2%) value between Pemsol and K279a is below the threshold for the same species (Table S6). Although the 16S RNA gene identity showed a high level of relatedness with *S. maltophilia* (Fig. S1), the ANIb similarity score and the DDH score suggest Pemsol could be a new species.

*Stenotrophomonas* are an essential group of bacteria with an enormous capacity to use a wide range of substances for their growth. *Stenotrophomonas*’ ability to survive in different environments, including those with limited nutrients, has been reported in many studies (Ryan et al., 2009). The strains with such capabilities have been isolated from different environments ranging from common environment to extreme environment such as highly acidic or alkaline environments (Boonchan, Britz & Stanley, 1998; Juhasz, Stanley & Britz, 2000; Samanta, Singh & Jain, 2002; Gao et al., 2013; Tebyanian, Hassanshahian & Kariminik, 2013; Arulazhagan et al., 2017). Thus, the recovery of Pemsol from crude oil-contaminated soil further confirmed the versatility *Stenotrophomonas*’ versatility (Ryan et al., 2009).

Previous studies have shown that some *Stenotrophomonas* strains can grow on and degrade xenobiotics such as PAHs and organophosphates (Ryan et al., 2009; Iyer, Iken & Leon, 2016). *Stenotrophomonas* sp. Pemsol grew effectively in BH medium with different PAHs as the sole carbon source except xylene, implying that Pemsol could possibly degrade these PAHs, making it a potential strain for the remediation of crude oil-contaminated regions.

**Figure 8:**
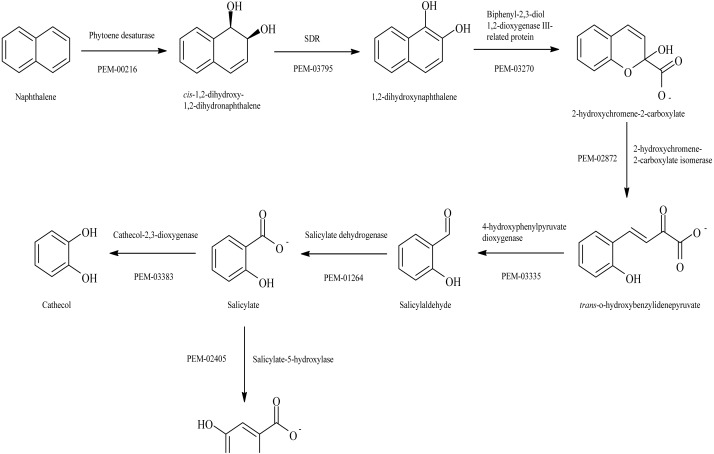
Proposed degradation Pathway for Naphthalene degradation by Pemsol.

Pemsol grew in the tested PAHs at the concentration ranging from 1–5 mg/ml. Although it can grow in higher concentrations, Pemsol displayed better growth at a concentration of 1 mg/ml. Previous studies have reported the growth of *Stenotrophomonas* species in other PAHs (Smith, 1990; Johnsen, Wick & Harms, 2005). Juhasz, Stanley & Britz (2000) demonstrated *Stenotrophomonas maltophilia strain VUN 10,003*’s growth in an experiment trying to degrade and detoxify pyrene, fluoranthene, benz[a]anthracene, benzo[a]pyrene, dibenz[a,h]anthracene and coronene in a 63-day experiment (Juhasz, Stanley & Britz, 2000). In our study, the analysis of the degraded products by GC-MS and UPLC-MS showed that Pemsol completely degraded naphthalene in the minimal medium in a system wrapped with a foil for 30 days. Catechol is the major constituent formed. Catechol is an intermediate product formed from the degradation of PAHs by bacteria before they are mineralized (Gibson, Koch & Kallio, 1968). According to the analysis of the annotated enzymes in Pemsol’s genome, we proposed a naphthalene degradation pathway for Pemsol shown in Fig. 8, which is similar to the pathway described by Eaton & Chapman (1992). The degradation of other PAH compounds was not performed by metabolite analysis, however, the use of other PAH compounds as sole carbon sources for Pemsol’s growth was confirmed, indicating that Pemsol possesses the capacity to degrade these PAHs, although it may employ different metabolic pathways for their degradation. Thus, *Stenotrophomonas* sp. Pemsol is an important species with the capacity to degrade and metabolize PAHs.

Bio-surfactants production in some bacteria is known to enhance PAH degradation (Willumsen & Karlson, 2004; Ruggeri et al., 2009; Das & Chandran, 2011). Bio-surfactant production by bacteria usually ensures the solubilization of hydrophobic PAH. Once hydrophobic PAHs become soluble, bio-degrading bacteria have easier access to use them (Willumsen & Karlson, 2004). Das et al. (2015) reported the presence of 25 genes related to biosurfactant production in a PAH-degrading *Pseudomonas aeruginosa* N002. However, several bacteria, including *Stenotrophomonas,* which can degrade PAH without biosurfactant production, have been reported (Bello-Akinosho et al., 2016; Arulazhagan et al., 2017). Pemsol does not produce surfactant, thus it is important to understand the molecular basis for this characteristic. The analysis of Pemsol’s genome showed that it possesses only three genes, phosphomannomutase (PEM_00211) and two glycosyls 2 family transferase genes (PEM_00203, 00204) that can participate in bio-surfactant formation (Pal et al., 2017). Particularly, it lacked some basic genes (such as rhlA, B, R and I which encode rhamnosyltransferase) required for bio-surfactant production in bacteria, 1 (Satpute et al., 2010). Although Pemsol did not bioemuslfiy PAHs, it effectively degraded the PAHs for growth. It can thus be deduced that surfactant production is not essentially required for the uptake of PAHs.

**Figure 9:**
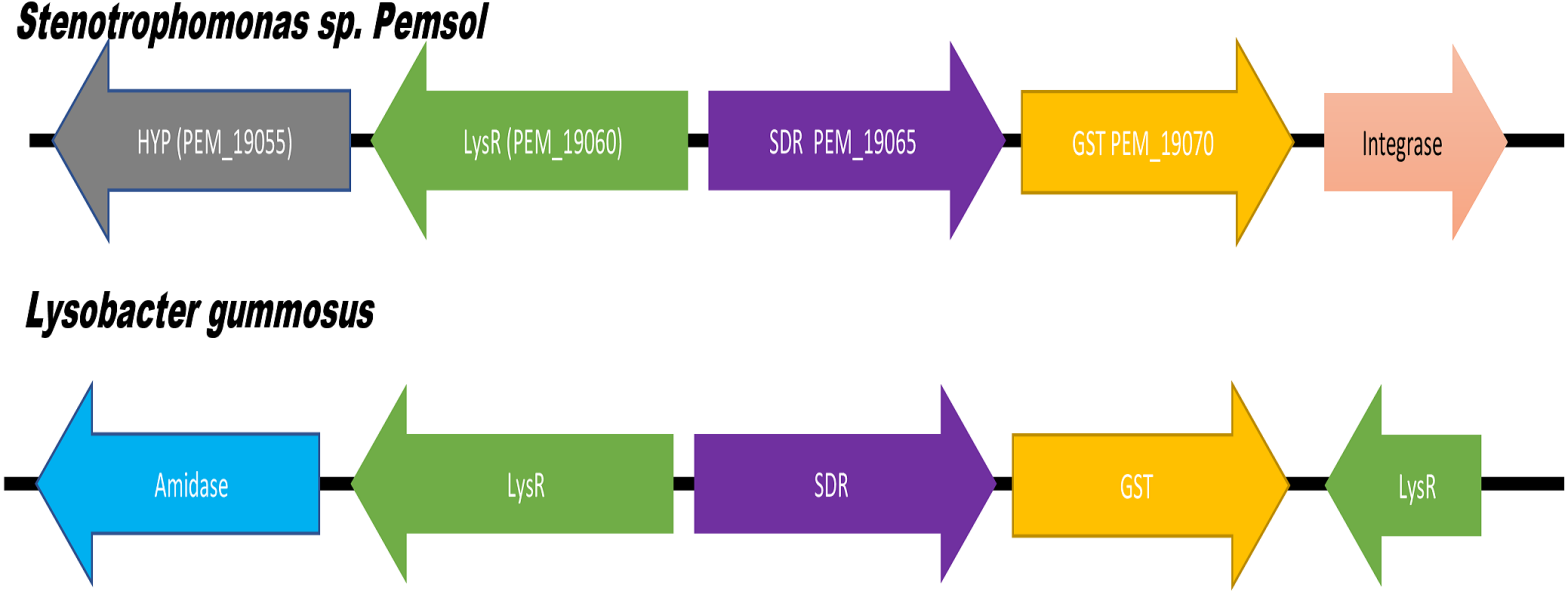
Identified unique gene region that is associated with the degradation of PAH.

The analysis of Pemsol’s genome with the Island viewer 4 showed that it possesses 29 GIs and several genes or gene clusters in GIs participate in the degradation of PAHs. For example, a gene cluster contains 3 genes which encode the proteins short dehydrogenase reductase (SDR), LysR, and glutathione S-transferases (GST). SDR can catalyze the reduction of C=C bond between an aromatic compound (Kavanagh et al., 2008). The KEGG database clearly showed that GST is directly involved in the degradation of many hydrocarbon compounds (benzopyrene, naphthalene, trichloroethylene, bromobenzene, etc.). The LysR-type transcriptional regulator (LTTR) has been reported to have a significant function in regulating genes that are important for the catabolism of aromatic compound, cell motility, and quorum sensing (Pal et al., 2017). Thus, LysR gene in this gene cluster could be involved in the regulation of SDR and GST for PAH degradation. It is interesting that the closest orthologues of the 3 proteins SDR, LysR, and GST are in the same gene order with the identity of 91.6%, 94.24 and 85.65% respectively in a distant species *Lysobacter gummosus* on NCBI database, implying that this gene cluster in these two species has a common origin and Pemsol could have obtained this gene cluster by horizontal gene transfer (Fig. 9).

## Conclusion

*Stenotrophomonas* sp. Pemsol was isolated from crude oil-contaminated soil from Tabasco, Mexico. It grew in the presence of five PAHs (biphenyl, anthraquinone, phenanthrene, naphthalene, and phenanthridine) as unique carbon source except xylene. The identification of Pemsol confirmed that it is a member of the genus *Stenotrophomonas*. The ability of Pemsol to degrade PAH was confirmed by its activities on naphthalene as revealed by FTIR, UPLC-MS and GC-MS analysis. The complete genome of Pemsol was sequenced and the analysis revealed that it possesses 145 genes that are involved in the degradation of PAHs but only three genes associated with bio-emulsification, leading to no biosurfactant production. The presence of some genes associated with the degradation of PAHs in the genomic islands inferred that those genes were horizontally acquired. Compared with other four sequenced hydrocarbon-degrading bacteria, Pemsol is much richer in genes for the COG category G, N, T, and W, which are mainly relevant to hydrocarbon utilization and interaction with the environment. These results give insight into the genetic basis involved in the survival of Pemsol in its oil-contaminated site and provide a guide on the possible strategies for the bioremediation of an oil-polluted environment with *Stenotrophomonas* sp*.* Pemsol without biosurfactant production.

##  Supplemental Information

10.7717/peerj.8102/supp-1 Supplemental Information 1

10.7717/peerj.8102/supp-2 Supplemental Information 2 This table contains the growth of Pemsol and other hydrocarbon degrading bacteria tested for their tolerance and growth in PAH in a 20 day tolerance study prior to degradation analysis. It also contain the table for the emulsion test of Pemsol as compared EACH TABLE represents Stenotrophomonas species’ growth in different PAH

10.7717/peerj.8102/supp-3 Supplemental Information 3 Table for the COGs category comparison betwen Pemsol and selected member of other Stenotrophomonas species COGs category comparison betwen Pemsol and selected member of other Stenotrophomonas species.

10.7717/peerj.8102/supp-4 Supplemental Information 4 Table of COGs comparison statistic between Stenotrophomonas sp. Pemsol and five other PAH degrading bacteria COGs category comparison betwen Pemsol and and five other PAH degrading bacteria

10.7717/peerj.8102/supp-5 Supplemental Information 5 raw table for figure 2 The table contains the growth of Stenotrophomonas sp. Pemsol in different PAH studies in the 8 days of tolerance studies

10.7717/peerj.8102/supp-6 Supplemental Information 6 Tables for the genes involved in the degradation and survival of Pemsol The tables contain the genes associated with PAH degradation by pemsol and their function, the genes that are on the genomic island, and the genes that are unique to Pemsol

10.7717/peerj.8102/supp-7 Supplemental Information 7 Figures showing the spectra from FTIR,and UPLC MS analysis for the product formed from the degradation of naphthalene by Pemsol Fig. 1 Phylogenetic tree of* Stenotrophomonas sp*. Pemsol with other members of the genus * Stenotrophomonas* based on the sequence of 16S rRNA gene, Fig. 2**:**Pemsols growth in solid media with PAH as sole carbon source Fig. 3 contains FTIR spectrum while Figs. 3 and 4 contain UPLC MS spectra, Fig. 6 Catechol 2, 3 dioxygenase containing region in Pemsol

## Additional Information and Declarations

### DNA Deposition

The following information was supplied regarding the deposition of DNA sequences: The raw data is available at GenBank: CP025780.1.

### Data Availability

The following information was supplied regarding data availability: The data is available at NCBI: NZ_CP025780.1.
