# Supplementary material for: The Polycyclic Aromatic Hydrocarbon (PAH) degradation activities and genome analysis of a novel strain Stenotrophomonas sp. Pemsol isolated from Mexico
Source: PeerJ. 2020 Jan 6;8:e8102. doi: 10.7717/peerj.8102 (PMC6951288; doi:10.7717/peerj.8102)
Supplement: Supplemental Information 1 [file peerj-08-8102-s001.pdf]

Instituto Nacional de Investigaciones Forestales, Agrícolas y Pecuarias

CNRG Centro Nacional de Recursos Genéticos

CM-CNRG INIFAP

La Colección de Microorganismos del Centro Nacional de Recursos Genéticos que pertenece al Instituto Nacional de Investigaciones Forestales, Agrícolas y Pecuarias con número de registro ante la WFCC 1006 (CM-CNRG) expide el siguiente:

CERTIFICADO DE DEPÓSITO

Correspondiente a la cepa microbiana

Stenotrophomonas* sp. Pemsol *con número de registro CM-CNRG 697

Depositada por el **Dr. Temidayo Oluyomi Elufisan, M. en E. Isabel Cristina Rodríguez Luna, M en C. Alejandro Sánchez Varela y Dr. Xianwu Guo** con domicilio en: **Centro de Biotecnología Genómica, Instituto Politécnico Nacional, Boulevard del Maestro con Elías Pina, Col. Narciso Mendoza, C.P. 88710, Reynosa Tamaulipas.**

Bajo la modalidad de **DEPÓSITO PÚBLICO** conforme a los criterios de viabilidad, pureza y estabilidad genética establecidos por la World Federation of Culture Collection, y conforme a lo declarado por el depositante en el Formulario de registro. Se expide el presente certificado para los fines que al interesado convengan.

**Ingreso a la CM-CNRG**	01/11/2019
**Número de certificado**	CM-CNRG 0697

Tepatitlán de Morelos Jalisco a 10 de diciembre de 2019

CERTIFICA

Dr. Ramón Ignacio Arteaga Garibay
Curador de la CM-CNRG

CM-CNRG INIFAP
AUTORIDAD INTERNACIONAL DE DEPÓSITO
NOTIFICACIÓN 3[illegible]

Autoridad Internacional de Depósito CM-CNRG INIFAP

Centro Nacional de Recursos Genéticos. Blvd. de la Biodiversidad n.° 400
Tepatitlán de Morelos, Jalisco, México. C.P. 47600

Tel.: (55) 3781 8700 ext. 84818, e-mail: cm.cnrg@inifap.gob.mx
