# Supplementary material for: The Polycyclic Aromatic Hydrocarbon (PAH) degradation activities and genome analysis of a novel strain Stenotrophomonas sp. Pemsol isolated from Mexico
Source: PeerJ. 2020 Jan 6;8:e8102. doi: 10.7717/peerj.8102 (PMC6951288; doi:10.7717/peerj.8102)
Supplement: Supplemental Information 6 — The tables contain the genes associated with PAH degradation by pemsol and their function, the genes that are on the genomic island, and the genes that are unique to Pemsol [file peerj-08-8102-s006.docx]

**Supplementary Table 1-6**

| **Table S1: Identified genes in *Stenotrophomonas* sp. Pemsol that are associated with the degradation of naphthalene and polycyclic aromatic hydrocarbon** | | | | | | | |
| --- | --- | --- | --- | --- | --- | --- | --- |
| Query_ID | Hit_ID | KEGG_no | KEGG_description | ko_no | EC_no | COG_no | Associated degradation pathways |
| PEM_00037 | aci: ACIAD3598 | K00120 | glucose-fructose oxidoreductase | ko00625 | N/A | COG1979; COG2141 | Bisphenol degradation, Polycyclic aromatic hydrocarbon degradation, Chloroalkane and chloroalkene degradation, Naphthalene degradation, Aminobenzoate degradation, Limonene and pinene degradation |
| PEM_00037 | nph:NP0254A | K00100 | 1-deoxy-D-xylulose-5-phosphate reductoisomerase | ko00625 | N/A | COG0111; COG0677;COG0743;COG1063 | Fructose and mannose metabolism, Bisphenol degradation, Linoleic acid metabolism, Chloroalkane and chloroalkene degradation, Butanoate metabolism |
| PEM_00043 | sjp: SJA_C1-21130 | K01860 | chloromuconate cycloisomerase | ko00623 | EC:5.5.1.7 | N/A | Chlorocyclohexane and chlorobenzene degradation, Benzoate degradation, Fluorobenzoate degradation, Toluene degradation, Chlorocyclohexane and chlorobenzene degradation, Fluorobenzoate degradation, Toluene degradation |
| PEM_00054 | bpm: BURPS1710b_A2071 | K01041 | glutaconate CoA-transferase, subunit B | ko00626 | N/A | N/A | Geraniol degradation, Naphthalene degradation |
| PEM_00118 | bbt: BBta_6889 | K00799 | glutathione S-transferase | ko00980 | EC:2.5.1.18 | COG0625 | Glutathione metabolism and Metabolism of xenobiotics by cytochrome P459 |
| PEM_00121 | bba: Bd3653 | K01077 | alkaline phosphatase | ko00627 | EC:3.1.3.1 | COG1785 |  |
| PEM_00137 | bra: BRADO5603 | K05915 | 2,4'-dihydroxyacetophenone dioxygenase | ko00363 | N/A | N/A | Bisphenol and Naphthalene degradation |
| PEM_00164 | gbe: GbCGDNIH1_1792 | K00599 | trans-aconitate 2-methyltransferase | ko00624 | N/A | COG0500 | Histidine metabolism, Tyrosine metabolism, Selenoamino acid metabolism, Polycyclic aromatic hydrocarbon degradation |
| PEM_00185 | sfu: Sfum_0938 | K01826 | 5-carboxymethyl-2-hydroxymuconate isomerase | ko00362 | EC:5.3.3.10 | COG3232 |  |
| PEM_00297 | mbb: BCG_0144 | K00680 | phospholipid:diacylglycerol acyltransferase | ko00626 | N/A | N/A | Naphthalene degradation, Aminobenzoate degradation, Ethylbenzene degradation, Limonene and pinene degradation |
| PEM_00342 | xac: XAC0815 | K00599 | trans-aconitate 2-methyltransferase | ko00624 | N/A | COG0500 | Histidine metabolism, Tyrosine metabolism, Selenoamino acid metabolism, Polycyclic aromatic hydrocarbon degradation |
| PEM_00487 | smt : Smal_0786 | K01426 | amidase | ko00627 | EC:3.5.1.4 | COG0154 |  |
| PEM_00496 | sml: Smlt0950 | K00799 | glutathione S-transferase | ko00980 | EC:2.5.1.18 | COG0625 | Glutathione metabolism and Metabolism of xenobiotics by cytochrome P454 |
| PEM_00500 | reh: H16_B1699 | K00001 | alcohol dehydrogenase | ko00625 | EC:1.1.1.1 | COG0604;COG1062;COG1064;COG1454 | Glycolysis / Gluconeogenesis, Fatty acid metabolism, Tyrosine metabolism, Chloroalkane and chloroalkene degradation, Naphthalene degradation, Methane metabolism, |
| PEM_00527 | mth: MTH234 | K01607 | 4-carboxymuconolactone decarboxylase | ko00362 | EC:4.1.1.44 | COG0599 |  |
| PEM_00595 | bte: BTH_II0588 | K01821 | 4-oxalocrotonate tautomerase | ko00362 | EC:5.3.2.- | COG1942 |  |
| PEM_00605 | rlg: Rleg_4614 | K00799 | glutathione S-transferase | ko00980 | EC:2.5.1.18 | COG0625 | Glutathione metabolism and Metabolism of xenobiotics by cytochrome P461 |
| PEM_00622 | bbt: BBta_4719 | K00100 | 1-deoxy-D-xylulose-5-phosphate reductoisomerase | ko00625 | N/A | COG0111;COG0677;COG0743;COG1063 | Fructose and mannose metabolism, Bisphenol degradation, Linoleic acid metabolism, Chloroalkane and chloroalkene degradation, Butanoate metabolism |
| PEM_00632 | bpd: BURPS668_A2562 | K01066 | esterase / lipase | ko00363 | EC:3.1.1.- | COG0657 | Bisphenol degradation Aminobenzoate degradation Tropane, piperidine and pyridine alkaloid biosynthesis |
| PEM_00680 | bme: BMEI1067 | K00680 | phospholipid:diacylglycerol acyltransferase | ko00626 | N/A | N/A | Naphthalene degradation, Aminobenzoate degradation, Ethylbenzene degradation, Limonene and pinene degradation |
| PEM_00701 | reh: H16_B1475 | K01066 | esterase / lipase | ko00363 | EC:3.1.1.- | COG0657 | Bisphenol degradation Aminobenzoate degradation Tropane, piperidine and pyridine alkaloid biosynthesis |
| PEM_00701 | aba: Acid345_2787 | K01061 | carboxymethylenebutenolidase | ko00623 | EC:3.1.1.45 | COG0412 | Chlorocyclohexane and chlorobenzene degradation, Fluorobenzoate degradation, Toluene degradation, Peptidoglycan biosynthesis |
| PEM_00722 | bme: BMEI1388 | K00100 | 1-deoxy-D-xylulose-5-phosphate reductoisomerase | ko00625 | N/A | COG0111;COG0677;COG0743;COG1063 | Fructose and mannose metabolism, Bisphenol degradation, Linoleic acid metabolism, Chloroalkane and chloroalkene degradation, Butanoate metabolism |
| PEM_00760 | sds: SDEG_0903 | K00680 | phospholipid:diacylglycerol acyltransferase | ko00626 | N/A | N/A | Naphthalene degradation, Aminobenzoate degradation, Ethylbenzene degradation, Limonene and pinene degradation, Tyrosine metabolism |
| PEM_00906 | ajs: Ajs_0052 | K00492 | nitric-oxide synthase, bacterial | ko00363 | N/A | COG0654 | Naphthalene degradation Aminobenzoate degradation and Limonene and pinene degradation |
| PEM_01004 | sus: Acid_6224 | K01692 | enoyl-CoA hydratase | ko00362 | EC:4.2.1.17 | COG1024 |  |
| PEM_01018 | pfl: PFL_2342 | K00128 | aldehyde dehydrogenase (NAD+) | ko00625 | EC:1.2.1.3 | COG1012 | Glycolysis / Gluconeogenesis, Pentose and glucuronate interconversions, Ascorbate and aldarate metabolism, Fatty acid metabolism, Valine, leucine and isoleucine degradation, Lysine degradation, Arginine and proline metabolism, Histidine metabolism, Tryptophan metabolism, beta-Alanine metabolism,Glycerolipid metabolism, Pyruvate metabolism, Chloroalkane and chloroalkene degradation, Propanoate metabolism, Limonene and pinene degradation |
| PEM_01045 | buj: BurJV3_1348 | K00799 | glutathione S-transferase | ko00980 | EC:2.5.1.18 | COG0625 | Glutathione metabolism and Metabolism of xenobiotics by cytochrome P457 |
| PEM_01059 | lsa: LSA1776 | K01607 | 4-carboxymuconolactone decarboxylase | ko00362 | EC:4.1.1.44 | COG0599 |  |
| PEM_01062 | bbt: BBta_3786 | K00100 | 1-deoxy-D-xylulose-5-phosphate reductoisomerase | ko00625 | N/A | COG0111;COG0677;COG0743;COG1063 | Fructose and mannose metabolism, Bisphenol degradation, Linoleic acid metabolism, Chloroalkane and chloroalkene degradation, Butanoate metabolism |
| PEM_01125 | smt: Smal_1438 | K00494 | alkanal monooxygenase (FMN-linked) | N/A | EC:1.14.14.3 | N/A |  |
| PEM_01174 | sml: Smlt1754 | K01113 | alkaline phosphatase D | ko00627 | EC:3.1.3.1 | COG3540 |  |
| PEM_01219 | buj: BurJV3_1582 | K00241 | succinate dehydrogenase cytochrome b-556 subunit | ko00623 | EC:1.3.99.1 | COG2009 | Oxidative phosphorylation, Toluene degradation, Butanoate metabolism, Reductive carboxylate cycle (CO2 fixation), Two-component system |
| PEM_01220 | buj: BurJV3_1583 | K00242 | succinate dehydrogenase hydrophobic membrane anchor protein | ko00623 | EC:1.3.99.1 | COG2142 | Oxidative phosphorylation, Toluene degradation, Butanoate metabolism, Reductive carboxylate cycle (CO2 fixation), Two-component system |
| PEM_01221 | buj: BurJV3_1584 | K00239 | succinate dehydrogenase flavoprotein subunit | ko00623 | EC:1.3.99.1 | COG1053 | Oxidative phosphorylation, Toluene degradation, Butanoate metabolism, Reductive carboxylate cycle (CO2 fixation), Two-component system |
| PEM_01222 | buj: BurJV3_1585 | K00240 | succinate dehydrogenase iron-sulfur protein | ko00623 | EC:1.3.99.1 | COG0479 | Citrate cycle (TCA cycle), Oxidative phosphorylation, Toluene degradation, Butanoate metabolism Reductive carboxylate cycle (CO2 fixation) |
| PEM_01261 | fra: Francci3_2664 | K00517 | beta-carotene 15,15'-monooxygenase | ko00363 | N/A | COG2124 | Bisphenol degradation Aminobenzoate degradation Tropane, piperidine and pyridine alkaloid biosynthesis |
| PEM_01283 | btk:BT9727_2565 | K00680 | phospholipid:diacylglycerol acyltransferase | ko00626 | N/A | N/A | Naphthalene degradation, Aminobenzoate degradation, Ethylbenzene degradation, Limonene and pinene degradation |
| PEM_01320 | sml: Smlt2023 | K00799 | glutathione S-transferase | ko00980 | EC:2.5.1.18 | COG0625 | Glutathione metabolism and Metabolism of xenobiotics by cytochrome P458 |
| PEM_01345 | lsl: LSL_0995 | K00100 | 1-deoxy-D-xylulose-5-phosphate reductoisomerase | ko00625 | N/A | COG0111;COG0677;COG0743;COG1063 | Fructose and mannose metabolism, Bisphenol degradation, Linoleic acid metabolism, Chloroalkane and chloroalkene degradation, Butanoate metabolism |
| PEM_01355 | pna: Pnap_0726 | K00114 | alcohol dehydrogenase (cytochrome c) | ko00625 | EC:1.1.2.8 | N/A | Glycolysis / Gluconeogenesis, Chloroalkane and chloroalkene degradation, Propanoate metabolism |
| PEM_01385 | bcz: BCZK1828 | K00517 | beta-carotene 15,15'-monooxygenase | ko00363 | N/A | COG2124 | Bisphenol degradation, Polycyclic aromatic hydrocarbon degradation Aminobenzoate degradation Limonene and pinene degradation Stilbenoid, diarylheptanoid and gingerol biosynthesis |
| PEM_01385 | rxy: Rxyl_3089 | K00494 | alkanal monooxygenase (FMN-linked) | N/A | EC:1.14.14.3 | N/A |  |
| PEM_01386 | bur: Bcep18194_B1532 | K04091 | alkanesulfonate monooxygenase | N/A | EC:1.14.14.5 | COG2141 |  |
| PEM_01386 | bps: BPSS1672 | K00492 | nitric-oxide synthase, bacterial | ko00363 | N/A | COG0654 |  |
| PEM_01404 | pen: PSEEN3116 | K00599 | trans-aconitate 2-methyltransferase | ko00624 | N/A | COG0500 | Histidine metabolism, Tyrosine metabolism, Selenoamino acid metabolism, Polycyclic aromatic hydrocarbon degradation |
| PEM_01407 | sml: Smlt2132 | K00128 | aldehyde dehydrogenase (NAD+) | ko00625 | EC:1.2.1.3 | COG1012 | Glycolysis / Gluconeogenesis, Pentose and glucuronate interconversions, Ascorbate and aldarate metabolism, Fatty acid metabolism, Valine, leucine and isoleucine degradation, Lysine degradation, Arginine and proline metabolism, Histidine metabolism, Tryptophan metabolism, beta-Alanine metabolism, Glycerolipid metabolism, Pyruvate metabolism, Chloroalkane and chloroalkene degradation, Propanoate metabolism, Limonene and pinene degradation |
| PEM_01439 | rec: RHECIAT_CH0002186 | K01113 | alkaline phosphatase D | ko00627 | EC:3.1.3.1 | COG3540 |  |
| PEM_01462 | abo: ABO_0067 | K00100 | 1-deoxy-D-xylulose-5-phosphate reductoisomerase | ko00625 | N/A | COG0111;COG0677;COG0743;COG1063 | Fructose and mannose metabolism, Bisphenol degradation, Linoleic acid metabolism, Chloroalkane and chloroalkene degradation, Butanoate metabolism |
| PEM_01474 | bme: BMEI0164 | K01759 | lactoylglutathione lyase | ko00620 | EC:4.4.1.5 | COG0346 |  |
| PEM_01632 | gox: GOX2267 | K00680 | phospholipid:diacylglycerol acyltransferase | ko00626 | N/A | N/A | Naphthalene degradation, Aminobenzoate degradation, Ethylbenzene degradation, Limonene and pinene degradation |
| PEM_01654 | msm: MSMEG_6370 | K01607 | 4-carboxymuconolactone decarboxylase | ko00362 | EC:4.1.1.44 | COG0599 |  |
| PEM_01677 | dru: Desru_2528 | K00121 | S-(hydroxymethyl)glutathione dehydrogenase / alcohol dehydrogenase | ko00625 | EC:1.1.1.284;1.1.1.1 | COG1062 | Glycolysis / Gluconeogenesis, Fatty acid metabolism, Tyrosine metabolism, Chloroalkane and chloroalkene degradation, Naphthalene degradation, Methane metabolism, Retinol metabolism, Metabolism of xenobiotics by cytochrome P450, Drug metabolism - cytochrome P450 |
| PEM_01677 | sen: SACE_5586 | K00100 | 1-deoxy-D-xylulose-5-phosphate reductoisomerase | ko00625 | N/A | COG0111;COG0677;COG0743;COG1063 | Fructose and mannose metabolism, Bisphenol degradation, Linoleic acid metabolism, Chloroalkane and chloroalkene degradation, Butanoate metabolism |
| PEM_01677 | chu: CHU_1246 | K00001 | alcohol dehydrogenase | ko00625 | EC:1.1.1.1 | COG0604;COG1062;COG1064;COG1454 | Chloroalkane and chloroalkene degradation, |
| PEM_01733 | vfi: VF_A0640 | K01759 | lactoylglutathione lyase | ko00620 | EC:4.4.1.5 | COG0346 |  |
| PEM_01834 | bat: BAS3185 | K00517 | beta-carotene 15,15'-monooxygenase | ko00363 | N/A | COG2124 | Bisphenol degradation Aminobenzoate degradation Tropane, piperidine and pyridine alkaloid biosynthesis |
| PEM_01834 | reu: Reut_C5909 | K00494 | alkanal monooxygenase (FMN-linked) | N/A | EC:1.14.14.3 | N/A |  |
| PEM_01910 | sro: Sros_7956 | K01563 | haloalkane dehalogenase | ko00361 | EC:3.8.1.5 | COG0596 | Chlorocyclohexane and chlorobenzene degradation, Chloroalkane and chloroalkene degradation |
| PEM_01910 | pac:PPA1620 | K01066 | esterase / lipase | ko00363 | EC:3.1.1.- | COG0657 | Bisphenol degradation Aminobenzoate degradation Tropane, piperidine and pyridine alkaloid biosynthesis |
| PEM_01939 | fal: FRAAL3541 | K04091 | alkanesulfonate monooxygenase | N/A | EC:1.14.14.5 | COG2141 |  |
| PEM_01939 | bce: BC3378 | K00517 | beta-carotene 15,15'-monooxygenase | ko00363 | N/A | COG2124 | Bisphenol degradation, Polycyclic aromatic hydrocarbon degradation Aminobenzoate degradation Limonene and pinene degradation Stilbenoid, diarylheptanoid and gingerol biosynthesis |
| PEM_01939 | cgl: NCgl2137 | K00494 | alkanal monooxygenase (FMN-linked) | N/A | EC:1.14.14.3 | N/A |  |
| PEM_01943 | azo: azo1939 | K00492 | nitric-oxide synthase, bacterial | ko00363 | N/A | COG0654 | Chlorocyclohexane and chlorobenzene degradation Bisphenol degradation Toluene degradation Polycyclic aromatic hydrocarbon degradation Naphthalene degradation Aminobenzoate degradation and Limonene and pinene degradation |
| PEM_01959 | zmo: ZMO1721 | K01759 | lactoylglutathione lyase | ko00620 | EC:4.4.1.5 | COG0346 |  |
| PEM_01968 | azo: azo2619 | K00599 | trans-aconitate 2-methyltransferase | ko00624 | N/A | COG0500 | Histidine metabolism, Tyrosine metabolism, Selenoamino acid metabolism, Polycyclic aromatic hydrocarbon degradation |
| PEM_02001 | gfo: GFO_0971 | K00258 | isoquinoline 1-oxidoreductase | ko00363 | N/A | COG3000 | Bisphenol degradation, DDT degradation, Limonene and pinene degradation |
| PEM_02049 | buj: BurJV3_2486 | K10680 | N-ethylmaleimide reductase | ko00633 | EC:1.-.-.- | COG1902 | Nitrotoluene degradation |
| PEM_02082 | sen: SACE_3584 | K00492 | nitric-oxide synthase, bacterial/pentachlorophenol monooxygenase | ko00363 | N/A | COG0654 | Chlorocyclohexane and chlorobenzene degradation Bisphenol degradation Toluene degradation Polycyclic aromatic hydrocarbon degradation Naphthalene degradation |
| PEM_02090 | azo: azo3317 | K00001 | alcohol dehydrogenase | ko00625 | EC:1.1.1.1 | COG0604;COG1062;COG1064;COG1454 | Glycolysis / Gluconeogenesis, Fatty acid metabolism, Tyrosine metabolism, Chloroalkane and chloroalkene degradation, Naphthalene degradation, Methane metabolism, Retinol metabolism, Metabolism of xenobiotics by cytochrome P450, Drug metabolism - cytochrome P450 |
| PEM_02112 | ret: RHE_PE00074 | K01564 | haloalkane dehalogenase | ko00625 | N/A | N/A | Chloroalkane and chloroalkene degradation, Atrazine degradation |
| PEM_02161 | gox: GOX1462 | K00100 | 1-deoxy-D-xylulose-5-phosphate reductoisomerase | ko00625 | N/A | COG0111;COG0677;COG0743;COG1063 | Fructose and mannose metabolism, Bisphenol degradation, Linoleic acid metabolism, Chloroalkane and chloroalkene degradation, Butanoate metabolism |
| PEM_02171 | smt: Smal_2609 | K00257 | isoquinoline 1-oxidoreductase | ko00626 | N/A | COG1960 | Geraniol degradation, Naphthalene degradation |
| PEM_02176 | fra: Francci3_1054 | K00680 | phospholipid: diacylglycerol acyltransferase | ko00626 | N/A | N/A | Naphthalene degradation, Aminobenzoate degradation, Ethylbenzene degradation, Limonene and pinene degradation, Tyrosine metabolism |
| PEM_02193 | reh: H16_A1564 | K00680 | phospholipid:diacylglycerol acyltransferase | ko00626 | N/A | N/A | Naphthalene degradation, Aminobenzoate degradation, Ethylbenzene degradation, Limonene and pinene degradation, Tyrosine metabolism |
| PEM_02199 | lsl: LSL_1531 | K00100 | 1-deoxy-D-xylulose-5-phosphate reductoisomerase | ko00625 | N/A | COG0111;COG0677;COG0743;COG1063 | Fructose and mannose metabolism, Bisphenol degradation, Linoleic acid metabolism, Chloroalkane and chloroalkene degradation, Butanoate metabolism |
| PEM_02199 | lps: LPST_C2861 | K00244 | fumarate reductase flavoprotein subunit | ko00623 | EC:1.3.99.1 | COG1053 | Oxidative phosphorylation, Toluene degradation, Butanoate metabolism, Reductive carboxylate cycle (CO2 fixation), Two-component system |
| PEM_02202 | sml: Smlt3208 | K00799 | glutathione S-transferase | ko00980 | EC:2.5.1.18 | COG0625 | Metabolism of xenobiotics by cytochrome P450 Drug metabolism - cytochrome P450 |
| PEM_02214 | smt: Smal_2653 | K00799 | glutathione S-transferase | ko00980 | EC:2.5.1.18 | COG0625 | Glutathione metabolism and Metabolism of xenobiotics by cytochrome P456 |
| PEM_02218 | msm: MSMEG_6370 | K01607 | 4-carboxymuconolactone decarboxylase | ko00362 | EC:4.1.1.44 | COG0599 |  |
| PEM_02305 | buj: BurJV3_2901 | K01061 | carboxymethylenebutenolidase | ko00623 | EC:3.1.1.45 | COG0412 | Chlorocyclohexane and chlorobenzene degradation, Fluorobenzoate degradation, Toluene degradation, Peptidoglycan biosynthesis |
| PEM_02315 | afm: AFUA_5G10090 | K00599 | trans-aconitate 2-methyltransferase | ko00624 | N/A | COG0500 | Histidine metabolism, Tyrosine metabolism, Selenoamino acid metabolism, Polycyclic aromatic hydrocarbon degradation |
| PEM_02316 | chu: CHU_0562 | K00258 | isoquinoline 1-oxidoreductase | ko00363 | N/A | COG3000 | Bisphenol degradation, DDT degradation, Limonene and pinene degradation |
| PEM_02405 | rba: RB13026 | K00517 | beta-carotene 15,15'-monooxygenase | ko00363 | N/A | COG2124 |  |
| PEM_02405 | sro: Sros_5419 | K00480 | salicylate hydroxylase | ko00626 | EC:1.14.13.1 | COG0654 | Naphthalene degradation, Salicylate degradation , and Dioxin degradation |
| PEM_02450 | syg: sync_2405 | K00257 | isoquinoline 1-oxidoreductase | ko00626 | N/A | COG1960 | Geraniol degradation, Naphthalene degradation |
| PEM_02458 | abo: ABO_1231 | K00001 | alcohol dehydrogenase | ko00625 | EC:1.1.1.1 | COG0604;COG1062;COG1064;COG1454 | Glycolysis / Gluconeogenesis, Fatty acid metabolism, Tyrosine metabolism, Chloroalkane and chloroalkene degradation, Naphthalene degradation, Methane metabolism, |
| PEM_02489 | ava: Ava_2685 | K01561 | haloacetate dehalogenase | ko00625 | EC:3.8.1.3 | N/A | Chlorocyclohexane and chlorobenzene degradation, Chloroalkane and chloroalkene degradation |
| PEM_02532 | pdx: Psed_0071 | K08195 | MFS transporter, AAHS family, 4-hydroxybenzoate transporter | N/A | N/A | COG0477 |  |
| PEM_02573 | gfo: GFO_2080 | K00680 | phospholipid:diacylglycerol acyltransferase | ko00626 | N/A | N/A | Naphthalene degradation, Aminobenzoate degradation, Ethylbenzene degradation, Limonene and pinene degradation, Tyrosine metabolism |
| PEM_02603 | gfo: GFO_2080 | K00680 | phospholipid:diacylglycerol acyltransferase | ko00626 | N/A | N/A | Naphthalene degradation, Aminobenzoate degradation, Ethylbenzene degradation, Limonene and pinene degradation |
| PEM_02603 | gfo: GFO_2080 | K00680 | phospholipid:diacylglycerol acyltransferase | ko00626 | N/A | N/A | Naphthalene degradation, Aminobenzoate degradation, Ethylbenzene degradation, Limonene and pinene degradation, Tyrosine metabolism |
| PEM_02626 | sml: Smlt3734 | K00799 | glutathione S-transferase | ko00980 | EC:2.5.1.18 | COG0625 | Glutathione metabolism and Metabolism of xenobiotics by cytochrome P451 |
| PEM_02673 | reu: Reut_A0332 | K01826 | 5-carboxymethyl-2-hydroxymuconate isomerase | ko00362 | EC:5.3.3.10 | COG3232 |  |
| PEM_02695 | msm: MSMEG_6310 | K01041 | glutaconate CoA-transferase, subunit B | ko00626 | N/A | N/A | Geraniol degradation, Naphthalene degradation |
| PEM_02738 | vfi: VF_A0640 | K01759 | lactoylglutathione lyase | ko00620 | EC:4.4.1.5 | COG0346 |  |
| PEM_02753 | rha: RHA1_ro02535 | K00496 | alkane 1-monooxygenase | ko00071 | EC:1.14.15.3 | N/A | Fatty acid metabolism |
| PEM_02799 | hma: rrnAC3101 | K00599 | trans-aconitate 2-methyltransferase | ko00624 | N/A | COG0500 | Histidine metabolism, Tyrosine metabolism, Selenoamino acid metabolism, Polycyclic aromatic hydrocarbon degradation |
| PEM_02803 | bpm: BURPS1710b_1565 | K00100 | 1-deoxy-D-xylulose-5-phosphate reductoisomerase | ko00625 | N/A | COG0111;COG0677;COG0743;COG1063 | Fructose and mannose metabolism, Bisphenol degradation, Linoleic acid metabolism, Chloroalkane and chloroalkene degradation, Butanoate metabolism |
| PEM_02845 | xtr: 496688 | K13299 | glutathione S-transferase kappa 1 | ko00980 | EC:2.5.1.18 | N/A | Glutathione metabolism and Metabolism of xenobiotics by cytochrome P460 |
| PEM_02851 | sml: Smlt3978 | K00121 | S-(hydroxymethyl)glutathione dehydrogenase / alcohol dehydrogenase | ko00625 | EC:1.1.1.284;1.1.1.1 | COG1062 | Glycolysis / Gluconeogenesis, Fatty acid metabolism, Tyrosine metabolism, Chloroalkane and chloroalkene degradation, Naphthalene degradation, Methane metabolism, |
| PEM_02854 | agr: AGROH133_07653 | K10680 | N-ethylmaleimide reductase | ko00633 | EC:1.-.-.- | COG1902 | Nitrotoluene degradation |
| PEM_02855 | azo: azo2616 | K01759 | lactoylglutathione lyase | ko00620 | EC:4.4.1.5 | COG0346 |  |
| PEM_02905 | llm: llmg_0271 | K00100 | 1-deoxy-D-xylulose-5-phosphate reductoisomerase | ko00625 | N/A | COG0111;COG0677;COG0743;COG1063 | Fructose and mannose metabolism, Bisphenol degradation, Linoleic acid metabolism, Chloroalkane and chloroalkene degradation, Butanoate metabolism |
| PEM_02905 | Dme: Dmel_CG3609 | K00078 | dihydrodiol dehydrogenase / D-xylose 1-dehydrogenase (NADP) | ko00980 | EC:1.3.1.20;1.1.1.179 | N/A | Pentose and glucuronate interconversions, Metabolism of xenobiotics by cytochrome P450 |
| PEM_02938 | mdo:100030403 | K00078 | dihydrodiol dehydrogenase / D-xylose 1-dehydrogenase (NADP) | ko00980 | EC:1.3.1.20;1.1.1.179 | N/A | Pentose and glucuronate interconversions, Metabolism of xenobiotics by cytochrome P450 |
| PEM_02960 | sml: Smlt4096 | K01759 | lactoylglutathione lyase | ko00620 | EC:4.4.1.5 | COG0346 |  |
| PEM_02961 | bba: Bd0456 | K01759 | lactoylglutathione lyase | ko00620 | EC:4.4.1.5 | COG0346 |  |
| PEM_03003 | lic: LIC13133 | K00100 | 1-deoxy-D-xylulose-5-phosphate reductoisomerase | ko00625 | N/A | COG0111;COG0677;COG0743;COG1063 | Fructose and mannose metabolism, Bisphenol degradation, Linoleic acid metabolism, Chloroalkane and chloroalkene degradation, Butanoate metabolism |
| PEM_03007 | reh: H16_A0274 | K01759 | lactoylglutathione lyase | ko00620 | EC:4.4.1.5 | COG0346 |  |
| PEM_03013 | buj: BurJV3_3604 | K00799 | glutathione S-transferase | ko00980 | EC:2.5.1.18 | COG0625 | Glutathione metabolism and Metabolism of xenobiotics by cytochrome P452 |
| PEM_03054 | xac: XAC3819 | K00799 | glutathione S-transferase | ko00980 | EC:2.5.1.18 | COG0625 | Glutathione metabolism and Metabolism of xenobiotics by cytochrome P455 |
| PEM_03079 | rha: RHA1_ro02470 | K00622 | arylamine N-acetyltransferase | ko00633 | EC:2.3.1.5 | COG2162 | Caffeine metabolism, Nitrotoluene degradation, Drug metabolism - other enzymes |
| PEM_03100 | pen: PSEEN0706 | K00680 | phospholipid:diacylglycerol acyltransferase | ko00626 | N/A | N/A | Naphthalene degradation, Aminobenzoate degradation, Ethylbenzene degradation, Limonene and pinene degradation, Tyrosine metabolism |
| PEM_03103 | ckl: CKL_0532 | K01066 | esterase / lipase | ko00363 | EC:3.1.1.- | COG0657 | Bisphenol degradation Aminobenzoate degradation Tropane, piperidine and pyridine alkaloid biosynthesis |
| PEM_03132 | gox: GOX0646 | K00100 | 1-deoxy-D-xylulose-5-phosphate reductoisomerase | ko00625 | N/A | COG0111;COG0677;COG0743;COG1063 | Fructose and mannose metabolism, Bisphenol degradation, Linoleic acid metabolism, Chloroalkane and chloroalkene degradation, Butanoate metabolism |
| PEM_03133 | azo: azo0148 | K00680 | phospholipid:diacylglycerol acyltransferase | ko00626 | N/A | N/A | Naphthalene degradation, Aminobenzoate degradation, Ethylbenzene degradation, Limonene and pinene degradation, Tyrosine metabolism |
| PEM_03152 | btl: BALH_2771 | K00680 | phospholipid:diacylglycerol acyltransferase | ko00626 | N/A | N/A | Naphthalene degradation, Aminobenzoate degradation, Ethylbenzene degradation, Limonene and pinene degradation, Tyrosine metabolism |
| PEM_03167 | pfl: PFL_4316 | K01564 | haloalkane dehalogenase | ko00625 | N/A | N/A | Chloroalkane and chloroalkene degradation, Atrazine degradation |
| PEM_03194 | hne: HNE_1435 | K00100 | 1-deoxy-D-xylulose-5-phosphate reductoisomerase | ko00625 | N/A | COG0111;COG0677;COG0743;COG1063 | Fructose and mannose metabolism, Bisphenol degradation, Linoleic acid metabolism, Chloroalkane and chloroalkene degradation, Butanoate metabolism |
| PEM_03223 | msm: MSMEG_6370 | K01607 | 4-carboxymuconolactone decarboxylase | ko00362 | EC:4.1.1.44 | COG0599 |  |
| PEM_03239 | ret: RHE_CH00346 | K00462 | biphenyl-2,3-diol 1,2-dioxygenase | ko00361 | EC:1.13.11.39 | N/A | Chlorocyclohexane and chlorobenzene degradation, Dioxin degradation |
| PEM_03239 | zmo: ZMO0030 | K01759 | lactoylglutathione lyase | ko00620 | EC:4.4.1.5 | COG0346 |  |
| PEM_03295 | reh: H16_A3071 | K00680 | phospholipid:diacylglycerol acyltransferase | ko00626 | N/A | N/A | Naphthalene degradation, Aminobenzoate degradation, Ethylbenzene degradation, Limonene and pinene degradation, Tyrosine metabolism |
| PEM_03332 | bba: Bd2246 | K00100 | 1-deoxy-D-xylulose-5-phosphate reductoisomerase | ko00625 | N/A | COG0111;COG0677;COG0743;COG1063 | Fructose and mannose metabolism, Bisphenol degradation, Linoleic acid metabolism, Chloroalkane and chloroalkene degradation, Butanoate metabolism |
| PEM_03361 | bra: BRADO3440 | K00100 | 1-deoxy-D-xylulose-5-phosphate reductoisomerase | ko00625 | N/A | COG0111;COG0677;COG0743;COG1063 | Fructose and mannose metabolism, Bisphenol degradation, Linoleic acid metabolism, Chloroalkane and chloroalkene degradation, Butanoate metabolism |
| PEM_03387 | sml: Smlt4422 | K03179 | 4-hydroxybenzoate octaprenyltransferase | ko00130 | EC:2.5.1.- | COG0382 |  |
| PEM_03421 | cgb: cg0155 | K00680 | phospholipid:diacylglycerol acyltransferase | ko00626 | N/A | N/A | Naphthalene degradation, Aminobenzoate degradation, Ethylbenzene degradation, Limonene and pinene degradation |
| PEM_03444 | sml: Smlt4504 | K00799 | glutathione S-transferase | ko00980 | EC:2.5.1.18 | COG0625 | Metabolism of xenobiotics by cytochrome P450 Drug metabolism - cytochrome P450 |
| PEM_03457 | smt: Smal_3879 | K03380 | phenol 2-monooxygenase | ko00623 | EC:1.14.13.7 | N/A | Chlorocyclohexane and chlorobenzene degradation, Toluene degradation, Aminobenzoate degradation |
| PEM_03467 | bra: BRADO0841 | K00100 | 1-deoxy-D-xylulose-5-phosphate reductoisomerase | ko00625 | N/A | COG0111;COG0677;COG0743;COG1063 | Fructose and mannose metabolism, Bisphenol degradation, Linoleic acid metabolism, Chloroalkane and chloroalkene degradation, Butanoate metabolism |
| PEM_03475 | tth: TTC1376 | K01055 | 3-oxoadipate enol-lactonase or beta-ketoadipate enol-lactone hydrolase | ko00362 | EC:3.1.1.24 | COG0596 |  |
| PEM_03488 | gox: GOX1400 | K00100 | 1-deoxy-D-xylulose-5-phosphate reductoisomerase | ko00625 | N/A | COG0111;COG0677;COG0743;COG1063 | Fructose and mannose metabolism, Bisphenol degradation, Linoleic acid metabolism, Chloroalkane and chloroalkene degradation, Butanoate metabolism |
| PEM_03493 | azo: azo0258 | K00680 | phospholipid:diacylglycerol acyltransferase | ko00626 | N/A | N/A | Naphthalene degradation, Aminobenzoate degradation, Ethylbenzene degradation, Limonene and pinene degradation, Tyrosine metabolism |
| PEM_03569 | mil:ML5_5535 | K01561 | haloacetate dehalogenase | ko00625 | EC:3.8.1.3 | N/A | Chlorocyclohexane and chlorobenzene degradation, Chloroalkane and chloroalkene degradation |
| PEM_03571 | buj: BurJV3_4039 | K05782 | benzoate membrane transport protein | N/A | N/A | COG3135 |  |
| PEM_03597 | Buj: BurJV3_4065 | K07323 | putative toluene tolerance protein | N/A | N/A | COG2854 | toluene degradation |
| PEM_03603 | buj: BurJV3_4071 | K00799 | glutathione S-transferase | ko00980 | EC:2.5.1.18 | COG0625 | Glutathione metabolism and Metabolism of xenobiotics by cytochrome P453 |
| PEM_03604 | gox: GOX0476 | K00100 | 1-deoxy-D-xylulose-5-phosphate reductoisomerase | ko00625 | N/A | COG0111;COG0677;COG0743;COG1063 | Fructose and mannose metabolism, Bisphenol degradation, Linoleic acid metabolism, Chloroalkane and chloroalkene degradation, Butanoate metabolism |
| PEM_03604 | reh:H16_B1699 | K00001 | alcohol dehydrogenase | ko00625 | EC:1.1.1.1 | COG0604;COG1062;COG1064;COG1454 | Glycolysis / Gluconeogenesis, Fatty acid metabolism, Tyrosine metabolism, Chloroalkane and chloroalkene degradation, Naphthalene degradation, Methane metabolism, |
| PEM_03645 | buj: BurJV3_0027 | K13953 | alcohol dehydrogenase, propanol-preferring | ko00625 | EC:1.1.1.1 | COG1064 | Glycolysis / Gluconeogenesis, Fatty acid metabolism, Tyrosine metabolism, Chloroalkane and chloroalkene degradation, Naphthalene degradation, Methane metabolism, |
| PEM_03645 | buj: BurJV3_0027 | K13953 | alcohol dehydrogenase, propanol-preferring | ko00626 | EC:1.1.1.1 | COG1064 | Glycolysis / Gluconeogenesis, Fatty acid metabolism, Tyrosine metabolism, Chloroalkane and chloroalkene degradation, Naphthalene degradation, Methane metabolism, |
| PEM_03654 | sml: Smlt0084 | K09474 | acid phosphatase (class A) | ko00627 | EC:3.1.3.2 | COG0671 |  |
| PEM_03667 | rba: RB2355 | K00680 | phospholipid:diacylglycerol acyltransferase | ko00626 | N/A | N/A | Naphthalene degradation, Aminobenzoate degradation, Ethylbenzene degradation, Limonene and pinene degradation, Tyrosine metabolism |
| PEM_03754 | bra: BRADO0018 | K00100 | 1-deoxy-D-xylulose-5-phosphate reductoisomerase | ko00625 | N/A | COG0111;COG0677;COG0743;COG1063 | Fructose and mannose metabolism, Bisphenol degradation, Linoleic acid metabolism, Chloroalkane and chloroalkene degradation, Butanoate metabolism |
| PEM_03755 | rlg: Rleg_0809 | K00799 | glutathione S-transferase | ko00980 | EC:2.5.1.18 | COG0625 | Glutathione metabolism and Metabolism of xenobiotics by cytochrome P450 |
| PEM_03772 | buj: BurJV3_0117 | K00626 | acetyl-CoA C-acetyltransferase | ko00362 | EC:2.3.1.9 | COG0183 |  |
| PEM_03776 | vfi: VF_0102 | K00680 | phospholipid:diacylglycerol acyltransferase | ko00626 | N/A | N/A | Naphthalene degradation, Aminobenzoate degradation, Ethylbenzene degradation, Limonene and pinene degradation |
| PEM_03803 | sml: Smlt0199 | K00252 | glutaryl-CoA dehydrogenase | ko00362 | EC:1.3.99.7 | COG1960 |  |
| PEM_03811 | sml: Smlt0206 | K01563 | haloalkane dehalogenase | ko00361 | EC:3.8.1.5 | COG0596 | Chlorocyclohexane and chlorobenzene degradation, Chloroalkane and chloroalkene degradation |
| PEM_03814 | lic: LIC13133 | K00100 | 1-deoxy-D-xylulose-5-phosphate reductoisomerase | ko00625 | N/A | COG0111; COG0677; COG0743; COG1063 | Fructose and mannose metabolism, Bisphenol degradation, Linoleic acid metabolism, Chloroalkane and chloroalkene degradation, Butanoate metabolism |
| PEM_03823 | xac: XAC2121 | K00599 | trans-aconitate 2-methyltransferase | ko00624 | N/A | COG0500 | Histidine metabolism, Tyrosine metabolism, Selenoamino acid metabolism, Polycyclic aromatic hydrocarbon degradation |
| PEM_03827 | aci: ACIAD2050 | K00680 | phospholipid: diacylglycerol acyltransferase | ko00626 | N/A | N/A | Naphthalene degradation, Aminobenzoate degradation, Ethylbenzene degradation, Limonene and pinene degradation, Tyrosine metabolism |
| PEM_03855 | amd: AMED_8366 | K09461 | anthraniloyl-CoA monooxygenase | ko00627 | EC:1.14.13.40 | N/A |  |
| PEM_03865 | smt: Smal_0220 | K00257 | isoquinoline 1-oxidoreductase | ko00626 | N/A | COG1960 | Geraniol degradation, Naphthalene degradation |
| PEM_03866 | buj: BurJV3_0222 | K01692 | enoyl-CoA hydratase | ko00362 | EC:4.2.1.17 | COG1024 |  |
| PEM_03939 | bba: Bd3653 | K01077 | alkaline phosphatase | ko00627 | EC:3.1.3.1 | COG1785 |  |
| PEM_02489 | tpr: Tpau_3844 | K05714 | 2-hydroxy-6-ketonona-2,4-dienedioic acid hydrolase | ko00360 | EC:3.7.1.- | COG0596 |  |
| PEM_01386 | bur: Bcep18194_B1532 | K04091 | alkanesulfonate monooxygenase | N/A | EC:1.14.14.5 | COG2141 |  |
| PEM_01939 | fal: FRAAL3541 | K04091 | alkanesulfonate monooxygenase | N/A | EC:1.14.14.5 | COG2141 |  |
| PEM_02671 | lch: Lcho_1335 | K03862 | vanillate monooxygenase | ko00627 | EC:1.14.13.82 | COG2146 |  |
| PEM_02399 | buj: BurJV3_2988 | K09456 | putative acyl-CoA dehydrogenase | N/A | N/A | COG1960 |  |
| PEM_00186 | sml: Smlt0609 | K01800 | maleylacetoacetate isomerase | ko00643 | EC:5.2.1.2 | COG0625 |  |
| PEM_03309 | sml: Smlt4329 | K00451 | homogentisate 1,2-dioxygenase | ko00643 | EC:1.13.11.5 | COG3508 |  |

**Table S2 Identified Genomic Islands in Pemsol**

| List of Islands | Island start | Island end | Length | Method | Gene name | Gene ID | Locus | Gene start | Gene end | Strand | Product |
| --- | --- | --- | --- | --- | --- | --- | --- | --- | --- | --- | --- |
| Island 1 | 77964 | 100189 | 22225 | Predicted by at least one method | WP_119706165.1 |  | PEM_RS00005 | 1 | 4373402 | 1 | IS3 family transposase |
|  | 77964 | 100189 | 22225 | Predicted by at least one method | WP_119706038.1 |  | PEM_RS00360 | 77964 | 80135 | 1 | ATP-binding protein |
|  | 77964 | 100189 | 22225 | Predicted by at least one method | WP_119706039.1 |  | PEM_RS00365 | 80132 | 81469 | 1 | hypothetical protein |
|  | 77964 | 100189 | 22225 | Predicted by at least one method | WP_119706040.1 |  | PEM_RS00370 | 81818 | 83560 | -1 | DUF262 domain-containing protein |
|  | 77964 | 100189 | 22225 | Predicted by at least one method | WP_119706166.1 | vsr | PEM_RS00375 | 84206 | 84661 | -1 | DNA mismatch endonuclease Vsr |
|  | 77964 | 100189 | 22225 | Predicted by at least one method | WP_119706167.1 |  | PEM_RS00380 | 84763 | 86535 | -1 | DNA cytosine methyltransferase |
|  | 77964 | 100189 | 22225 | Predicted by at least one method | WP_119706041.1 |  | PEM_RS00385 | 86493 | 87716 | 1 | DUF3883 domain-containing protein |
|  | 77964 | 100189 | 22225 | Predicted by at least one method | WP_119706042.1 |  | PEM_RS00390 | 87858 | 88058 | -1 | hypothetical protein |
|  | 77964 | 100189 | 22225 | Predicted by at least one method | WP_119706043.1 |  | PEM_RS00395 | 88292 | 88660 | -1 | hypothetical protein |
|  | 77964 | 100189 | 22225 | Predicted by at least one method | WP_119706044.1 |  | PEM_RS00400 | 89148 | 89363 | -1 | hypothetical protein |
|  | 77964 | 100189 | 22225 | Predicted by at least one method | WP_119706045.1 |  | PEM_RS00405 | 89534 | 90178 | 1 | DUF1629 domain-containing protein |
|  | 77964 | 100189 | 22225 | Predicted by at least one method | WP_119706168.1 |  | PEM_RS00410 | 90277 | 90918 | 1 | DUF1629 domain-containing protein |
|  | 77964 | 100189 | 22225 | Predicted by at least one method | WP_119706046.1 |  | PEM_RS00415 | 91006 | 91650 | 1 | DUF1629 domain-containing protein |
|  | 77964 | 100189 | 22225 | Predicted by at least one method | WP_119706047.1 |  | PEM_RS00420 | 92025 | 92681 | 1 | DUF1629 domain-containing protein |
|  | 77964 | 100189 | 22225 | Predicted by at least one method | WP_119706048.1 |  | PEM_RS00425 | 92801 | 93430 | 1 | DUF1629 domain-containing protein |
|  | 77964 | 100189 | 22225 | Predicted by at least one method | WP_119706049.1 |  | PEM_RS00430 | 93728 | 94369 | 1 | DUF1629 domain-containing protein |
|  | 77964 | 100189 | 22225 | Predicted by at least one method | WP_119706050.1 |  | PEM_RS00435 | 95109 | 95303 | -1 | hypothetical protein |
|  | 77964 | 100189 | 22225 | Predicted by at least one method | WP_119706051.1 |  | PEM_RS00440 | 95340 | 97772 | 1 | hypothetical protein |
|  | 77964 | 100189 | 22225 | Predicted by at least one method | WP_119706052.1 |  | PEM_RS00445 | 98041 | 98454 | 1 | hypothetical protein |
|  | 77964 | 100189 | 22225 | Predicted by at least one method | WP_119706053.1 |  | PEM_RS00450 | 98533 | 99030 | -1 | hypothetical protein |
|  | 77964 | 100189 | 22225 | Predicted by at least one method | WP_119706054.1 |  | PEM_RS00455 | 99320 | 100189 | -1 | hypothetical protein |
| Island 2 | 212532 | 225320 | 12788 | Predicted by at least one method | WP_119706165.1 |  | PEM_RS00005 | 1 | 4373402 | 1 | IS3 family transposase |
|  | 212532 | 225320 | 12788 | Predicted by at least one method | WP_061480473.1 |  | PEM_RS00985 | 211348 | 212535 | 1 | cystathionine gamma-synthase |
|  | 212532 | 225320 | 12788 | Predicted by at least one method | WP_111126855.1 |  | PEM_RS00990 | 212532 | 213365 | 1 | ABC transporter permease |
|  | 212532 | 225320 | 12788 | Predicted by at least one method | WP_111126853.1 |  | PEM_RS00995 | 213355 | 214695 | 1 | ABC transporter ATP-binding protein |
|  | 212532 | 225320 | 12788 | Predicted by at least one method | WP_111126851.1 |  | PEM_RS01000 | 214692 | 216875 | 1 | class I SAM-dependent methyltransferase |
|  | 212532 | 225320 | 12788 | Predicted by at least one method | WP_111126849.1 |  | PEM_RS01005 | 216857 | 219058 | 1 | glycosyltransferase |
|  | 212532 | 225320 | 12788 | Predicted by at least one method | WP_111126847.1 |  | PEM_RS01010 | 219082 | 221364 | 1 | glycosyltransferase family 2 protein |
|  | 212532 | 225320 | 12788 | Predicted by at least one method | WP_100477532.1 |  | PEM_RS01015 | 221447 | 222439 | -1 | glycosyltransferase family 2 protein |
|  | 212532 | 225320 | 12788 | Predicted by at least one method | WP_111126845.1 |  | PEM_RS01020 | 222454 | 224331 | -1 | hypothetical protein |
|  | 212532 | 225320 | 12788 | Predicted by at least one method | WP_111126842.1 |  | PEM_RS01025 | 224868 | 225320 | -1 | GtrA family protein |
| Island 3 | 320461 | 325069 | 4608 | Predicted by at least one method | WP_119706165.1 |  | PEM_RS00005 | 1 | 4373402 | 1 | IS3 family transposase |
|  | 320461 | 325069 | 4608 | Predicted by at least one method | WP_032953932.1 |  | PEM_RS01415 | 320461 | 320835 | -1 | hypothetical protein |
|  | 320461 | 325069 | 4608 | Predicted by at least one method | WP_111128408.1 |  | PEM_RS01420 | 321148 | 322008 | -1 | hypothetical protein |
|  | 320461 | 325069 | 4608 | Predicted by at least one method | WP_069139489.1 |  | PEM_RS01425 | 322163 | 323125 | -1 | hypothetical protein |
|  | 320461 | 325069 | 4608 | Predicted by at least one method | WP_111128406.1 |  | PEM_RS01430 | 323138 | 323785 | -1 | hypothetical protein |
|  | 320461 | 325069 | 4608 | Predicted by at least one method | WP_111128404.1 |  | PEM_RS01435 | 323996 | 325069 | 1 | hypothetical protein |
| Island 4 | 635499 | 639638 | 4139 | Predicted by at least one method | WP_119706165.1 |  | PEM_RS00005 | 1 | 4373402 | 1 | IS3 family transposase |
|  | 635499 | 639638 | 4139 | Predicted by at least one method | WP_005415491.1 |  | PEM_RS02880 | 635499 | 636248 | 1 | hypothetical protein |
|  | 635499 | 639638 | 4139 | Predicted by at least one method | WP_111127569.1 |  | PEM_RS02885 | 636564 | 639329 | -1 | hypothetical protein |
|  | 635499 | 639638 | 4139 | Predicted by at least one method | WP_005415493.1 |  | PEM_RS02890 | 639330 | 639638 | -1 | hypothetical protein |
| Island 5 | 940931 | 946303 | 5372 | Predicted by at least one method | WP_119706165.1 |  | PEM_RS00005 | 1 | 4373402 | 1 | IS3 family transposase |
|  | 940931 | 946303 | 5372 | Predicted by at least one method | WP_065196531.1 |  | PEM_RS04305 | 940931 | 941335 | 1 | hypothetical protein |
|  | 940931 | 946303 | 5372 | Predicted by at least one method | WP_065196530.1 |  | PEM_RS04310 | 942041 | 942445 | 1 | hypothetical protein |
|  | 940931 | 946303 | 5372 | Predicted by at least one method | WP_095377789.1 |  | PEM_RS04315 | 943936 | 944307 | 1 | DUF596 domain-containing protein |
|  | 940931 | 946303 | 5372 | Predicted by at least one method | WP_111127959.1 |  | PEM_RS04320 | 944490 | 944837 | 1 | DUF596 domain-containing protein |
|  | 940931 | 946303 | 5372 | Predicted by at least one method | WP_095377791.1 |  | PEM_RS04325 | 945024 | 945911 | 1 | DUF2628 domain-containing protein |
|  | 940931 | 946303 | 5372 | Predicted by at least one method | WP_095377792.1 |  | PEM_RS04330 | 945911 | 946303 | 1 | hypothetical protein |
| Island 6 | 1139449 | 1143710 | 4261 | Predicted by at least one method | WP_119706165.1 |  | PEM_RS00005 | 1 | 4373402 | 1 | IS3 family transposase |
|  | 1139449 | 1143710 | 4261 | Predicted by at least one method | WP_005415918.1 |  | PEM_RS05185 | 1139449 | 1141011 | -1 | sensor kinase |
|  | 1139449 | 1143710 | 4261 | Predicted by at least one method | WP_032954065.1 |  | PEM_RS05190 | 1141470 | 1141958 | 1 | hypothetical protein |
|  | 1139449 | 1143710 | 4261 | Predicted by at least one method | WP_065186143.1 |  | PEM_RS05195 | 1142118 | 1143710 | -1 | hypothetical protein |
| Island 7 | 1374377 | 1384472 | 10095 | Predicted by at least one method | WP_119706165.1 |  | PEM_RS00005 | 1 | 4373402 | 1 | IS3 family transposase |
|  | 1374377 | 1384472 | 10095 | Predicted by at least one method | WP_111128307.1 |  | PEM_RS06395 | 1374377 | 1374859 | -1 | hypothetical protein |
|  | 1374377 | 1384472 | 10095 | Predicted by at least one method | WP_111128305.1 |  | PEM_RS06400 | 1375057 | 1375308 | 1 | hypothetical protein |
|  | 1374377 | 1384472 | 10095 | Predicted by at least one method | WP_111128304.1 |  | PEM_RS06405 | 1375324 | 1375569 | -1 | hypothetical protein |
|  | 1374377 | 1384472 | 10095 | Predicted by at least one method | WP_111128302.1 |  | PEM_RS06410 | 1375619 | 1376203 | 1 | hypothetical protein |
|  | 1374377 | 1384472 | 10095 | Predicted by at least one method |  |  | PEM_RS06415 | 1376371 | 1377657 | -1 | DNA cytosine methyltransferase |
|  | 1374377 | 1384472 | 10095 | Predicted by at least one method | WP_111128301.1 |  | PEM_RS06420 | 1377711 | 1378001 | -1 | hypothetical protein |
|  | 1374377 | 1384472 | 10095 | Predicted by at least one method | WP_111128299.1 |  | PEM_RS06425 | 1378462 | 1379196 | 1 | hypothetical protein |
|  | 1374377 | 1384472 | 10095 | Predicted by at least one method | WP_111128298.1 |  | PEM_RS06430 | 1379535 | 1379903 | 1 | hypothetical protein |
|  | 1374377 | 1384472 | 10095 | Predicted by at least one method | WP_111128297.1 |  | PEM_RS06435 | 1380063 | 1380611 | 1 | hypothetical protein |
|  | 1374377 | 1384472 | 10095 | Predicted by at least one method |  |  | PEM_RS06440 | 1380702 | 1381634 | 1 | hypothetical protein |
|  | 1374377 | 1384472 | 10095 | Predicted by at least one method | WP_099475748.1 |  | PEM_RS06445 | 1381791 | 1382105 | 1 | helix-turn-helix domain-containing protein |
|  | 1374377 | 1384472 | 10095 | Predicted by at least one method | WP_111128295.1 |  | PEM_RS06450 | 1382124 | 1382495 | -1 | hypothetical protein |
|  | 1374377 | 1384472 | 10095 | Predicted by at least one method | WP_111128294.1 |  | PEM_RS06455 | 1382751 | 1383263 | 1 | hypothetical protein |
|  | 1374377 | 1384472 | 10095 | Predicted by at least one method | WP_111128292.1 |  | PEM_RS06460 | 1383267 | 1384472 | -1 | DUF4102 domain-containing protein |
| Island 8 | 1378462 | 1383263 | 4801 | Predicted by at least one method | WP_119706165.1 |  | PEM_RS00005 | 1 | 4373402 | 1 | IS3 family transposase |
|  | 1378462 | 1383263 | 4801 | Predicted by at least one method | WP_111128299.1 |  | PEM_RS06425 | 1378462 | 1379196 | 1 | hypothetical protein |
|  | 1378462 | 1383263 | 4801 | Predicted by at least one method | WP_111128298.1 |  | PEM_RS06430 | 1379535 | 1379903 | 1 | hypothetical protein |
|  | 1378462 | 1383263 | 4801 | Predicted by at least one method | WP_111128297.1 |  | PEM_RS06435 | 1380063 | 1380611 | 1 | hypothetical protein |
|  | 1378462 | 1383263 | 4801 | Predicted by at least one method |  |  | PEM_RS06440 | 1380702 | 1381634 | 1 | hypothetical protein |
|  | 1378462 | 1383263 | 4801 | Predicted by at least one method | WP_099475748.1 |  | PEM_RS06445 | 1381791 | 1382105 | 1 | helix-turn-helix domain-containing protein |
|  | 1378462 | 1383263 | 4801 | Predicted by at least one method | WP_111128295.1 |  | PEM_RS06450 | 1382124 | 1382495 | -1 | hypothetical protein |
|  | 1378462 | 1383263 | 4801 | Predicted by at least one method | WP_111128294.1 |  | PEM_RS06455 | 1382751 | 1383263 | 1 | hypothetical protein |
| Island 9 | 1779697 | 1790053 | 10356 | Predicted by at least one method | WP_119706165.1 |  | PEM_RS00005 | 1 | 4373402 | 1 | IS3 family transposase |
|  | 1779697 | 1790053 | 10356 | Predicted by at least one method | WP_111127669.1 |  | PEM_RS08250 | 1779697 | 1780176 | 1 | N-acetyltransferase |
|  | 1779697 | 1790053 | 10356 | Predicted by at least one method | WP_119706088.1 |  | PEM_RS08255 | 1780247 | 1780567 | 1 | hypothetical protein |
|  | 1779697 | 1790053 | 10356 | Predicted by at least one method | WP_080352922.1 |  | PEM_RS08260 | 1781071 | 1781430 | 1 | hypothetical protein |
|  | 1779697 | 1790053 | 10356 | Predicted by at least one method | WP_111127665.1 |  | PEM_RS08265 | 1781468 | 1781947 | -1 | hypothetical protein |
|  | 1779697 | 1790053 | 10356 | Predicted by at least one method | WP_080352923.1 |  | PEM_RS08270 | 1782041 | 1782337 | -1 | hypothetical protein |
|  | 1779697 | 1790053 | 10356 | Predicted by at least one method | WP_049414795.1 |  | PEM_RS08275 | 1782515 | 1782775 | -1 | hypothetical protein |
|  | 1779697 | 1790053 | 10356 | Predicted by at least one method | WP_080352924.1 |  | PEM_RS08280 | 1783130 | 1783834 | -1 | hypothetical protein |
|  | 1779697 | 1790053 | 10356 | Predicted by at least one method | WP_080352925.1 |  | PEM_RS08285 | 1783824 | 1784471 | -1 | DUF4433 domain-containing protein |
|  | 1779697 | 1790053 | 10356 | Predicted by at least one method | WP_049414796.1 |  | PEM_RS08290 | 1784720 | 1785421 | -1 | hypothetical protein |
|  | 1779697 | 1790053 | 10356 | Predicted by at least one method | WP_119706089.1 |  | PEM_RS08295 | 1785592 | 1786347 | 1 | hypothetical protein |
|  | 1779697 | 1790053 | 10356 | Predicted by at least one method | WP_111127009.1 |  | PEM_RS08300 | 1786408 | 1786932 | -1 | transporter |
|  | 1779697 | 1790053 | 10356 | Predicted by at least one method | WP_119706090.1 |  | PEM_RS08305 | 1787523 | 1787879 | 1 | hypothetical protein |
|  | 1779697 | 1790053 | 10356 | Predicted by at least one method | WP_111128490.1 |  | PEM_RS08310 | 1787880 | 1788350 | 1 | hypothetical protein |
|  | 1779697 | 1790053 | 10356 | Predicted by at least one method | WP_111128489.1 |  | PEM_RS08315 | 1788390 | 1789016 | -1 | nucleotidyltransferase family protein |
|  | 1779697 | 1790053 | 10356 | Predicted by at least one method | WP_111128487.1 |  | PEM_RS08320 | 1788992 | 1790053 | -1 | XdhC/CoxI family protein |
| Island 10 | 1810472 | 1815583 | 5111 | Predicted by at least one method | WP_119706165.1 |  | PEM_RS00005 | 1 | 4373402 | 1 | IS3 family transposase |
|  | 1810472 | 1815583 | 5111 | Predicted by at least one method | WP_032954207.1 |  | PEM_RS08450 | 1810472 | 1810693 | -1 | hypothetical protein |
|  | 1810472 | 1815583 | 5111 | Predicted by at least one method | WP_111128462.1 |  | PEM_RS08455 | 1811019 | 1811396 | 1 | response regulator |
|  | 1810472 | 1815583 | 5111 | Predicted by at least one method | WP_005416573.1 |  | PEM_RS08460 | 1811412 | 1811840 | -1 | BLUF domain-containing protein |
|  | 1810472 | 1815583 | 5111 | Predicted by at least one method | WP_005416574.1 |  | PEM_RS08465 | 1812019 | 1812708 | -1 | glycosyltransferase family 2 protein |
|  | 1810472 | 1815583 | 5111 | Predicted by at least one method | WP_111128460.1 |  | PEM_RS08470 | 1812705 | 1813292 | -1 | methyltransferase domain-containing protein |
|  | 1810472 | 1815583 | 5111 | Predicted by at least one method | WP_111174906.1 |  | PEM_RS08475 | 1813289 | 1814047 | -1 | PIG-L family deacetylase |
|  | 1810472 | 1815583 | 5111 | Predicted by at least one method | WP_111128456.1 |  | PEM_RS08480 | 1814035 | 1814976 | -1 | acyl-CoA dehydrogenase |
|  | 1810472 | 1815583 | 5111 | Predicted by at least one method | WP_065186752.1 |  | PEM_RS08485 | 1815086 | 1815583 | -1 | hypothetical protein |
| Island 11  1816906-1820994 | 1816906 | 1820994 | 4088 | Predicted by at least one method | WP_119706165.1 |  | PEM_RS00005 | 1 | 4373402 | 1 | IS3 family transposase |
|  | 1816906 | 1820994 | 4088 | Predicted by at least one method | WP_053496592.1 |  | PEM_RS08495 | 1816906 | 1817094 | 1 | hypothetical protein |
|  | 1816906 | 1820994 | 4088 | Predicted by at least one method | WP_111128452.1 |  | PEM_RS08500 | 1817194 | 1817832 | 1 | hypothetical protein |
|  | 1816906 | 1820994 | 4088 | Predicted by at least one method | WP_005416582.1 |  | PEM_RS08505 | 1817836 | 1818387 | 1 | cysteine hydrolase |
|  | 1816906 | 1820994 | 4088 | Predicted by at least one method | WP_111128450.1 |  | PEM_RS08510 | 1818403 | 1818636 | -1 | hypothetical protein |
|  | 1816906 | 1820994 | 4088 | Predicted by at least one method | WP_080104316.1 |  | PEM_RS08515 | 1818778 | 1819188 | -1 | hypothetical protein |
|  | 1816906 | 1820994 | 4088 | Predicted by at least one method | WP_111128448.1 |  | PEM_RS08520 | 1819270 | 1819956 | -1 | hypothetical protein |
|  | 1816906 | 1820994 | 4088 | Predicted by at least one method | WP_005416586.1 |  | PEM_RS08525 | 1820011 | 1820994 | -1 | LacI family DNA-binding transcriptional regulator |
|  | 1932729 | 1952883 | 20154 | Predicted by at least one method | WP_119706165.1 |  | PEM_RS00005 | 1 | 4373402 | 1 | IS3 family transposase |
| Island 12 | 1932729 | 1952883 | 20154 | Predicted by at least one method | WP_111129284.1 |  | PEM_RS09020 | 1932649 | 1933020 | -1 | hypothetical protein |
|  | 1932729 | 1952883 | 20154 | Predicted by at least one method | WP_111129286.1 |  | PEM_RS09025 | 1933557 | 1934615 | 1 | hypothetical protein |
|  | 1932729 | 1952883 | 20154 | Predicted by at least one method | WP_081280448.1 |  | PEM_RS09030 | 1934642 | 1935382 | -1 | hypothetical protein |
|  | 1932729 | 1952883 | 20154 | Predicted by at least one method | WP_065196355.1 |  | PEM_RS09035 | 1935379 | 1936410 | -1 | hypothetical protein |
|  | 1932729 | 1952883 | 20154 | Predicted by at least one method | WP_111129288.1 |  | PEM_RS09040 | 1936435 | 1939002 | -1 | fimbrial biogenesis outer membrane usher protein |
|  | 1932729 | 1952883 | 20154 | Predicted by at least one method | WP_111129290.1 |  | PEM_RS09045 | 1939092 | 1939820 | -1 | molecular chaperone |
|  | 1932729 | 1952883 | 20154 | Predicted by at least one method | WP_111129292.1 |  | PEM_RS09050 | 1939890 | 1940402 | -1 | hypothetical protein |
|  | 1932729 | 1952883 | 20154 | Predicted by at least one method |  |  | PEM_RS09055 | 1940800 | 1941324 | -1 | molecular chaperone |
|  | 1932729 | 1952883 | 20154 | Predicted by at least one method | WP_111129294.1 |  | PEM_RS09060 | 1941548 | 1941838 | -1 | hypothetical protein |
|  | 1932729 | 1952883 | 20154 | Predicted by at least one method | WP_111129295.1 |  | PEM_RS09065 | 1941882 | 1942676 | -1 | molecular chaperone |
|  | 1932729 | 1952883 | 20154 | Predicted by at least one method | WP_111129297.1 |  | PEM_RS09070 | 1942673 | 1943704 | -1 | fimbrial protein |
|  | 1932729 | 1952883 | 20154 | Predicted by at least one method | WP_111129299.1 |  | PEM_RS09075 | 1943707 | 1946316 | -1 | fimbrial biogenesis outer membrane usher protein |
|  | 1932729 | 1952883 | 20154 | Predicted by at least one method | WP_111129301.1 |  | PEM_RS09080 | 1946403 | 1947149 | -1 | molecular chaperone |
|  | 1932729 | 1952883 | 20154 | Predicted by at least one method | WP_111129303.1 |  | PEM_RS09085 | 1947230 | 1947784 | -1 | type 1 fimbrial protein |
|  | 1932729 | 1952883 | 20154 | Predicted by at least one method | WP_111129305.1 |  | PEM_RS09090 | 1947951 | 1948634 | -1 | porin family protein |
|  | 1932729 | 1952883 | 20154 | Predicted by at least one method | WP_065196365.1 |  | PEM_RS09095 | 1948704 | 1949600 | -1 | hypothetical protein |
|  | 1932729 | 1952883 | 20154 | Predicted by at least one method | WP_065177739.1 |  | PEM_RS09100 | 1949848 | 1950069 | -1 | hypothetical protein |
|  | 1932729 | 1952883 | 20154 | Predicted by at least one method | WP_111129307.1 |  | PEM_RS09105 | 1950158 | 1950835 | -1 | response regulator |
|  | 1932729 | 1952883 | 20154 | Predicted by at least one method | WP_065177737.1 |  | PEM_RS09110 | 1950913 | 1951560 | 1 | DNA-binding response regulator |
|  | 1932729 | 1952883 | 20154 | Predicted by at least one method | WP_111129309.1 |  | PEM_RS09115 | 1951584 | 1954955 | -1 | sensor histidine kinase |
|  | 1941548 | 1947784 | 6236 | Predicted by at least one method | WP_119706165.1 |  | PEM_RS00005 | 1 | 4373402 | 1 | IS3 family transposase |
| Island 13 | 1941548 | 1947784 | 6236 | Predicted by at least one method | WP_111129294.1 |  | PEM_RS09060 | 1941548 | 1941838 | -1 | hypothetical protein |
|  | 1941548 | 1947784 | 6236 | Predicted by at least one method | WP_111129295.1 |  | PEM_RS09065 | 1941882 | 1942676 | -1 | molecular chaperone |
|  | 1941548 | 1947784 | 6236 | Predicted by at least one method | WP_111129297.1 |  | PEM_RS09070 | 1942673 | 1943704 | -1 | fimbrial protein |
|  | 1941548 | 1947784 | 6236 | Predicted by at least one method | WP_111129299.1 |  | PEM_RS09075 | 1943707 | 1946316 | -1 | fimbrial biogenesis outer membrane usher protein |
|  | 1941548 | 1947784 | 6236 | Predicted by at least one method | WP_111129301.1 |  | PEM_RS09080 | 1946403 | 1947149 | -1 | molecular chaperone |
|  | 1941548 | 1947784 | 6236 | Predicted by at least one method | WP_111129303.1 |  | PEM_RS09085 | 1947230 | 1947784 | -1 | type 1 fimbrial protein |
|  | 2782453 | 2791255 | 8802 | Predicted by at least one method | WP_119706165.1 |  | PEM_RS00005 | 1 | 4373402 | 1 | IS3 family transposase |
| Island 14 | 2782453 | 2791255 | 8802 | Predicted by at least one method | WP_111128791.1 |  | PEM_RS12890 | 2782453 | 2783646 | -1 | MFS transporter |
|  | 2782453 | 2791255 | 8802 | Predicted by at least one method | WP_005417015.1 |  | PEM_RS12895 | 2783740 | 2784669 | 1 | LysR family transcriptional regulator |
|  | 2782453 | 2791255 | 8802 | Predicted by at least one method | WP_111128836.1 |  | PEM_RS12900 | 2784759 | 2786216 | -1 | amidohydrolase |
|  | 2782453 | 2791255 | 8802 | Predicted by at least one method | WP_065185738.1 |  | PEM_RS12905 | 2786271 | 2786972 | -1 | ankyrin repeat domain-containing protein |
|  | 2782453 | 2791255 | 8802 | Predicted by at least one method | WP_005417024.1 |  | PEM_RS12910 | 2787088 | 2787969 | 1 | LysR family transcriptional regulator |
|  | 2782453 | 2791255 | 8802 | Predicted by at least one method | WP_111128793.1 |  | PEM_RS12915 | 2788132 | 2788605 | 1 | DUF3806 domain-containing protein |
|  | 2782453 | 2791255 | 8802 | Predicted by at least one method | WP_111128794.1 |  | PEM_RS12920 | 2788781 | 2789218 | 1 | DUF3806 domain-containing protein |
|  | 2782453 | 2791255 | 8802 | Predicted by at least one method | WP_111128796.1 |  | PEM_RS12925 | 2789267 | 2790427 | -1 | cytosine deaminase |
|  | 2782453 | 2791255 | 8802 | Predicted by at least one method | WP_111128798.1 |  | PEM_RS12930 | 2790611 | 2791255 | 1 | hypothetical protein |
|  | 2918164 | 2924684 | 6520 | Predicted by at least one method | WP_119706165.1 |  | PEM_RS00005 | 1 | 4373402 | 1 | IS3 family transposase |
| Island 15 | 2918164 | 2924684 | 6520 | Predicted by at least one method | WP_111128020.1 |  | PEM_RS13485 | 2918164 | 2918346 | 1 | hypothetical protein |
|  | 2918164 | 2924684 | 6520 | Predicted by at least one method | WP_111128022.1 |  | PEM_RS13490 | 2919063 | 2922989 | -1 | RHS repeat protein |
|  | 2918164 | 2924684 | 6520 | Predicted by at least one method | WP_100473728.1 |  | PEM_RS13495 | 2924298 | 2924684 | -1 | hypothetical protein |
|  | 2930323 | 2938024 | 7701 | Predicted by at least one method | WP_119706165.1 |  | PEM_RS00005 | 1 | 4373402 | 1 | IS3 family transposase |
| Island 16 | 2930323 | 2938024 | 7701 | Predicted by at least one method | WP_111128025.1 |  | PEM_RS13530 | 2930323 | 2930541 | -1 | hypothetical protein |
|  | 2930323 | 2938024 | 7701 | Predicted by at least one method | WP_111174855.1 |  | PEM_RS13535 | 2930964 | 2932208 | -1 | HlyD family efflux transporter periplasmic adaptor subunit |
|  | 2930323 | 2938024 | 7701 | Predicted by at least one method | WP_111128026.1 |  | PEM_RS13540 | 2932205 | 2934316 | -1 | peptidase domain-containing ABC transporter |
|  | 2930323 | 2938024 | 7701 | Predicted by at least one method | WP_065185273.1 |  | PEM_RS13545 | 2934686 | 2935258 | -1 | CPBP family intramembrane metalloprotease |
|  | 2930323 | 2938024 | 7701 | Predicted by at least one method | WP_065185274.1 |  | PEM_RS13550 | 2935462 | 2935665 | -1 | hypothetical protein |
|  | 2930323 | 2938024 | 7701 | Predicted by at least one method | WP_065185275.1 |  | PEM_RS13555 | 2936037 | 2936372 | -1 | hypothetical protein |
|  | 2930323 | 2938024 | 7701 | Predicted by at least one method |  |  | PEM_RS13560 | 2936369 | 2938024 | -1 | site-specific integrase |
|  | 2995282 | 3000315 | 5033 | Predicted by at least one method | WP_119706165.1 |  | PEM_RS00005 | 1 | 4373402 | 1 | IS3 family transposase |
| Island 17 | 2995282 | 3000315 | 5033 | Predicted by at least one method | WP_111128209.1 |  | PEM_RS13805 | 2995282 | 3000315 | -1 | hemagluttinin domain-containing protein |
|  | 3342574 | 3349702 | 7128 | Predicted by at least one method | WP_119706165.1 |  | PEM_RS00005 | 1 | 4373402 | 1 | IS3 family transposase |
| Island 18 | 3342574 | 3349702 | 7128 | Predicted by at least one method | WP_069139687.1 |  | PEM_RS15320 | 3342574 | 3342993 | -1 | H-NS histone family protein |
|  | 3342574 | 3349702 | 7128 | Predicted by at least one method | WP_019660763.1 |  | PEM_RS15325 | 3343112 | 3343504 | 1 | DoxX family protein |
|  | 3342574 | 3349702 | 7128 | Predicted by at least one method | WP_111128068.1 |  | PEM_RS15330 | 3343523 | 3345241 | -1 | serine hydrolase |
|  | 3342574 | 3349702 | 7128 | Predicted by at least one method | WP_111128059.1 |  | PEM_RS15335 | 3345360 | 3346001 | -1 | hypothetical protein |
|  | 3342574 | 3349702 | 7128 | Predicted by at least one method | WP_111128061.1 |  | PEM_RS15340 | 3347029 | 3347682 | 1 | hypothetical protein |
|  | 3342574 | 3349702 | 7128 | Predicted by at least one method | WP_005419578.1 |  | PEM_RS15345 | 3348134 | 3348538 | 1 | hypothetical protein |
|  | 3342574 | 3349702 | 7128 | Predicted by at least one method | WP_111128063.1 |  | PEM_RS15350 | 3348566 | 3349702 | 1 | hypothetical protein |
|  | 3673418 | 3680554 | 7136 | Predicted by at least one method | WP_119706165.1 |  | PEM_RS00005 | 1 | 4373402 | 1 | IS3 family transposase |
|  | 3673418 | 3680554 | 7136 | Predicted by at least one method | WP_111129559.1 | arr | PEM_RS16990 | 3673418 | 3673852 | 1 | NAD(+)--rifampin ADP-ribosyltransferase |
| Island 19 | 3673418 | 3680554 | 7136 | Predicted by at least one method | WP_111129560.1 |  | PEM_RS16995 | 3673845 | 3674291 | -1 | DUF4279 domain-containing protein |
|  | 3673418 | 3680554 | 7136 | Predicted by at least one method | WP_111129561.1 |  | PEM_RS17000 | 3674562 | 3674864 | -1 | hypothetical protein |
|  | 3673418 | 3680554 | 7136 | Predicted by at least one method | WP_049417059.1 |  | PEM_RS17005 | 3675201 | 3675566 | -1 | hypothetical protein |
|  | 3673418 | 3680554 | 7136 | Predicted by at least one method | WP_111129562.1 |  | PEM_RS17010 | 3675621 | 3676223 | -1 | DUF998 domain-containing protein |
|  | 3673418 | 3680554 | 7136 | Predicted by at least one method | WP_111129563.1 |  | PEM_RS17015 | 3676377 | 3677339 | 1 | hypothetical protein |
|  | 3673418 | 3680554 | 7136 | Predicted by at least one method | WP_111129564.1 |  | PEM_RS17020 | 3677814 | 3678170 | 1 | hypothetical protein |
|  | 3673418 | 3680554 | 7136 | Predicted by at least one method | WP_111129565.1 |  | PEM_RS17025 | 3678216 | 3678746 | 1 | cold-shock protein |
|  | 3673418 | 3680554 | 7136 | Predicted by at least one method | WP_111129566.1 |  | PEM_RS17030 | 3678823 | 3679143 | -1 | hypothetical protein |
|  | 3673418 | 3680554 | 7136 | Predicted by at least one method | WP_065186542.1 |  | PEM_RS17035 | 3679314 | 3679523 | -1 | hypothetical protein |
|  | 3673418 | 3680554 | 7136 | Predicted by at least one method | WP_065186543.1 |  | PEM_RS17040 | 3679520 | 3680554 | -1 | hypothetical protein |
|  | 4117495 | 4130922 | 13427 | Predicted by at least one method | WP_119706165.1 |  | PEM_RS00005 | 1 | 4373402 | 1 | IS3 family transposase |
|  | 4117495 | 4130922 | 13427 | Predicted by at least one method | WP_005420957.1 |  | PEM_RS18940 | 4117495 | 4117809 | 1 | hypothetical protein |
| Island 20 | 4117495 | 4130922 | 13427 | Predicted by at least one method | WP_111127188.1 |  | PEM_RS18945 | 4117914 | 4119959 | 1 | TonB-dependent receptor |
|  | 4117495 | 4130922 | 13427 | Predicted by at least one method | WP_075675705.1 |  | PEM_RS18950 | 4120365 | 4120943 | 1 | histidine phosphatase family protein |
|  | 4117495 | 4130922 | 13427 | Predicted by at least one method | WP_087921115.1 |  | PEM_RS18955 | 4121020 | 4121448 | -1 | hypothetical protein |
|  | 4117495 | 4130922 | 13427 | Predicted by at least one method | WP_119706135.1 |  | PEM_RS18960 | 4122507 | 4124927 | 1 | transposase |
|  | 4117495 | 4130922 | 13427 | Predicted by at least one method | WP_049441968.1 |  | PEM_RS18965 | 4124927 | 4125928 | 1 | ATP-binding protein |
|  | 4117495 | 4130922 | 13427 | Predicted by at least one method | WP_119706136.1 |  | PEM_RS18970 | 4125938 | 4126168 | 1 | hypothetical protein |
|  | 4117495 | 4130922 | 13427 | Predicted by at least one method | WP_119706137.1 |  | PEM_RS18975 | 4126168 | 4127187 | 1 | ATP-binding protein |
|  | 4117495 | 4130922 | 13427 | Predicted by at least one method | WP_075675701.1 |  | PEM_RS18980 | 4129204 | 4129572 | -1 | H-NS histone family protein |
|  | 4117495 | 4130922 | 13427 | Predicted by at least one method | WP_119706138.1 |  | PEM_RS18985 | 4129825 | 4130922 | 1 | hypothetical protein |
|  | 4120365 | 4139831 | 19466 | Predicted by at least one method | WP_119706165.1 |  | PEM_RS00005 | 1 | 4373402 | 1 | IS3 family transposase |
|  | 4120365 | 4139831 | 19466 | Predicted by at least one method | WP_075675705.1 |  | PEM_RS18950 | 4120365 | 4120943 | 1 | histidine phosphatase family protein |
| Island 21 | 4120365 | 4139831 | 19466 | Predicted by at least one method | WP_087921115.1 |  | PEM_RS18955 | 4121020 | 4121448 | -1 | hypothetical protein |
|  | 4120365 | 4139831 | 19466 | Predicted by at least one method | WP_119706135.1 |  | PEM_RS18960 | 4122507 | 4124927 | 1 | transposase |
|  | 4120365 | 4139831 | 19466 | Predicted by at least one method | WP_049441968.1 |  | PEM_RS18965 | 4124927 | 4125928 | 1 | ATP-binding protein |
|  | 4120365 | 4139831 | 19466 | Predicted by at least one method | WP_119706136.1 |  | PEM_RS18970 | 4125938 | 4126168 | 1 | hypothetical protein |
|  | 4120365 | 4139831 | 19466 | Predicted by at least one method | WP_119706137.1 |  | PEM_RS18975 | 4126168 | 4127187 | 1 | ATP-binding protein |
|  | 4120365 | 4139831 | 19466 | Predicted by at least one method | WP_075675701.1 |  | PEM_RS18980 | 4129204 | 4129572 | -1 | H-NS histone family protein |
|  | 4120365 | 4139831 | 19466 | Predicted by at least one method | WP_119706138.1 |  | PEM_RS18985 | 4129825 | 4130922 | 1 | hypothetical protein |
|  | 4120365 | 4139831 | 19466 | Predicted by at least one method | WP_049468424.1 |  | PEM_RS18990 | 4130974 | 4132953 | 1 | BCCT family transporter |
|  | 4120365 | 4139831 | 19466 | Predicted by at least one method | WP_119706139.1 |  | PEM_RS18995 | 4133076 | 4134320 | 1 | DUF2254 domain-containing protein |
|  | 4120365 | 4139831 | 19466 | Predicted by at least one method |  |  | PEM_RS19000 | 4134256 | 4135500 | -1 | class A beta-lactamase-related serine hydrolase |
|  | 4120365 | 4139831 | 19466 | Predicted by at least one method | WP_119706140.1 |  | PEM_RS19005 | 4135806 | 4136174 | -1 | TonB-dependent receptor |
|  | 4120365 | 4139831 | 19466 | Predicted by at least one method | WP_083663114.1 |  | PEM_RS19010 | 4136346 | 4137107 | -1 | sulfite exporter TauE/SafE family protein |
|  | 4120365 | 4139831 | 19466 | Predicted by at least one method | WP_075675697.1 |  | PEM_RS19015 | 4137126 | 4137563 | -1 | TIGR01244 family phosphatase |
|  | 4120365 | 4139831 | 19466 | Predicted by at least one method | WP_119706141.1 |  | PEM_RS19020 | 4137560 | 4137991 | -1 | YeeE/YedE family protein |
|  | 4120365 | 4139831 | 19466 | Predicted by at least one method | WP_119706142.1 |  | PEM_RS19025 | 4137988 | 4138428 | -1 | YeeE/YedE family protein |
|  | 4120365 | 4139831 | 19466 | Predicted by at least one method | WP_032965476.1 |  | PEM_RS19030 | 4138425 | 4139294 | -1 | MBL fold metallo-hydrolase |
|  | 4120365 | 4139831 | 19466 | Predicted by at least one method | WP_049441866.1 |  | PEM_RS19035 | 4139538 | 4139831 | 1 | transcriptional regulator |
|  | 4122807 | 4130052 | 7245 | Predicted by at least one method | WP_119706165.1 |  | PEM_RS00005 | 1 | 4373402 | 1 | IS3 family transposase |
|  | 4122807 | 4130052 | 7245 | Predicted by at least one method | WP_119706135.1 |  | PEM_RS18960 | 4122507 | 4124927 | 1 | transposase |
|  | 4122807 | 4130052 | 7245 | Predicted by at least one method | WP_049441968.1 |  | PEM_RS18965 | 4124927 | 4125928 | 1 | ATP-binding protein |
|  | 4122807 | 4130052 | 7245 | Predicted by at least one method | WP_119706136.1 |  | PEM_RS18970 | 4125938 | 4126168 | 1 | hypothetical protein |
|  | 4122807 | 4130052 | 7245 | Predicted by at least one method | WP_119706137.1 |  | PEM_RS18975 | 4126168 | 4127187 | 1 | ATP-binding protein |
|  | 4122807 | 4130052 | 7245 | Predicted by at least one method | WP_075675701.1 |  | PEM_RS18980 | 4129204 | 4129572 | -1 | H-NS histone family protein |
|  | 4122807 | 4130052 | 7245 | Predicted by at least one method | WP_119706138.1 |  | PEM_RS18985 | 4129825 | 4130922 | 1 | hypothetical protein |
|  | 4135477 | 4158355 | 22878 | Predicted by at least one method | WP_119706165.1 |  | PEM_RS00005 | 1 | 4373402 | 1 | IS3 family transposase |
|  | 4135477 | 4158355 | 22878 | Predicted by at least one method |  |  | PEM_RS19000 | 4134256 | 4135500 | -1 | class A beta-lactamase-related serine hydrolase |
| Island 22 | 4135477 | 4158355 | 22878 | Predicted by at least one method | WP_119706140.1 |  | PEM_RS19005 | 4135806 | 4136174 | -1 | TonB-dependent receptor |
|  | 4135477 | 4158355 | 22878 | Predicted by at least one method | WP_083663114.1 |  | PEM_RS19010 | 4136346 | 4137107 | -1 | sulfite exporter TauE/SafE family protein |
|  | 4135477 | 4158355 | 22878 | Predicted by at least one method | WP_075675697.1 |  | PEM_RS19015 | 4137126 | 4137563 | -1 | TIGR01244 family phosphatase |
|  | 4135477 | 4158355 | 22878 | Predicted by at least one method | WP_119706141.1 |  | PEM_RS19020 | 4137560 | 4137991 | -1 | YeeE/YedE family protein |
|  | 4135477 | 4158355 | 22878 | Predicted by at least one method | WP_119706142.1 |  | PEM_RS19025 | 4137988 | 4138428 | -1 | YeeE/YedE family protein |
|  | 4135477 | 4158355 | 22878 | Predicted by at least one method | WP_032965476.1 |  | PEM_RS19030 | 4138425 | 4139294 | -1 | MBL fold metallo-hydrolase |
|  | 4135477 | 4158355 | 22878 | Predicted by at least one method | WP_049441866.1 |  | PEM_RS19035 | 4139538 | 4139831 | 1 | transcriptional regulator |
|  | 4135477 | 4158355 | 22878 | Predicted by at least one method | WP_119706143.1 |  | PEM_RS19040 | 4140029 | 4141996 | -1 | TonB-dependent receptor |
|  | 4135477 | 4158355 | 22878 | Predicted by at least one method | WP_119706144.1 |  | PEM_RS19045 | 4142069 | 4142479 | -1 | MerR family transcriptional regulator |
|  | 4135477 | 4158355 | 22878 | Predicted by at least one method | WP_119706145.1 |  | PEM_RS19050 | 4142565 | 4143191 | 1 | cation transporter |
|  | 4135477 | 4158355 | 22878 | Predicted by at least one method | WP_032959372.1 |  | PEM_RS19055 | 4143501 | 4143944 | -1 | endoribonuclease L-PSP |
|  | 4135477 | 4158355 | 22878 | Predicted by at least one method | WP_119706146.1 |  | PEM_RS19060 | 4144118 | 4145005 | -1 | LysR family transcriptional regulator |
|  | 4135477 | 4158355 | 22878 | Predicted by at least one method | WP_119706147.1 |  | PEM_RS19065 | 4145149 | 4145901 | 1 | SDR family oxidoreductase |
|  | 4135477 | 4158355 | 22878 | Predicted by at least one method | WP_119706148.1 |  | PEM_RS19070 | 4146098 | 4146772 | 1 | glutathione S-transferase family protein |
|  | 4135477 | 4158355 | 22878 | Predicted by at least one method | WP_119706149.1 |  | PEM_RS19075 | 4146867 | 4147475 | 1 | integrase |
|  | 4135477 | 4158355 | 22878 | Predicted by at least one method | WP_119706150.1 |  | PEM_RS19080 | 4147607 | 4147786 | 1 | hypothetical protein |
|  | 4135477 | 4158355 | 22878 | Predicted by at least one method | WP_119706151.1 |  | PEM_RS19085 | 4148327 | 4149199 | 1 | hypothetical protein |
|  | 4135477 | 4158355 | 22878 | Predicted by at least one method | WP_119706152.1 |  | PEM_RS19090 | 4149264 | 4149662 | 1 | hypothetical protein |
|  | 4135477 | 4158355 | 22878 | Predicted by at least one method | WP_119706153.1 |  | PEM_RS19095 | 4149649 | 4149903 | 1 | hypothetical protein |
|  | 4135477 | 4158355 | 22878 | Predicted by at least one method | WP_115875143.1 |  | PEM_RS19100 | 4150084 | 4150989 | -1 | LysR family transcriptional regulator |
|  | 4135477 | 4158355 | 22878 | Predicted by at least one method | WP_032959369.1 |  | PEM_RS19105 | 4151130 | 4152527 | 1 | amidohydrolase |
|  | 4135477 | 4158355 | 22878 | Predicted by at least one method | WP_115875139.1 |  | PEM_RS19110 | 4152536 | 4153015 | 1 | DMT family transporter |
|  | 4135477 | 4158355 | 22878 | Predicted by at least one method | WP_075675695.1 |  | PEM_RS19115 | 4153265 | 4153972 | 1 | AbiV family abortive infection protein |
|  | 4135477 | 4158355 | 22878 | Predicted by at least one method | WP_119706154.1 |  | PEM_RS19120 | 4154047 | 4154835 | -1 | hypothetical protein |
|  | 4135477 | 4158355 | 22878 | Predicted by at least one method | WP_119706155.1 |  | PEM_RS19125 | 4155417 | 4155938 | -1 | hypothetical protein |
|  | 4135477 | 4158355 | 22878 | Predicted by at least one method | WP_119706156.1 |  | PEM_RS19130 | 4156165 | 4157991 | -1 | DUF2813 domain-containing protein |
| Island 23 | 4142565 | 4170025 | 27460 | Predicted by at least one method | WP_119706165.1 |  | PEM_RS00005 | 1 | 4373402 | 1 | IS3 family transposase |
|  | 4142565 | 4170025 | 27460 | Predicted by at least one method | WP_119706145.1 |  | PEM_RS19050 | 4142565 | 4143191 | 1 | cation transporter |
|  | 4142565 | 4170025 | 27460 | Predicted by at least one method | WP_032959372.1 |  | PEM_RS19055 | 4143501 | 4143944 | -1 | endoribonuclease L-PSP |
|  | 4142565 | 4170025 | 27460 | Predicted by at least one method | WP_119706146.1 |  | PEM_RS19060 | 4144118 | 4145005 | -1 | LysR family transcriptional regulator |
|  | 4142565 | 4170025 | 27460 | Predicted by at least one method | WP_119706147.1 |  | PEM_RS19065 | 4145149 | 4145901 | 1 | SDR family oxidoreductase |
|  | 4142565 | 4170025 | 27460 | Predicted by at least one method | WP_119706148.1 |  | PEM_RS19070 | 4146098 | 4146772 | 1 | glutathione S-transferase family protein |
|  | 4142565 | 4170025 | 27460 | Predicted by at least one method | WP_119706149.1 |  | PEM_RS19075 | 4146867 | 4147475 | 1 | integrase |
|  | 4142565 | 4170025 | 27460 | Predicted by at least one method | WP_119706150.1 |  | PEM_RS19080 | 4147607 | 4147786 | 1 | hypothetical protein |
|  | 4142565 | 4170025 | 27460 | Predicted by at least one method | WP_119706151.1 |  | PEM_RS19085 | 4148327 | 4149199 | 1 | hypothetical protein |
|  | 4142565 | 4170025 | 27460 | Predicted by at least one method | WP_119706152.1 |  | PEM_RS19090 | 4149264 | 4149662 | 1 | hypothetical protein |
|  | 4142565 | 4170025 | 27460 | Predicted by at least one method | WP_119706153.1 |  | PEM_RS19095 | 4149649 | 4149903 | 1 | hypothetical protein |
|  | 4142565 | 4170025 | 27460 | Predicted by at least one method | WP_115875143.1 |  | PEM_RS19100 | 4150084 | 4150989 | -1 | LysR family transcriptional regulator |
|  | 4142565 | 4170025 | 27460 | Predicted by at least one method | WP_032959369.1 |  | PEM_RS19105 | 4151130 | 4152527 | 1 | amidohydrolase |
|  | 4142565 | 4170025 | 27460 | Predicted by at least one method | WP_115875139.1 |  | PEM_RS19110 | 4152536 | 4153015 | 1 | DMT family transporter |
|  | 4142565 | 4170025 | 27460 | Predicted by at least one method | WP_075675695.1 |  | PEM_RS19115 | 4153265 | 4153972 | 1 | AbiV family abortive infection protein |
|  | 4142565 | 4170025 | 27460 | Predicted by at least one method | WP_119706154.1 |  | PEM_RS19120 | 4154047 | 4154835 | -1 | hypothetical protein |
|  | 4142565 | 4170025 | 27460 | Predicted by at least one method | WP_119706155.1 |  | PEM_RS19125 | 4155417 | 4155938 | -1 | hypothetical protein |
|  | 4142565 | 4170025 | 27460 | Predicted by at least one method | WP_119706156.1 |  | PEM_RS19130 | 4156165 | 4157991 | -1 | DUF2813 domain-containing protein |
|  | 4142565 | 4170025 | 27460 | Predicted by at least one method | WP_005420952.1 |  | PEM_RS19135 | 4158377 | 4159438 | 1 | two-component sensor histidine kinase |
|  | 4142565 | 4170025 | 27460 | Predicted by at least one method | WP_111127186.1 | ntrC | PEM_RS19140 | 4159431 | 4160879 | 1 | nitrogen regulation protein NR(I) |
|  | 4142565 | 4170025 | 27460 | Predicted by at least one method | WP_065178703.1 |  | PEM_RS19145 | 4160973 | 4161542 | 1 | superoxide dismutase family protein |
|  | 4142565 | 4170025 | 27460 | Predicted by at least one method | WP_053497798.1 |  | PEM_RS19150 | 4161575 | 4162201 | 1 | superoxide dismutase family protein |
|  | 4142565 | 4170025 | 27460 | Predicted by at least one method | WP_065178704.1 |  | PEM_RS19155 | 4162323 | 4163210 | -1 | hypothetical protein |
|  | 4142565 | 4170025 | 27460 | Predicted by at least one method | WP_005420942.1 |  | PEM_RS19160 | 4163304 | 4164599 | 1 | acetyl-CoA C-acetyltransferase |
|  | 4142565 | 4170025 | 27460 | Predicted by at least one method | WP_111127184.1 |  | PEM_RS19165 | 4164760 | 4166055 | -1 | heme biosynthesis protein HemY |
|  | 4142565 | 4170025 | 27460 | Predicted by at least one method | WP_111127182.1 |  | PEM_RS19170 | 4166062 | 4167036 | -1 | hypothetical protein |
|  | 4142565 | 4170025 | 27460 | Predicted by at least one method | WP_111127180.1 |  | PEM_RS19175 | 4167141 | 4167950 | -1 | uroporphyrinogen-III synthase |
|  | 4142565 | 4170025 | 27460 | Predicted by at least one method | WP_005407593.1 |  | PEM_RS19180 | 4167986 | 4168453 | 1 | thioesterase |
|  | 4142565 | 4170025 | 27460 | Predicted by at least one method | WP_111127178.1 |  | PEM_RS19185 | 4168486 | 4168911 | 1 | hypothetical protein |
|  | 4142565 | 4170025 | 27460 | Predicted by at least one method | WP_005420930.1 |  | PEM_RS19190 | 4168969 | 4169406 | 1 | rhodanese-like domain-containing protein |
|  | 4142565 | 4170025 | 27460 | Predicted by at least one method | WP_005407596.1 |  | PEM_RS19195 | 4169507 | 4170025 | 1 | protein-export protein SecB |
| Island 24 | 4143501 | 4150989 | 7488 | Predicted by at least one method | WP_119706165.1 |  | PEM_RS00005 | 1 | 4373402 | 1 | IS3 family transposase |
|  | 4143501 | 4150989 | 7488 | Predicted by at least one method | WP_032959372.1 |  | PEM_RS19055 | 4143501 | 4143944 | -1 | endoribonuclease L-PSP |
|  | 4143501 | 4150989 | 7488 | Predicted by at least one method | WP_119706146.1 |  | PEM_RS19060 | 4144118 | 4145005 | -1 | LysR family transcriptional regulator |
|  | 4143501 | 4150989 | 7488 | Predicted by at least one method | WP_119706147.1 |  | PEM_RS19065 | 4145149 | 4145901 | 1 | SDR family oxidoreductase |
|  | 4143501 | 4150989 | 7488 | Predicted by at least one method | WP_119706148.1 |  | PEM_RS19070 | 4146098 | 4146772 | 1 | glutathione S-transferase family protein |
|  | 4143501 | 4150989 | 7488 | Predicted by at least one method | WP_119706149.1 |  | PEM_RS19075 | 4146867 | 4147475 | 1 | integrase |
|  | 4143501 | 4150989 | 7488 | Predicted by at least one method | WP_119706150.1 |  | PEM_RS19080 | 4147607 | 4147786 | 1 | hypothetical protein |
|  | 4143501 | 4150989 | 7488 | Predicted by at least one method | WP_119706151.1 |  | PEM_RS19085 | 4148327 | 4149199 | 1 | hypothetical protein |
|  | 4143501 | 4150989 | 7488 | Predicted by at least one method | WP_119706152.1 |  | PEM_RS19090 | 4149264 | 4149662 | 1 | hypothetical protein |
|  | 4143501 | 4150989 | 7488 | Predicted by at least one method | WP_119706153.1 |  | PEM_RS19095 | 4149649 | 4149903 | 1 | hypothetical protein |
|  | 4143501 | 4150989 | 7488 | Predicted by at least one method | WP_115875143.1 |  | PEM_RS19100 | 4150084 | 4150989 | -1 | LysR family transcriptional regulator |
| Island 25 | 4152536 | 4157991 | 5455 | Predicted by at least one method | WP_119706165.1 |  | PEM_RS00005 | 1 | 4373402 | 1 | IS3 family transposase |
|  | 4152536 | 4157991 | 5455 | Predicted by at least one method | WP_115875139.1 |  | PEM_RS19110 | 4152536 | 4153015 | 1 | DMT family transporter |
|  | 4152536 | 4157991 | 5455 | Predicted by at least one method | WP_075675695.1 |  | PEM_RS19115 | 4153265 | 4153972 | 1 | AbiV family abortive infection protein |
|  | 4152536 | 4157991 | 5455 | Predicted by at least one method | WP_119706154.1 |  | PEM_RS19120 | 4154047 | 4154835 | -1 | hypothetical protein |
|  | 4152536 | 4157991 | 5455 | Predicted by at least one method | WP_119706155.1 |  | PEM_RS19125 | 4155417 | 4155938 | -1 | hypothetical protein |
|  | 4152536 | 4157991 | 5455 | Predicted by at least one method | WP_119706156.1 |  | PEM_RS19130 | 4156165 | 4157991 | -1 | DUF2813 domain-containing protein |
| Island 26 | 4268713 | 4285443 | 16730 | Predicted by at least one method | WP_119706165.1 |  | PEM_RS00005 | 1 | 4373402 | 1 | IS3 family transposase |
|  | 4268713 | 4285443 | 16730 | Predicted by at least one method | WP_069138079.1 |  | PEM_RS19645 | 4267792 | 4268748 | -1 | cation diffusion facilitator family transporter |
|  | 4268713 | 4285443 | 16730 | Predicted by at least one method | WP_111128876.1 |  | PEM_RS19650 | 4269127 | 4269639 | 1 | phage gp6-like head-tail connector protein |
|  | 4268713 | 4285443 | 16730 | Predicted by at least one method | WP_069138077.1 |  | PEM_RS19655 | 4269636 | 4269986 | 1 | head-tail adaptor protein |
|  | 4268713 | 4285443 | 16730 | Predicted by at least one method | WP_069138076.1 |  | PEM_RS19660 | 4270010 | 4270366 | 1 | DUF3168 domain-containing protein |
|  | 4268713 | 4285443 | 16730 | Predicted by at least one method | WP_069138075.1 |  | PEM_RS19665 | 4270437 | 4271096 | 1 | phage tail protein |
|  | 4268713 | 4285443 | 16730 | Predicted by at least one method | WP_069138074.1 |  | PEM_RS19670 | 4271096 | 4271404 | 1 | hypothetical protein |
|  | 4268713 | 4285443 | 16730 | Predicted by at least one method | WP_069138216.1 |  | PEM_RS19675 | 4271455 | 4271712 | 1 | hypothetical protein |
|  | 4268713 | 4285443 | 16730 | Predicted by at least one method | WP_111128878.1 |  | PEM_RS19680 | 4271788 | 4273149 | 1 | phage tail protein |
|  | 4268713 | 4285443 | 16730 | Predicted by at least one method | WP_069138072.1 |  | PEM_RS19685 | 4273146 | 4273475 | 1 | phage tail protein |
|  | 4268713 | 4285443 | 16730 | Predicted by at least one method | WP_111174903.1 |  | PEM_RS19690 | 4273515 | 4274666 | 1 | hypothetical protein |
|  | 4268713 | 4285443 | 16730 | Predicted by at least one method | WP_111128881.1 |  | PEM_RS19695 | 4274745 | 4275851 | 1 | hypothetical protein |
|  | 4268713 | 4285443 | 16730 | Predicted by at least one method | WP_111128883.1 |  | PEM_RS19700 | 4275853 | 4276305 | 1 | hypothetical protein |
|  | 4268713 | 4285443 | 16730 | Predicted by at least one method | WP_111128884.1 |  | PEM_RS19705 | 4276318 | 4277457 | 1 | hypothetical protein |
|  | 4268713 | 4285443 | 16730 | Predicted by at least one method | WP_111128886.1 |  | PEM_RS19710 | 4277498 | 4278634 | 1 | hypothetical protein |
|  | 4268713 | 4285443 | 16730 | Predicted by at least one method | WP_111128888.1 |  | PEM_RS19715 | 4278786 | 4279481 | 1 | phage minor tail protein L |
|  | 4268713 | 4285443 | 16730 | Predicted by at least one method | WP_111128890.1 |  | PEM_RS19720 | 4279544 | 4280284 | 1 | peptidase P60 |
|  | 4268713 | 4285443 | 16730 | Predicted by at least one method | WP_087921215.1 |  | PEM_RS19725 | 4280277 | 4280879 | 1 | tail assembly protein |
|  | 4268713 | 4285443 | 16730 | Predicted by at least one method | WP_111128892.1 |  | PEM_RS19730 | 4280876 | 4284364 | 1 | host specificity protein J |
|  | 4268713 | 4285443 | 16730 | Predicted by at least one method | WP_069138062.1 |  | PEM_RS19735 | 4284364 | 4284693 | 1 | hypothetical protein |
|  | 4268713 | 4285443 | 16730 | Predicted by at least one method | WP_111128894.1 |  | PEM_RS19740 | 4284693 | 4285403 | 1 | hypothetical protein |
|  | 4268713 | 4285443 | 16730 | Predicted by at least one method |  |  | PEM_RS19745 | 4285429 | 4285614 | -1 | cation diffusion facilitator family transporter |
|  | 4273146 | 4277457 | 4311 | Predicted by at least one method | WP_119706165.1 |  | PEM_RS00005 | 1 | 4373402 | 1 | IS3 family transposase |
|  | 4273146 | 4277457 | 4311 | Predicted by at least one method | WP_111128878.1 |  | PEM_RS19680 | 4271788 | 4273149 | 1 | phage tail protein |
| Isalnd 27 | 4273146 | 4277457 | 4311 | Predicted by at least one method | WP_069138072.1 |  | PEM_RS19685 | 4273146 | 4273475 | 1 | phage tail protein |
|  | 4273146 | 4277457 | 4311 | Predicted by at least one method | WP_111174903.1 |  | PEM_RS19690 | 4273515 | 4274666 | 1 | hypothetical protein |
|  | 4273146 | 4277457 | 4311 | Predicted by at least one method | WP_111128881.1 |  | PEM_RS19695 | 4274745 | 4275851 | 1 | hypothetical protein |
|  | 4273146 | 4277457 | 4311 | Predicted by at least one method | WP_111128883.1 |  | PEM_RS19700 | 4275853 | 4276305 | 1 | hypothetical protein |
|  | 4273146 | 4277457 | 4311 | Predicted by at least one method | WP_111128884.1 |  | PEM_RS19705 | 4276318 | 4277457 | 1 | hypothetical protein |
| Island 28 | 4329054 | 4343066 | 14012 | Predicted by at least one method | WP_119706165.1 |  | PEM_RS00005 | 1 | 4373402 | 1 | IS3 family transposase |
|  | 4329054 | 4343066 | 14012 | Predicted by at least one method | WP_005420550.1 |  | PEM_RS19970 | 4329054 | 4329515 | -1 | hypothetical protein |
|  | 4329054 | 4343066 | 14012 | Predicted by at least one method | WP_004153834.1 |  | PEM_RS19975 | 4329512 | 4329982 | -1 | phage holin family protein |
|  | 4329054 | 4343066 | 14012 | Predicted by at least one method | WP_004153835.1 |  | PEM_RS19980 | 4329991 | 4330356 | -1 | hypothetical protein |
|  | 4329054 | 4343066 | 14012 | Predicted by at least one method | WP_005420544.1 |  | PEM_RS19985 | 4330458 | 4331891 | -1 | Do family serine endopeptidase |
|  | 4329054 | 4343066 | 14012 | Predicted by at least one method | WP_005420542.1 |  | PEM_RS19990 | 4332383 | 4333270 | 1 | hypothetical protein |
|  | 4329054 | 4343066 | 14012 | Predicted by at least one method | WP_111128935.1 |  | PEM_RS19995 | 4333377 | 4334081 | 1 | hypothetical protein |
|  | 4329054 | 4343066 | 14012 | Predicted by at least one method | WP_069138035.1 |  | PEM_RS20000 | 4334097 | 4334456 | -1 | DUF1428 domain-containing protein |
|  | 4329054 | 4343066 | 14012 | Predicted by at least one method | WP_111128937.1 |  | PEM_RS20005 | 4334548 | 4336950 | -1 | S9 family peptidase |
|  | 4329054 | 4343066 | 14012 | Predicted by at least one method | WP_005420534.1 |  | PEM_RS20010 | 4337161 | 4337733 | 1 | Ax21 family protein |
|  | 4329054 | 4343066 | 14012 | Predicted by at least one method | WP_080104364.1 |  | PEM_RS20015 | 4337911 | 4338609 | 1 | DNA-binding response regulator |
|  | 4329054 | 4343066 | 14012 | Predicted by at least one method | WP_005420531.1 |  | PEM_RS20020 | 4338616 | 4339005 | -1 | hypothetical protein |
|  | 4329054 | 4343066 | 14012 | Predicted by at least one method | WP_005420530.1 |  | PEM_RS20025 | 4339184 | 4339882 | 1 | hypothetical protein |
|  | 4329054 | 4343066 | 14012 | Predicted by at least one method | WP_032954666.1 |  | PEM_RS20030 | 4339879 | 4341072 | -1 | MFS transporter |
|  | 4329054 | 4343066 | 14012 | Predicted by at least one method | WP_111128939.1 |  | PEM_RS20035 | 4341170 | 4342057 | 1 | LysR family transcriptional regulator |
|  | 4329054 | 4343066 | 14012 | Predicted by at least one method | WP_005420526.1 |  | PEM_RS20040 | 4342026 | 4342493 | -1 | DUF1456 family protein |
|  | 4329054 | 4343066 | 14012 | Predicted by at least one method | WP_005420525.1 |  | PEM_RS20045 | 4342578 | 4343066 | 1 | hypothetical protein |
| Island 29 | 4369220 | 4373403 | 4183 | Predicted by at least one method | WP_119706165.1 |  | PEM_RS00005 | 1 | 4373402 | 1 | IS3 family transposase |
|  | 4369220 | 4373403 | 4183 | Predicted by at least one method | WP_119706163.1 |  | PEM_RS20160 | 4369454 | 4369777 | -1 | hypothetical protein |
|  | 4369220 | 4373403 | 4183 | Predicted by at least one method | WP_119706164.1 |  | PEM_RS20165 | 4369752 | 4370492 | -1 | hypothetical protein |
|  | 4369220 | 4373403 | 4183 | Predicted by at least one method |  |  | PEM_RS20170 | 4370750 | 4371139 | -1 | hemolysin |
|  | 4369220 | 4373403 | 4183 | Predicted by at least one method |  |  | PEM_RS20175 | 4371136 | 4371500 | -1 | hypothetical protein |
|  | 4369220 | 4373403 | 4183 | Predicted by at least one method |  |  | PEM_RS20180 | 4371601 | 4371929 | -1 | hypothetical protein |
|  | 4369220 | 4373403 | 4183 | Predicted by at least one method | WP_032954795.1 |  | PEM_RS20185 | 4372373 | 4372666 | 1 | transposase |

**Table S3: Annotation of Predicted genes in the Genomic Islands of *Stenotrophomonas* sp. Pemsol**

| Query | Seed Ortholog | evalue | Predicted name | Possible origin for gene transfer | KEGG KO | COG category | Protein Family |
| --- | --- | --- | --- | --- | --- | --- | --- |
| PEM_00073 | 322710.Avin_36080 | 0 |  | *Azotobacter vinelactii* |  | T | Histidine kinase |
| PEM_00075 | 533247.CRD_00627 | 5.5E-41 | Y0750 | *Raphidopsis* |  | S | Protein of unknown function (DUF1524) |
| PEM_00076 | 596151.DesfrDRAFT_0170 | 6.1E-166 | DCM2 | *Desulfovibrio fructosivoran* | K00558 | L | cytosine-specific methyltransferase |
| PEM_00077 | 596151.DesfrDRAFT_0169 | 2E-60 |  | *Desulfovibrio fructosivoran* |  |  |  |
| PEM_00081 | 190486.XAC2246 | 1.3E-64 |  | *Xanthomonas axonopodis citris* |  | S | Protein of unknown function (DUF1629) |
| PEM_00082 | 190486.XAC3322 | 4E-66 |  | *Xanthomonas axonopodis citris* |  | S | Protein of unknown function (DUF1629) |
| PEM_00083 | 190486.XAC2246 | 1.6E-36 |  | *Xanthomonas axonopodis citris* |  | S | Protein of unknown function (DUF1629) |
| PEM_00084 | 316273.XCV2688 | 3.3E-63 |  | *Xanthomoas campestris vesicatoria* |  | S | Protein of unknown function (DUF1629) |
| PEM_00085 | 190486.XAC2862 | 4.1E-63 |  | *Xanthomonas axonopodis citris* |  | S | Protein of unknown function (DUF1629) |
| PEM_00086 | 316273.XCV2685 | 3.5E-86 |  | *Xanthomoas campestris vesicatoria* |  | S | Protein of unknown function (DUF1629) |
| PEM_00087 | 316273.XCV2684 | 8.8E-213 |  | *Xanthomoas campestris vesicatoria* |  |  |  |
| PEM_00202 | 522373.Smlt0617 | 7.4E-215 | METB | *Stenotrophomonas maltophilia K279a* | K01739,K01758, K01760,K17217 | E | Cystathionine gamma-synthase |
| PEM_00203 | 1045855.DSC_02425 | 2.2E-80 | WZM | *Pseudoxanthomonas spadix* | K01992,K09690 | V | transporter |
| PEM_00204 | 228410.NE0483 | 7.7E-128 | TAGH | *Nitrosomonas europaea* | K09689,K09691,K09693 | P | ABC, transporter |
| PEM_00205 | 223283.PSPTO_1074 | 9E-118 |  | *Pseudomonas syringae pv. tomato* | K07011 | M | Glycosyl transferase, family 2 |
| PEM_00206 | 640510.BC1001_0680 | 6.8E-89 |  | *Burkholderia sp. CCGE1001* | K07011 | M | Glycosyl transferase, family 2 |
| PEM_00207 | 452637.Oter_3973 | 1.8E-177 |  | *Opitutus terrae (strain DSM 11246 / JCM 15787 / PB90-1)* |  | M | Glycosyl transferase family 2 |
| PEM_00208 | 522373.Smlt0784 | 3.7E-127 |  | *Stenotrophomonas maltophilia (strain K279a)* |  | M | Glycosyl Transferase |
| PEM_00209 | 391008.Smal_0635 | 9.5E-176 |  | *Stenotrophomonas maltophilia (strain R551-3)* |  |  |  |
| PEM_00288 | 426355.Mrad2831_3035 | 2.4E-22 |  | *Methylobacterium radiotolerans (strain ATCC 27329 / DSM 1819 / JCM 2831)* |  |  |  |
| PEM_00290 | 266835.mlr4370 | 0.00000039 |  | *Rhizobium loti (strain MAFF303099) (Mesorhizobium loti)* |  |  |  |
| PEM_00581 | 522373.Smlt1010 | 5.4E-70 |  | *Stenotrophomonas maltophilia (strain K279a)* |  | S | Acetyltransferase (GNAT) family |
| PEM_00582 | 216591.BCAM1200 | 3.9E-14 |  | *Burkholderia cenocepacia* |  | S | Inner membrane protein YmfA |
| PEM_00583 | 522373.Smlt0064 | 2.4E-141 | ELI_1277 | *Stenotrophomonas maltophilia (strain K279a)* | K06919 | L | prophage primase |
| PEM_00585 | 190485.XCC3461 | 2.4E-147 |  | *Xanthomonas campestris pv. campestris* | K16214 | S | zeta toxin |
| PEM_00868 | 522373.Smlt1390 | 0 | FHAB | *Stenotrophomonas maltophilia (strain K279a)* | K15125 | U | filamentous hemagglutinin |
| PEM_00869 | 522373.Smlt1396A | 2.4E-12 |  | *Stenotrophomonas maltophilia (strain K279a)* |  | S | Protein of unknown function, DUF596 |
| PEM_00870 | 391008.Smal_3995 | 1.3E-11 |  | *Stenotrophomonas maltophilia (strain R551-3)* |  | S | Protein of unknown function (DUF2628) |
| PEM_01042 | 397945.Aave_0535 | 8.1E-44 |  | *Acidovorax avenae subsp. citrulli* |  | T | MASE1 |
| PEM_01043 | 397945.Aave_0536 | 1.7E-19 |  | *Acidovorax avenae subsp. citrulli* |  |  |  |
| PEM_01044 | 397945.Aave_0535 | 2.4E-43 |  | *Acidovorax avenae subsp. citrulli* |  | T | MASE1 |
| PEM_01288 | 441620.Mpop_2731 | 1E-60 | DCM | *Methylobacterium populi* | K00558 | L | C-5 cytosine-specific DNA methylase |
| PEM_01295 | 522373.Smlt1978 | 1.1E-100 |  | *Stenotrophomonas maltophilia (strain K279a)* |  | S | conserved protein |
| PEM_01296 | 522373.Smlt1978 | 1.7E-48 |  | *Stenotrophomonas maltophilia (strain K279a)* |  | S | conserved protein |
| PEM_01297 | 1127134.NOCYR_5259 | 3.8E-09 |  | *Nocardia cyriacigeorgica (strain GUH-2)* |  | K | Helix-turn-helix |
| PEM_01300 | 56780.SYN_01916 | 4.5E-135 |  | *Syntrophus aciditrophicus (strain SB)* |  | L | integrase family |
| PEM_01654 | 522373.Smlt2386 | 1.9E-76 | BMUL_5091 | *Stenotrophomonas maltophilia (strain K279a)* |  | K | Gcn5-related n-acetyltransferase |
| PEM_01658 | 216591.BCAM1881 | 4.8E-81 |  | *Burkholderia cenocepacia* |  |  |  |
| PEM_01659 | 216591.BCAM1882 | 2.9E-48 |  | *Burkholderia cenocepacia* |  |  |  |
| PEM_01661 | 522373.Smlt2497 | 6.1E-130 |  | *Stenotrophomonas maltophilia (strain K279a)* |  |  |  |
| PEM_01666 | 522373.Smlt2501 | 3.4E-102 |  | *Stenotrophomonas maltophilia (strain K279a)* | K07141 | S | 2-C-methyl-D-erythritol 4-phosphate cytidylyltransferase |
| PEM_01667 | 391008.Smal_1990 | 8.1E-181 |  | *Stenotrophomonas maltophilia (strain R551-3)* | K07402 | O | Xanthine dehydrogenase accessory factor |
| PEM_01690 | 522373.Smlt2534 | 2.8E-122 |  | *Stenotrophomonas maltophilia (strain K279a*) |  | S | Protein of unknown function DUF72 |
| PEM_01691 | 522373.Smlt2535 | 2.3E-71 |  | *Stenotrophomonas maltophilia (strain K279a)* |  | S | BLUF |
| PEM_01693 | 522373.Smlt2711 | 8.2E-11 | OCAR_6262 | *Stenotrophomonas maltophilia (strain K279a)* |  | T | response regulator |
| PEM_01694 | 391008.Smal_1741 | 1.8E-30 |  | *Stenotrophomonas maltophilia (strain R551-3)* |  | S | domain protein |
| PEM_01695 | 522373.Smlt2543 | 3.6E-83 |  | *Stenotrophomonas maltophilia (strain K279a)* |  | M | Glycosyl transferase |
| PEM_01696 | 522373.Smlt2544 | 2.1E-106 |  | *Stenotrophomonas maltophilia (strain K279a)* |  | S | Nodulation protein S (NodS) |
| PEM_01697 | 522373.Smlt2545 | 4.2E-131 |  | *Stenotrophomonas maltophilia (strain K279a)* |  | S | GlcNAc-PI de-N-acetylase |
| PEM_01698 | 1045855.DSC_05565 | 2.2E-68 |  | [*Pseudoxanthomonas spadix (strain BD-a59)*](http://www.uniprot.org/taxonomy/1045855) |  | S | acyl-CoA dehydrogenase |
| PEM_01699 | 522373.Smlt2547 | 3.5E-70 | BMUL_5515 | *Stenotrophomonas maltophilia (strain K279a)* |  | S | Inherit from bactNOG: Diguanylate cyclase phosphodiesterase |
| PEM_01700 | 522373.Smlt2548 | 5.7E-220 | YBDR | *Stenotrophomonas maltophilia (strain K279a)* | K00098 | C | Dehydrogenase |
| PEM_01702 | 314565.XC_3765 | 2.5E-76 |  | *Xanthomonas campestris pv. campestris (strain 8004)* |  |  |  |
| PEM_01703 | 522373.Smlt2552 | 5E-81 |  | *Stenotrophomonas maltophilia (strain K279a)* |  | Q | Isochorismatase family |
| PEM_01704 | 522373.Smlt2553 | 2E-39 |  | *Stenotrophomonas maltophilia (strain K279a)* |  |  |  |
| PEM_01705 | 522373.Smlt2554 | 4.8E-58 |  | *Stenotrophomonas maltophilia (strain K279a)* |  |  |  |
| PEM_01706 | 742159.HMPREF0004_4690 | 8.4E-70 |  | *Achromobacter piechaudii ATCC 43553* |  |  |  |
| PEM_01707 | 391008.Smal_2029 | 1.3E-164 | PTXS | *Stenotrophomonas maltophilia (strain R551-3)* | K02525, K02529, K03435 | K | Transcriptional regulator |
| PEM_01806 | 743721.Psesu_2031 | 3.1E-30 | ECPD | *Pseudoxanthomonas suwonensis (strain 11-1)* | K07346, K07357, K15540 | N, U | Pfam:Pili_assembly_C |
| PEM_01808 | 1097668.BYI23_B003340 | 2.2E-155 | HTRE | *Burkholderia sp. YI23* | K07347 | M | outer membrane usher protein |
| PEM_01809 | 340100.Bpet4466 | 1.9E-32 | ECPD | *Bordetella petrii (strain ATCC BAA-461 / DSM 12804 / CCUG 43448)* | K07346, K07357 K15540 | O | Pili assembly chaperone |
| PEM_01810 | 626418.bglu_1g22890 | 0.000000023 | FIMA | *Burkholderia glumae (strain BGR1)* | K07345, K12517 | N, U | Inherit from proNOG: Fimbrial protein |
| PEM_01811 | 522373.Smlt0732 | 1.6E-10 | YEHC | *Stenotrophomonas maltophilia (strain K279a)* | K07346, K15540 | N, U | Chaperone |
| PEM_01814 | 350701.BDAG_03369 | 5.5E-33 |  | *Burkholderia dolosa AU0158* | K15540 | O | Pili assembly chaperone |
| PEM_01816 | 667129.HMPREF0758_4622 | 1.1E-157 | YEHB | *Serratia odorifera DSM 4582* | K07347 | M | outer membrane usher protein |
| PEM_01817 | 340100.Bpet4466 | 6.6E-44 | ECPD | *Bordetella petrii (strain ATCC BAA-461 / DSM 12804 / CCUG 43448)* | K07346, K07357, K15540 | O | Pili assembly chaperone |
| PEM_01818 | 266265.Bxe_C1153 | 3.9E-09 | FIMA | *Bordetella petrii (strain ATCC BAA-461 / DSM 12804 / CCUG 43448)* | K07345 | N, U | Fimbrial |
| PEM_01819 | 716541.ECL_04444 | 4.1E-61 |  | *Enterobacter cloacae subsp. cloacae (strain ATCC 13047 / DSM 30054 / NBRC 13535 / NCDC 279-56)* |  | M | Inherit from proNOG: opacity protein and |
| PEM_02561 | 522373.Smlt2892 | 2.2E-195 | BMUL_3317 | *Stenotrophomonas maltophilia (strain K279a)* | K08224 | G | Major Facilitator |
| PEM_02562 | 522373.Smlt2893 | 2.2E-166 |  | *Stenotrophomonas maltophilia (strain K279a)* |  | K | LysR family transcriptional regulator |
| PEM_02563 | 391008.Smal_2344 | 1E-266 |  | *Stenotrophomonas maltophilia* (R551-3) |  | S | amidohydrolase |
| PEM_02564 | 522373.Smlt2895 | 8.4E-118 | OCAR_5703 | *Stenotrophomonas maltophilia (strain K279a)* | K06867 | F, J | Ankyrin repeat-containing protein |
| PEM_02565 | 522373.Smlt2896 | 1.3E-160 |  | *Stenotrophomonas maltophilia (strain K279a)* | K19242 | K | lysr family transcriptional regulator |
| PEM_02566 | 391008.Smal_2347 | 9.3E-48 |  | *Stenotrophomonas maltophilia* (R551-3) |  |  |  |
| PEM_02567 | 391008.Smal_2347 | 3.3E-72 |  | *Stenotrophomonas maltophilia* (R551-3) |  |  |  |
| PEM_02568 | 522373.Smlt2898 | 1E-213 | YAHJ | *Stenotrophomonas maltophilia (strain K279a)* | K01485 | F | deaminase |
| PEM_02569 | 522373.Smlt2899 | 5.9E-110 |  | *Stenotrophomonas maltophilia (strain K279a)* |  |  |  |
| PEM_02680 | 391008.Smal_2251 | 0 |  | *Stenotrophomonas maltophilia* (R551-3) |  | M | rhs family |
| PEM_02689 | 391008.Smal_1583 | 1.4E-200 | MCHE | *Stenotrophomonas maltophilia* (R551-3) | K02022,K13408 | U | secretion protein |
| PEM_02690 | 391008.Smal_1584 | 0 | CVAB | *Stenotrophomonas maltophilia* (R551-3) | K06147,K13409 | V | AtP-binding protein |
| PEM_02692 | 190486.XAC3298 | 4.5E-105 |  | *Xanthomonas axonopodis pv. citri (strain 306)* |  | L | Integrase |
| PEM_02741 | 391008.Smal_3245 | 0 |  | *Stenotrophomonas maltophilia* (R551-3) | K19232 | U, W | Pfam:HIM |
| PEM_03048 | 522373.Smlt4157 | 2.6E-66 | OCAR_5815 | *Stenotrophomonas maltophilia (strain K279a)* | K03746 | S | Histone family protein nucleoid-structuring protein H-NS |
| PEM_03049 | 391008.Smal_3563 | 1.3E-62 | YQJF | *Stenotrophomonas maltophilia (strain K279a)* | K15977 | S | Inner membrane protein YqjF |
| PEM_03050 | 522373.Smlt4159 | 7.4e-309 | PBP-2 | *Stenotrophomonas maltophilia (strain K279a)* |  | V | Beta-lactamase |
| PEM_03054 | 522373.Smlt0332 | 1.5E-177 | SCLAV_0044 | *Stenotrophomonas maltophilia (strain K279a)* | K11904 | S | Rhs element vgr protein |
| PEM_03055 | 522373.Smlt0324 | 1E-161 | Q | *Stenotrophomonas maltophilia (strain K279a)* |  | S | portal protein |
| PEM_03244 | 391008.Smal_2784 | 5.4E-39 |  | *Stenotrophomonas maltophilia* (R551-3) |  |  |  |
| PEM_03245 | 391008.Smal_2785 | 1.3E-35 | BMUL_3731 | *Stenotrophomonas maltophilia* (R551-3) |  | S | Protein of unknown function (DUF1653) |
| PEM_03246 | 391008.Smal_2787 | 6.7E-49 | EMRE | *Stenotrophomonas maltophilia* (R551-3) | K03297 | P | multidrug resistance protein |
| PEM_03248 | 391008.Smal_2789 | 2E-105 | RHTB | *Stenotrophomonas maltophilia* (R551-3) |  | E | Homoserine homoserine lactone efflux protein |
| PEM_03249 | 391008.Smal_2790 | 6.8E-108 | CLPP | *Stenotrophomonas maltophilia* (R551-3) | K01358 | O | Cleaves peptides in various proteins in a process that requires ATP hydrolysis. Has a chymotrypsin-like activity. Plays a major role in the degradation of misfolded proteins (By similarity) |
| PEM_03250 | 391008.Smal_2791 | 1.2E-53 |  | *Stenotrophomonas maltophilia* (R551-3) |  |  |  |
| PEM_03251 | 391008.Smal_2792 | 9E-48 |  | *Stenotrophomonas maltophilia* (R551-3) |  | K | Transcriptional regulator |
| PEM_03252 | 522373.Smlt3366 | 3.2E-109 | YGEA | *Stenotrophomonas maltophilia* (strain K279a) | K01779 | E | aspartate racemase |
| PEM_03254 | 391008.Smal_2795 | 4.5E-84 |  | *Stenotrophomonas maltophilia* (R551-3) |  | S | Protein of unknown function (DUF3011) |
| PEM_03255 | 391008.Smal_2797 | 0.00000012 |  | *Stenotrophomonas maltophilia* (R551-3) |  | S | acetyltransferase, GNAT family |
| PEM_03256 | 1001585.MDS_1719 | 3.7E-42 |  | *Pseudomonas mendocina* (strain NK-01) | K01104 | T | LMWPc |
| PEM_03257 | 384676.PSEEN3596 | 2.5E-11 |  | *Pseudomonas entomophila* (strain L48) |  |  |  |
| PEM_03258 | 390235.PputW619_3104 | 5.4E-57 | OCAR_5492 | *Pseudomonas putida* (strain W619) |  | S | Antioxidant protein with alkyl hydroperoxidase activity. Required for the reduction of the AhpC active site cysteine residues and for the regeneration of the AhpC enzyme activity (By similarity) |
| PEM_03259 | 395492.Rleg2_1661 | 6.7E-44 |  | *Rhizobium leguminosarum* *bv. trifolii* (strain WSM2304) |  | J | endoribonuclease L-PSP |
| PEM_03261 | 743721.Psesu_1124 | 1.6E-56 | INTE | *Pseudoxanthomonas suwonensis* (strain 11-1*)* |  | L | Integrase |
| PEM_03263 | 391008.Smal_2798 | 4.8E-61 | MERR |  |  | K | Transcriptional Regulator, MerR family |
| PEM_03264 | 391008.Smal_2799 | 3.9E-48 | IHFA | *Stenotrophomonas maltophilia* (strain R551-3) | K04764,K05788 | K | This protein is one of the two subunits of integration host factor, a specific DNA-binding protein that functions in genetic recombination as well as in transcriptional and translational control (By similarity) |
| PEM_03265 | 391008.Smal_2800 | 0 | PHET | *Stenotrophomonas maltophilia* (strain R551-3) | K01890 | J | phenylalanyl-tRNA synthetase beta subunit |
| PEM_03266 | 522373.Smlt3374 | 3.4E-189 | PHES | *Stenotrophomonas maltophilia* (strain K279a*)* | K01889 | J | Phenylalanyl-tRNA synthetase alpha subunit |
| PEM_03381 | 522373.Smlt4372 | 1.8E-78 | ARR | *Stenotrophomonas maltophilia* (strain K279a*)* | K19062 | M | rifampin ADP-ribosyl transferase |
| PEM_03382 | 522373.Smlt4373 | 2.4E-73 |  | *Stenotrophomonas maltophilia* (strain K279a*)* |  |  |  |
| PEM_03385 | 76114.ebA2464 | 7.1E-54 |  | *Aromatoleum aromaticum* (strain EbN1) (*Azoarcus* sp. (strain EbN1)) |  |  |  |
| PEM_03390 | 1045855.DSC_14975 | 3.6E-48 |  | *Pseudoxanthomonas spadix* (strain BD-a59) |  | M | Inherit from COG: YD repeat protein |
| PEM_03427 | 522373.Smlt4430 | 0 |  | *Stenotrophomonas maltophilia* (strain K279a) |  | M | rhs family |
| PEM_03658 | 522373.Smlt4693 | 0 | YIDC | *Stenotrophomonas maltophilia* (strain K279a) | K03217 | U | Required for the insertion and or proper folding and or complex formation of integral membrane proteins into the membrane. Involved in integration of membrane proteins that insert both dependently and independently of the Sec translocase complex, as well as at least some lipoproteins. Aids folding of multispanning membrane proteins (By similarity) |
| PEM_03659 | 522373.Smlt4694 | 5.6E-84 | RNPA | *Stenotrophomonas maltophilia* (strain K279a) | K03536 | J | RNaseP catalyzes the removal of the 5'-leader sequence from pre-tRNA to produce the mature 5'-terminus. It can also cleave other RNA substrates such as 4.5S RNA. The protein component plays an auxiliary but essential role in vivo by binding to the 5'-leader sequence and broadening the substrate specificity of the ribozyme (By similarity) |
| PEM_03660 | 522373.Smlt4695 | 5.3E-16 | RPMH | *Stenotrophomonas maltophilia* (strain K279a) | K02914 | J | 50S ribosomal protein L34 |
| PEM_03661 | 522373.Smlt0001 | 8.7E-249 | DNAA | *Stenotrophomonas maltophilia* (strain K279a) | K02313 | L | it binds specifically double-stranded DNA at a 9 bp consensus (dnaA box) 5'-TTATC CA A CA A-3'. DnaA binds to ATP and to acidic phospholipids (By similarity) |
| PEM_03662 | 522373.Smlt0002 | 2.4E-196 | DNAN | *Stenotrophomonas maltophilia* (strain K279a) | K02338 | L | DNA polymerase III is a complex, multichain enzyme responsible for most of the replicative synthesis in bacteria. This DNA polymerase also exhibits 3' to 5' exonuclease activity. The beta chain is required for initiation of replication once it is clamped onto DNA, it slides freely (bidirectional and ATP- independent) along duplex DNA (By similarity) |
| PEM_03663 | 522373.Smlt0004 | 6.6E-202 | RECF | *Stenotrophomonas maltophilia* (strain K279a) | K03629 | L | it is required for DNA replication and normal SOS inducibility. RecF binds preferentially to single-stranded, linear DNA. It also seems to bind ATP (By similarity) |
| PEM_03664 | 522373.Smlt0005 | 0 | GYRB | *Stenotrophomonas maltophilia* (strain K279a) | K02470 | L | DNA gyrase negatively supercoils closed circular double- stranded DNA in an ATP-dependent manner and also catalyzes the interconversion of other topological isomers of double-stranded DNA rings, including catenanes and knotted rings (By similarity) |
| PEM_03665 | 391008.Smal_0005 | 5.7E-129 |  | *Stenotrophomonas maltophilia* (strain R551-3) | K07052 | S | CAAX amino terminal protease family |
| PEM_03666 | 391008.Smal_0006 | 7.6E-130 |  | *Stenotrophomonas maltophilia* (strain R551-3) |  | O | peptidase M48, Ste24p |
| PEM_03667 | 522373.Smlt0008 | 3E-200 | ORF398 | *Stenotrophomonas maltophilia* (strain K279a) |  | S | domain protein |
| PEM_03668 | 391008.Smal_0008 | 3.4E-108 |  | *Stenotrophomonas maltophilia* (strain R551-3) | K03832 | M | Pfam:TonB |
| PEM_03770 | 522373.Smlt0157 | 0 |  | *Stenotrophomonas maltophilia* (strain K279a) | K02014 | P | TonB-dependent receptor |
| PEM_03771 | 500635.MITSMUL_03861 | 1.4E-31 |  | *Mitsuokella multacida* DSM 20544 |  | S | phosphoglycerate mutase family protein |
| PEM_03773 | 391008.Smal_3568 | 2.7E-14 | AVA_1330 | *Stenotrophomonas maltophilia* (strain R551-3) | K07497 | L | Integrase core domain |
| PEM_03776 | 391612.CY0110_22834 | 0.000000046 | BMUL_4716 | *Cyanothece sp.* CCY0110 |  | S | aaa ATPase |
| PEM_03777 | 402626.Rpic_4575 | 0.00000089 |  | *Ralstonia pickettii* (strain 12J) |  |  |  |
| PEM_03778 | 1045855.DSC_02605 | 3.5E-22 |  | *Pseudoxanthomonas spadix* (strain BD-a59) | K03746 | S | nucleoid-structuring protein |
| PEM_03779 | 318167.Sfri_2854 | 3.8E-80 |  | *Shewanella frigidimarina* (strain NCIMB 400) |  |  |  |
| PEM_03780 | 589865.DaAHT2_1056 | 1.4E-242 |  | *Desulfurivibrio alkaliphilus (strain DSM 19089 / UNIQEM U267 / AHT2)* | K02168 | P | Choline carnitine betaine transporter |
| PEM_03781 | 709797.CSIRO_3742 | 4.7E-92 |  | *Bradyrhizobiaceae bacterium* SG-6C |  | S | Predicted membrane protein (DUF2254) |
| PEM_03782 | 522373.Smlt0115 | 5.2E-41 |  | *Stenotrophomonas maltophilia* (strain K279a) |  | V | Beta-lactamase |
| PEM_03783 | 543728.Vapar_2066 | 1.1E-27 |  | *Variovorax paradoxus* (strain S110) | K02014 | P | receptor |
| PEM_03784 | 1045855.DSC_06700 | 1.7E-79 |  | *Pseudoxanthomonas spadix* (strain BD-a59) | K07090 | S | Sulfite exporter TauE/SafE |
| PEM_03785 | 316273.XCV2442 | 7.7E-37 |  | *Xanthomonas campestris pv. vesicatoria* (strain 85-10) |  | S | Putative phosphatase (DUF442) |
| PEM_03786 | 316273.XCV2443 | 8.4E-52 |  | *Xanthomonas campestris pv. vesicatoria* (strain 85-10) | K07112 | S | YeeE YedE family protein |
| PEM_03787 | 316273.XCV2445 | 2.7E-135 |  | *Xanthomonas campestris pv. vesicatoria* (strain 85-10) | K01069 | P | beta-lactamase domain protein |
| PEM_03789 | 690566.Sphch_3377 | 7.1E-223 |  | *Sphingobium chlorophenolicum* L-1 | K02014,K16089 | P | TonB-dependent Receptor Plug |
| PEM_03790 | 272630.MexAM1_META1p2602 | 1.6E-36 | HMRR | *Methylobacterium extorquens* (strain ATCC 14718 / DSM 1338 / JCM 2805 / NCIMB 9133 / AM1) |  | K | MerR family transcriptional regulator |
| PEM_03791 | 314266.SKA58_04115 | 1.2E-51 |  | *Sphingomonas* sp. (strain SKA58) |  | P | Cation efflux protein |
| PEM_03792 | 395492.Rleg2_1661 | 3.3E-43 |  | *Rhizobium leguminosarum bv. trifolii* (strain WSM2304) |  | J | endoribonuclease L-PSP |
| PEM_03793 | 398578.Daci_5638 | 7.9E-121 |  | *Delftia acidovorans* (strain DSM 14801 / SPH-1) |  | K | transcriptional Regulator LysR family |
| PEM_03794 | 339670.Bamb_3531 | 2.2E-111 |  | *Burkholderia ambifaria* (strain ATCC BAA-244 / AMMD) (*Burkholderia cepacia* (strain AMMD)) |  | S | Short-chain dehydrogenase reductase sdr |
| PEM_03795 | 267608.RSc2594 | 3.2E-74 | GSTCH1 | *Ralstonia solanacearum* (strain GMI1000) (*Pseudomonas solanacearum*) | K00799, K03599 | O | Glutathione S-transferase, C-terminal domain |
| PEM_03796 | 640512.BC1003_0658 | 2.2E-74 | LDB1079 | *Burkholderia* sp. (strain CCGE1003) |  | L | integrase family |
| PEM_03800 | 517433.PanABDRAFT_0490 | 3.9E-99 | LYSR18 | *Pantoea* sp. aB | K18900 | K | lysr family transcriptional regulator |
| PEM_03801 | 682634.SOD_a07460 | 8.7E-191 | SCLAV_5667 | *Serratia plymuthica* 4Rx13 |  | F | amidohydrolase |
| PEM_03802 | 1001585.MDS_3582 | 3.9E-42 |  | *Pseudomonas mendocina* (strain NK-01) | K09936 | S | Protein of unknown function, DUF606 |
| PEM_03803 | 78245.Xaut_0790 | 2.4E-32 |  | *Xanthobacter autotrophicus* (strain ATCC BAA-1158 / Py2) |  |  |  |
| PEM_03804 | 572477.Alvin_2808 | 2.8E-80 |  | *Allochromatium vinosum* (strain ATCC 17899 / DSM 180 / NBRC 103801 / NCIMB 10441 / D) (*Chromatium vinosum*) |  |  |  |
| PEM_03806 | 384676.PSEEN3569 | 1.2E-243 |  | *Pseudomonas entomophila* (strain L48) |  | S | Inherit from proNOG: ATP-dependent OLD family endonuclease |
| PEM_03807 | 391008.Smal_0122 | 4.4E-195 | GLNL | *Stenotrophomonas maltophilia* (strain R551-3) | K02482, K07708, K07710, K13532, K13533 | T | Signal transduction histidine kinase, nitrogen specific |
| PEM_03808 | 522373.Smlt0159 | 2.5E-257 | NTRC | *Stenotrophomonas maltophilia* (strain K279a) | K07712, K07714 | T | two components, sigma54 specific, transcriptional regulator, Fis family |
| PEM_03809 | 522373.Smlt0160 | 2.3E-97 | SODC | *Stenotrophomonas maltophilia* (strain K279a) | K04565 | P | Destroys radicals which are normally produced within the cells and which are toxic to biological systems (By similarity) |
| PEM_03810 | 522373.Smlt0161 | 7.8E-107 | SODC | *Stenotrophomonas maltophilia* (strain K279a) | K04565 | P | Destroys radicals which are normally produced within the cells and which are toxic to biological systems (By similarity) |
| PEM_03811 | 522373.Smlt0163 | 4.9E-163 |  | *Stenotrophomonas maltophilia* (strain K279a) |  |  |  |
| PEM_03812 | 522373.Smlt0164 | 1E-241 | FADA2 |  | K00626, K00632 | I | Catalyzes the final step of fatty acid oxidation in which acetyl-CoA is released and the CoA ester of a fatty acid two carbons shorter is formed (By similarity) |
| PEM_03813 | 391008.Smal_0129 | 1.8E-227 | HEMY | *Stenotrophomonas maltophilia* (strain R551-3) | K02498 | H | HemY protein |
| PEM_03814 | 383407.Xoryp_010100001530 | 2.9E-68 | HEMX | *Xanthomonas oryzae pv. oryzicola* (strain BLS256) | K02496, K13543 | H | c-methyltransferase |
| PEM_03815 | 522373.Smlt0167 | 1.4E-119 | HEMD | *Stenotrophomonas maltophilia (strain K279a)* | K01719, K13542,K13543 | H | synthase |
| PEM_03816 | 522373.Smlt0168 | 6.1E-80 |  | *Stenotrophomonas maltophilia (strain K279a)* |  | S | Putative thioesterase (yiiD_Cterm) |
| PEM_03817 | 314565.XC_0211 | 1.3E-28 |  | Xanthomonas campestris pv. campestris (strain 8004) |  |  |  |
| PEM_03818 | 522373.Smlt0170 | 3.9E-64 | YIBN | *Stenotrophomonas maltophilia* (strain K279a) | K01011 | P | Rhodanese domain protein |
| PEM_03819 | 522373.Smlt0171 | 2.2E-91 | SECB | *Stenotrophomonas maltophilia* (strain K279a) | K03071 | U | One of the proteins required for the normal export of preproteins out of the cell cytoplasm. It is a molecular chaperone that binds to a subset of precursor proteins, maintaining them in a translocation-competent state. It also specifically binds to its receptor SecA (By similarity) |

Number of Predicted genomic island in Pemsol = 29

Total number of Associated genes = 227

**Table S4: Annotation of the identified unique genes in Pemsol**

| Query | Seed Ortholog | evalue | Predicted name | Possible origin | KEGG KO | COG Cat | eggnog HMM Desc |
| --- | --- | --- | --- | --- | --- | --- | --- |
| unique/209/9/Org9_Gene218 | 83406.HDN1F_01080 | 1.30E-71 |  | gamma proteobacterium HdN1 |  | M | Glycosyl Transferase |
| unique/222/9/Org9_Gene548 | 314565.XC_0701 | 2.40E-147 |  | *Xanthomonas campestris pv. campestris* (strain 8004) | K16214 | S | zeta toxin |
| unique/284/9/Org9_Gene1720 | 99287.STM3478 | 1.10E-42 | BIGA | *Salmonella typhimurium* (strain LT2 / SGSC1412 / ATCC 700720) | K12516 | S | surface-exposed virulence protein |
| unique/357/9/Org9_Gene3664 | 391008.Smal_3568 | 2.70E-14 | AVA_1330 | *Stenotrophomonas maltophilia* (strain R551-3) | K07497 | L | Integrase core domain |
| unique/418/9/Org9_Gene198 | 452637.Oter_3973 | 1.80E-177 |  | *Opitutus terrae* (strain DSM 11246 / JCM 15787 / PB90-1) |  | M | Glycosyl transferase family 2 |
| unique/481/9/Org9_Gene197 | 640510.BC1001_0680 | 6.80E-89 |  | *Burkholderia sp*. CCGE1001 | K07011 | M | Glycosyl transferase, family 2 |
| unique/484/9/Org9_Gene196 | 223283.PSPTO_1074 | 9.00E-118 |  | *Pseudomonas syringae pv. tomato* (strain ATCC BAA-871 / DC3000) | K07011 | M | Glycosyl transferase, family 2 |
| unique/493/9/Org9_Gene67 | 322710.Avin_36080 | 0 |  | *Azotobacter vinelandii* (strain DJ / ATCC BAA-1303) |  | T | Histidine kinase |
| unique/521/9/Org9_Gene2810 | 266265.Bxe_C1143 | 1.10E-195 | CVAB | *Paraburkholderia xenovorans* (strain LB400) | K13409 | V | Colicin V processing peptidase |
| unique/665/9/Org9_Gene2864 | 216591.BCAM0224 | 1.20E-57 |  | *Burkholderia cenocepacia* (strain ATCC BAA-245 / DSM 16553 / LMG 16656 / NCTC 13227 / J2315 / CF5610) (Burkholderia cepacia (strain J2315)) |  | U, W | Pfam: YadA |
| unique/765/9/Org9_Gene3697 | 384676.PSEEN3569 | 1.20E-243 |  | *Pseudomonas entomophila* (strain L48) |  | S | Inherit from proNOG: ATP-dependent OLD family endonuclease |
| unique/841/9/Org9_Gene69 | 533247.CRD_00627 | 5.50E-41 | Y0750 | *Raphidiopsis brookii* D9 |  | S | Protein of unknown function (DUF1524) |
| unique/855/9/Org9_Gene3314 | 190485.XCC0449 | 3.00E-201 |  | *Xanthomonas campestris pv. campestris* (strain ATCC 33913 / DSM 3586 / NCPPB 528 / LMG 568 / P 25) | K03791 | O | Peptidase family M23 |
| unique/1032/9/Org9_Gene2560 | 383407.Xoryp_010100016550 | 2.80E-214 |  | *Xanthomonas oryzae pv. oryzicola* (strain BLS256) |  | M | Glycosyl transferase family protein |
| unique/1121/9/Org9_Gene70 | 596151.DesfrDRAFT_0170 | 6.10E-166 | DCM2 | *Desulfovibrio fructosivorans* JJ | K00558 | L | cytosine-specific methyltransferase |
| unique/1165/9/Org9_Gene1323 | 580332.Slit_1205 | 1.60E-86 |  | *Sideroxydans lithotrophicus* (strain ES-1) |  |  |  |
| unique/1172/9/Org9_Gene3255 | 31964.CMS_0032 | 4.80E-14 |  | *Clavibacter michiganensis subsp. sepedonicus* (strain ATCC 33113 / DSM 20744 / JCM 9667 / LMG 2889 / C-1) (*Corynebacterium sepedonicum*) |  |  |  |
| unique/1185/9/Org9_Gene2616 | 190486.XAC3298 | 4.50E-105 |  | *Xanthomonas axonopodis pv. citri* (strain 306) |  | L | Integrase |
| unique/1334/9/Org9_Gene3692 | 682634.SOD_a07460 | 8.70E-191 | SCLAV_5667 | *Serratia plymuthica* 4Rx13 |  | F | amidohydrolase |
| unique/1356/9/Org9_Gene1739 | 878320.AcavA_010100019329 | 7.40E-41 |  | *Aeromonas caviae* (*Aeromonas punctata*) |  | L | mutt nudix family protein |
| unique/1388/9/Org9_Gene3695 | 572477.Alvin_2808 | 2.80E-80 |  | *Allochromatium vinosum* (strain ATCC 17899 / DSM 180 / NBRC 103801 / NCIMB 10441 / D) (*Chromatium vinosum*) |  |  |  |
| unique/1447/9/Org9_Gene3668 | 402626.Rpic_4575 | 8.90E-07 |  | *Ralstonia pickettii* (strain 12J) |  |  |  |
| unique/1466/9/Org9_Gene2227 | 1045856.EcWSU1_01282 | 1.60E-69 |  | *Enterobacter cloacae* EcWSU1 |  |  |  |
| unique/1476/9/Org9_Gene195 | 228410.NE0483 | 7.70E-128 | TAGH | *Nitrosomonas europaea* (strain ATCC 19718 / CIP 103999 / KCTC 2705 / NBRC 14298) | K09689,K09691,K09693 | P | ABC, transporter |
| unique/1613/9/Org9_Gene211 | 397945.Aave_0059 | 5.60E-200 |  | *Acidovorax citrulli* (strain AAC00-1) (*Acidovorax avenae subsp. citrulli)* |  | H | amine oxidase |
| unique/1630/9/Org9_Gene2811 | 266265.Bxe_C1144 | 3.00E-60 | MCHE | *Paraburkholderia xenovorans* (strain LB400) | K02022, K13408 | U | secretion protein |
| unique/1664/9/Org9_Gene2049 | 1064539.AZOBR_140297 | 1.00E-41 |  | Azospirillum brasilense Sp245 | K06921 | S | Archaeal ATPase |
| unique/2107/9/Org9_Gene71 | 596151.DesfrDRAFT_0169 | 2.00E-60 |  | *Desulfovibrio fructosivorans* |  |  |  |
| unique/2118/9/Org9_Gene2808 | 661367.LLO_2903 | 9.90E-28 |  | *Legionella longbeachae* serogroup 1 (strain NSW150) |  | S | Inherit from proNOG: RimK domain protein ATP-grasp |
| unique/2465/9/Org9_Gene3667 | 391612.CY0110_22834 | 4.60E-08 | BMUL_4716 | *Cyanothece* sp. CCY0110 |  | S | aaa ATPase |
| unique/2512/9/Org9_Gene3599 | 190486.XAC4279 | 2.50E-91 |  | *Xanthomonas axonopodis pv. citri* (strain 306) |  |  |  |
| unique/2544/9/Org9_Gene280 | 266835.mlr4370 | 3.90E-07 |  | *Rhizobium loti* (strain MAFF303099) (*Mesorhizobium loti)* |  |  |  |
| unique/2545/9/Org9_Gene1779 | 1045855.DSC_06280 | 2.50E-14 | GUMN | *Pseudoxanthomonas spadix* (strain BD-a59) |  | S | gumn family |
| unique/2677/9/Org9_Gene278 | 426355.Mrad2831_3035 | 2.40E-22 |  | *Methylobacterium radiotolerans* (strain ATCC 27329 / DSM 1819 / JCM 2831) |  |  |  |
| unique/2955/9/Org9_Gene3691 | 517433.PanABDRAFT_0490 | 3.90E-99 | LYSR18 | Pantoea sp. aB | K18900 | K | lysr family transcriptional regulator |
| unique/3060/9/Org9_Gene3684 | 398578.Daci_5638 | 7.90E-121 |  | *Delftia acidovorans* (strain DSM 14801 / SPH-1) |  | K | transcriptional Regulator LysR family |
| unique/3243/9/Org9_Gene1292 | 312153.Pnuc_0323 | 1.50E-41 |  | *Polynucleobacter asymbioticus* (strain DSM 18221 / CIP 109841 / QLW-P1DMWA-1) *(Polynucleobacter necessarius* subsp. asymbioticus) |  | Q | Phytanoyl-CoA dioxygenase (PhyH) |
| unique/3291/9/Org9_Gene194 | 1045855.DSC_02425 | 2.20E-80 | WZM | *Pseudoxanthomonas spadix* (strain BD-a59) | K01992,K09690 | V | transporter |
| unique/3461/9/Org9_Gene1750 | 350701.BDAG_03369 | 5.50E-33 |  | *Burkholderia dolosa* AU0158 | K15540 | O | Pili assembly chaperone |
| unique/3615/9/Org9_Gene3596 | 377431.CferDRAFT_1902 | 1.60E-24 |  | *Chlorobium ferrooxidans* DSM 13031 |  | S | Inner membrane protein YmfA |
| unique/3674/9/Org9_Gene3685 | 339670.Bamb_3531 | 2.20E-111 |  | *Burkholderia ambifaria* (strain ATCC BAA-244 / AMMD) (*Burkholderia cepacia* (strain AMMD)) |  | S | Short-chain dehydrogenase reductase sdr |
| unique/3717/9/Org9_Gene1753 | 340100.Bpet4466 | 6.60E-44 | ECPD | *Bordetella petrii (*strain ATCC BAA-461 / DSM 12804 / CCUG 43448) | K07346,K07357,K15540 | O | Pili assembly chaperone |
| unique/3739/9/Org9_Gene1742 | 743721.Psesu_2031 | 3.10E-30 | ECPD | *Pseudoxanthomonas suwonensis* (strain | K07346,K07357,K15540 | N, U | Pfam:Pili_assembly_C |
| unique/3766/9/Org9_Gene1229 | 441620.Mpop_2731 | 1.00E-60 | DCM | *Methylobacterium populi* (strain ATCC BAA-705 / NCIMB 13946 / BJ001) | K00558 | L | C-5 cytosine-specific DNA methylase |
| unique/3822/9/Org9_Gene1745 | 340100.Bpet4466 | 1.90E-32 | ECPD | *Bordetella petrii* (strain ATCC BAA-461 / DSM 12804 / CCUG 43448) 250 | K07346, K07357,K15540 | O | Pili assembly chaperone |
| unique/3900/9/Org9_Gene3545 | 160488.PP_1958 | 5.30E-19 |  | *Pseudomonas putida* (strain ATCC 47054 / DSM 6125 / NCIMB 11950 / KT2440) |  | S | Protein of unknown function (DUF2026) |
| unique/3947/9/Org9_Gene1595 | 216591.BCAM1881 | 4.80E-81 |  | *Burkholderia cenocepacia* (strain ATCC BAA-245 / DSM 16553 / LMG 16656 / NCTC 13227 / J2315 / CF5610) (*Burkholderia cepacia* (strain J2315)) |  |  |  |
| unique/4040/9/Org9_Gene3694 | 78245.Xaut_0790 | 2.40E-32 |  | *Xanthobacter autotrophicus* (strain ATCC BAA-1158 / Py2) |  |  |  |
| unique/4105/9/Org9_Gene3686 | 267608.RSc2594 | 3.20E-74 | GSTCH1 | *Ralstonia solanacearum* (strain GMI1000) (*Pseudomonas solanacearum)* | K00799, K03599 | O | Glutathione S-transferase, C-terminal domain |
| unique/4222/9/Org9_Gene293 | 399742.Ent638_0318 | 2.30E-16 | Y0567 | *Enterobacter* sp. (strain 638) | K12678 | M, U | outer membrane autotransporter |
| unique/4263/9/Org9_Gene1596 | 216591.BCAM1882 | 2.90E-48 |  | *Burkholderia cenocepacia* (strain ATCC BAA-245 / DSM 16553 / LMG 16656 / NCTC 13227 / J2315 / CF5610) (Burkholderia cepacia (strain J2315)) |  |  |  |
| unique/4294/9/Org9_Gene3662 | 500635.MITSMUL_03861 | 1.40E-31 |  | *Mitsuokella multacida* DSM 20544 |  | S | phosphoglycerate mutase family protein |
| unique/4306/9/Org9_Gene79 | 316273.XCV2685 | 3.50E-86 |  | *Xanthomonas campestris pv. vesicatoria* (strain 85-10) |  | S | Protein of unknown function (DUF1629) |
| unique/4512/9/Org9_Gene3687 | 640512.BC1003_0658 | 2.20E-74 | LDB1079 | *Burkholderia* sp. (strain CCGE1003) |  | L | integrase family |
| unique/4635/9/Org9_Gene1588 | 1007104.SUS17_1730 | 1.50E-27 | HEMO |  | K07215 | P | heme oxygenase |
| unique/4821/9/Org9_Gene1754 | 266265.Bxe_C1153 | 3.90E-09 | FIMA | *Paraburkholderia xenovorans* (strain LB400) | K07345 | N, U | Fimbrial |
| unique/4902/9/Org9_Gene3475 | 640513.Entas_1928 | 1.50E-58 |  | *Enterobacter asburiae* (strain LF7a) |  | S | Protein of unknown function (DUF3304) |
| unique/5072/9/Org9_Gene1746 | 626418.bglu_1g22890 | 2.30E-08 | FIMA | *Burkholderia glumae* (strain BGR1) | K07345, K12517 | N, U | Inherit from proNOG: Fimbrial protein |
| unique/5251/9/Org9_Gene3597 | 438753.AZC_2823 | 7.60E-09 | YMFA | *Azorhizobium caulinodans* (strain ATCC 43989 / DSM 5975 / JCM 20966 / NBRC 14845 / NCIMB 13405 / ORS 571) |  | S | Protein of unknown function (DUF3592) |
| unique/5302/9/Org9_Gene3693 | 1001585.MDS_3582 | 3.90E-42 |  | *Pseudomonas mendocina* (strain NK-01) | K09936 | S | Protein of unknown function, DUF606 |
| unique/5342/9/Org9_Gene3167 | 743721.Psesu_1124 | 1.60E-56 | INTE | *Pseudoxanthomonas suwonensis* (strain 11-1) |  | L | Integrase |
| unique/5463/9/Org9_Gene1535 | 471874.PROSTU_03721 | 6.00E-24 | HOCA | *Providencia stuartii* ATCC 25827 |  | S | Inherit from proNOG: cytoplasmic protein |
| unique/5542/9/Org9_Gene8 | 944559.HMPREF9413_1219 | 6.30E-10 |  | *Paenibacillus* sp. HGF7 |  |  |  |
| unique/5626/9/Org9_Gene1747 | 522373.Smlt0732 | 1.60E-10 | YEHC | *Stenotrophomonas maltophilia* (strain K279a) | K07346, K15540 | N, U | Chaperone |
| unique/5930/9/Org9_Gene1134 | 522373.Smlt1748 | 1.80E-48 | ACEE | *Stenotrophomonas maltophilia* (strain K279a) | K00163 | C | Component of the pyruvate dehydrogenase (PDH) complex, that catalyzes the overall conversion of pyruvate to acetyl-CoA and CO(2) (By similarity) |
| unique/6046/9/Org9_Gene2084 | 383407.Xoryp_010100020185 | 2.40E-34 |  | *Xanthomonas oryzae pv. oryzicola* (strain BLS256) | K02004 | V | ABC, transporter |
| unique/6351/9/Org9_Gene1228 | 441620.Mpop_2731 | 3.00E-12 | DCM | *Methylobacterium populi* (strain ATCC BAA-705 / NCIMB 13946 / BJ001) | K00558 | L | C-5 cytosine-specific DNA methylase |
| unique/6366/9/Org9_Gene3674 | 543728.Vapar_2066 | 1.10E-27 |  | *Variovorax paradoxus* (strain S110) | K02014 | P | receptor |
| unique/6512/9/Org9_Gene1238 | 1127134.NOCYR_5259 | 3.80E-09 |  | *Nocardia cyriacigeorgica* (strain GUH-2) |  | K | Helix-turn-helix |
| unique/6685/9/Org9_Gene3291 | 384676.PSEEN3073 | 2.10E-19 |  | Pseudomonas entomophila (strain L48) |  | S | Colicin D |
| unique/6712/9/Org9_Gene1237 | 522373.Smlt1978 | 1.70E-48 |  | *Stenotrophomonas maltophilia* (strain K279a) |  | S | conserved protein |
| unique/6925/9/Org9_Gene3029 | 391008.Smal_3635 | 6.20E-15 |  | *Stenotrophomonas maltophilia* (strain R553-1) | K06996 | S | glyoxalase bleomycin resistance protein dioxygenase |
| unique/6990/9/Org9_Gene3712 | 522373.Smlt0173 | 5.20E-19 |  | *Stenotrophomonas maltophilia* (strain K279a) |  |  |  |
| unique/7079/9/Org9_Gene2048 | 264730.PSPPH_2283 | 2.70E-14 |  | *Pseudomonas savastanoi pv. phaseolicola* (strain 1448A / Race 6) (*Pseudomonas syringae pv. phaseolicola* (strain 1448A / Race 6)) |  | S | Suppressor of fused protein (SUFU) |
| unique/7389/9/Org9_Gene3161 | 391008.Smal_2797 | 1.20E-07 |  | *Stenotrophomonas maltophilia* (strain R553-1) |  | S | acetyltransferase, GNAT family |

**Table S5 Comparison of COGs in genomes between *Stenotrophomonas* sp Pemsol and reported hydrocarbon degrading bacteria**

| Func_id | Func_name | Stenotrophomonas_sp_PEMSOL_geneCount | Acinetobacter_baylyi_ADP1_geneCount | Acinetobacter_lwoffii_SH145_geneCount | Alcanivorax_borkumensis_SK2_geneCount | Franconibacter_pulveris_DJ34_geneCount |
| --- | --- | --- | --- | --- | --- | --- |
| A | RNA processing and modification | 1 | 1 | 1 | 1 | 1 |
| B | Chromatin structure and dynamics | 1 | 0 | 0 | 1 | 0 |
| C | Energy production and conversion | 188 | 179 | 150 | 162 | 223 |
| D | Cell cycle control, cell division, chromosome partitioning | 34 | 27 | 28 | 33 | 42 |
| E | Amino acid transport and metabolism | 210 | 234 | 172 | 160 | 389 |
| F | Nucleotide transport and metabolism | 72 | 88 | 69 | 64 | 95 |
| G | Carbohydrate transport and metabolism | 144 | 108 | 72 | 64 | 404 |
| H | Coenzyme transport and metabolism | 150 | 162 | 132 | 148 | 204 |
| I | Lipid transport and metabolism | 134 | 186 | 145 | 162 | 130 |
| J | Translation, ribosomal structure and biogenesis | 213 | 202 | 204 | 205 | 238 |
| K | Transcription | 232 | 189 | 128 | 135 | 337 |
| L | Replication, recombination and repair | 101 | 82 | 96 | 91 | 123 |
| M | Cell wall/membrane/envelope biogenesis | 210 | 163 | 131 | 164 | 254 |
| N | Cell motility | 116 | 34 | 32 | 36 | 124 |
| O | Posttranslational modification, protein turnover, chaperones | 146 | 104 | 102 | 118 | 154 |
| P | Inorganic ion transport and metabolism | 180 | 180 | 146 | 141 | 226 |
| Q | Secondary metabolites biosynthesis, transport and catabolism | 80 | 80 | 46 | 62 | 79 |
| R | General function prediction only | 261 | 260 | 194 | 202 | 300 |
| S | Function unknown | 200 | 140 | 116 | 141 | 207 |
| T | Signal transduction mechanisms | 205 | 101 | 89 | 120 | 201 |
| U | Intracellular trafficking, secretion, and vesicular transport | 75 | 59 | 49 | 46 | 98 |
| V | Defense mechanisms | 99 | 68 | 63 | 49 | 78 |
| W | Extracellular structures | 55 | 25 | 21 | 28 | 26 |
| X | Mobilome: prophages, transposons | 13 | 16 | 28 | 8 | 19 |
| Z | Cytoskeleton | 1 | 0 | 0 | 1 | 1 |

**Table S6 Genonme-Genome Distance calculated Digital DNA Hybridization (DDH) value for Pemsol with the closely related strain *Stenotrophomonas maltophila* K279a as the reference**
