# Supplementary material for: The Polycyclic Aromatic Hydrocarbon (PAH) degradation activities and genome analysis of a novel strain Stenotrophomonas sp. Pemsol isolated from Mexico
Source: PeerJ. 2020 Jan 6;8:e8102. doi: 10.7717/peerj.8102 (PMC6951288; doi:10.7717/peerj.8102)
Supplement: Supplemental Information 7 — Fig. 1 Phylogenetic tree of Stenotrophomonas sp. Pemsol with other members of the genus Stenotrophomonas based on the sequence of 16S rRNA gene, Fig. 2:Pemsols growth in solid media with PAH as sole carbon source Fig. 3 contains FTIR spectrum while Figs. 3 and 4 contain UPLC MS spectra, Fig. 6 Catechol 2, 3 dioxygenase containing region in Pemsol [file peerj-08-8102-s007.pdf]

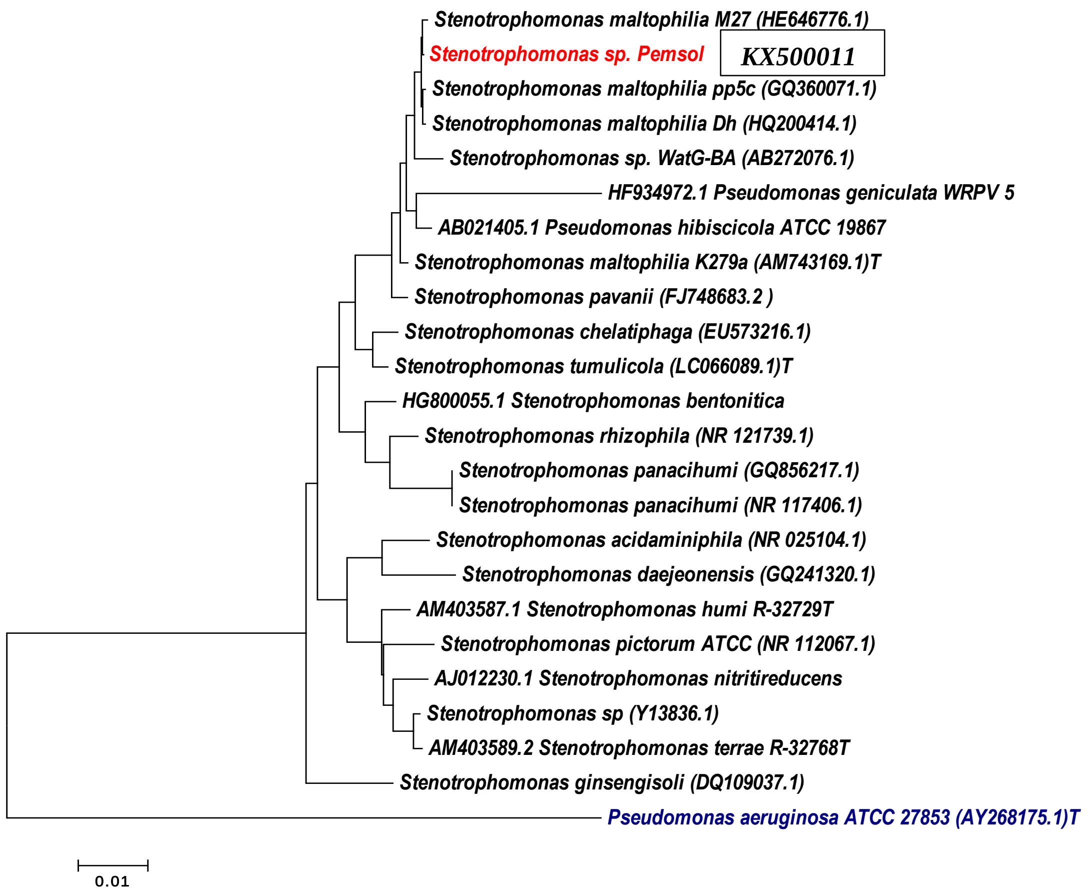

Figure S1. Phylogenetic tree of *Stenotrophomonas sp*. Pemsol with other members of the genus *Stenotrophomonas* based on the sequence of 16S rRNA gene.

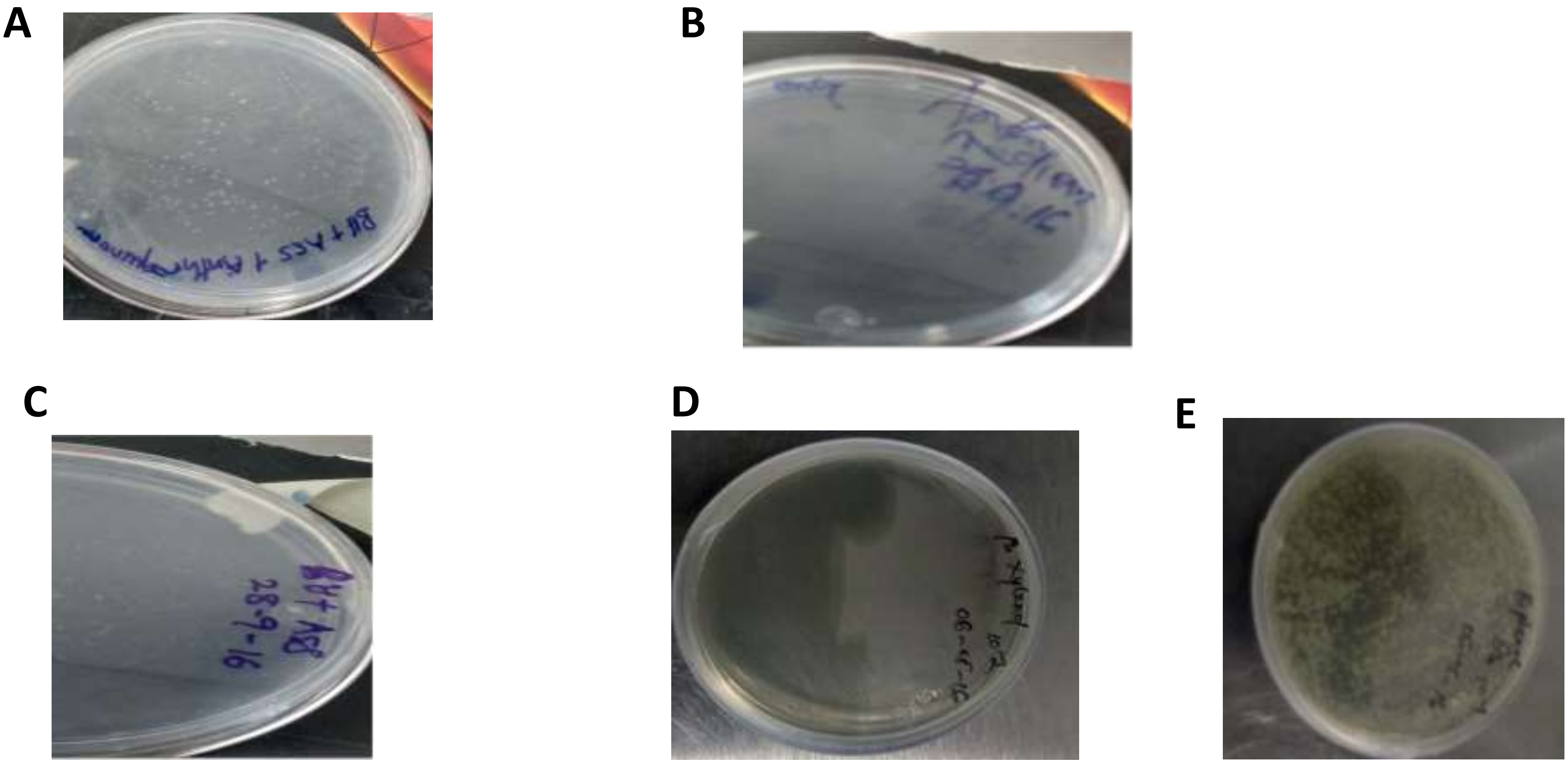

A Pemsol's growth in PAH (anthroquinone)

B PAH in minaml medium

C: Pemsol in minimal medium without PAH

D: Pemsol unable to grow in Xylene

E. Pemsol colony count from spread plate technique on luria agar after 48 hours growth in minimal medium with biphenyl as sole carbon source.

Figure S2: Pemsols growth in solid media with PAH as sole carbon source

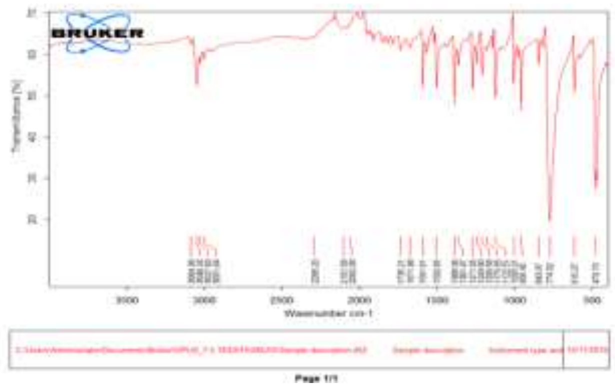

a, **Image from the FTIR spectrometry for naphthalene degradation**

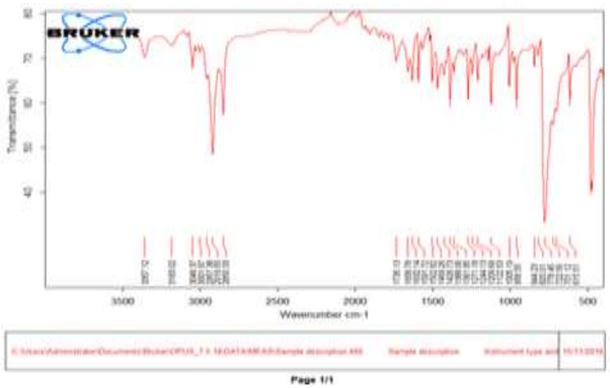

b, FTIR spectrometry for naphthalene metabolites formed after the 15th day of

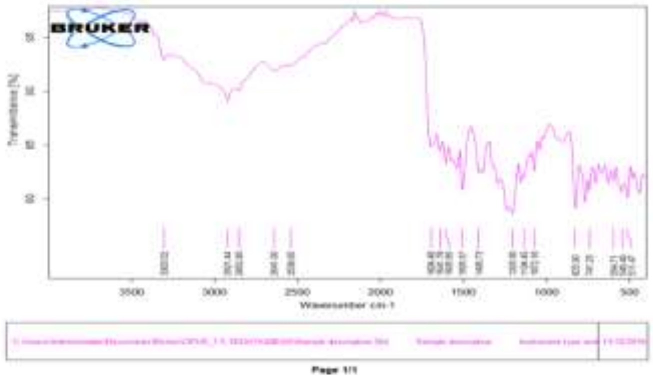

c, Image from the FTIR spectrometry of the metabolite formed from degradtion of naphthalene by *Stenotrophomona*s sp. Pemsol at 30th day.

Figure S3: FTIR spectrometry analysis of the degradation metabolites formed from the activities of Pemsol on naphthalene and the controls

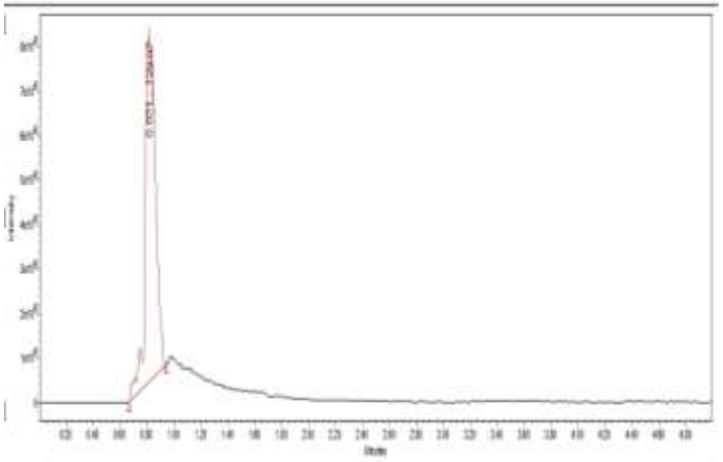

A

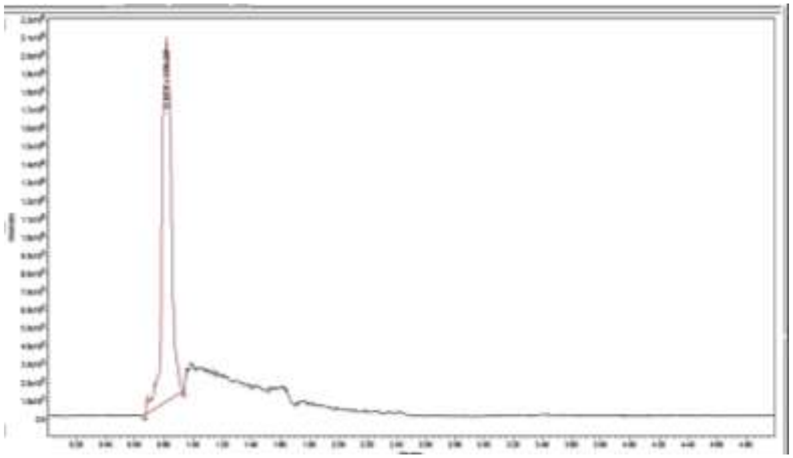

B

The UPLC-MS analysis spectrum of the control experiment after the 15th day of degradation study B UPLC-MS analysis spectrum of the test experiment after the 15th day of degradation studies

Figure S4: UPLC mass spectrometry image for the metabolites formed from the degradation of naphthalene by *Stenotrophomonas* sp.

Pemsol after 15th day of degradation study.

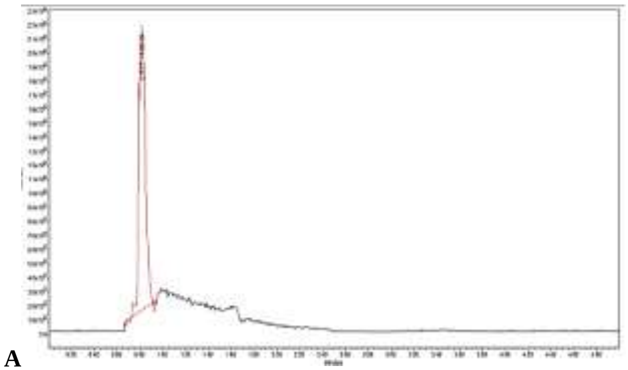

A

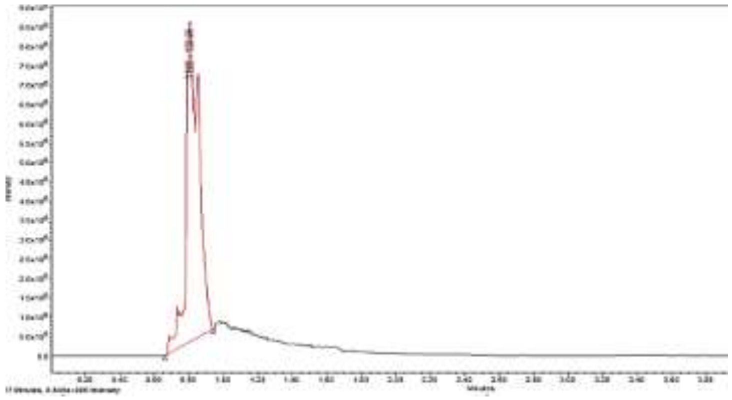

B

Figure S5 UPLC mass spectrometry image for the metabolites formed from the degradation of Naphthalene by *Stenotrophomonas* sp. Pemsol within 30 days of degradation study.

A UPLC-MS spectrum for the metabolite formed from the degradation of naphthalene by *Stenotrophomonas* sp. Pemsol

B UPLC-MS spectrum for naphthalene in the control experiment after 30 days of experimental study on *Stenotrophomonas* species Pemsol's degradation of naphthalene.

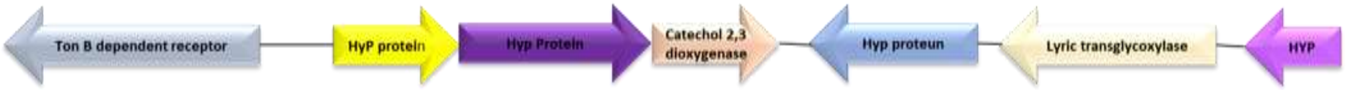

Figure S6: Catechol 2, 3 dioxygenase containing region in Pemsol
